# Rational design of biodegradable sulphonamide candidates treating septicaemia by synergistic dual inhibition of COX-2/PGE2 axis and DHPS enzyme

**DOI:** 10.1080/14756366.2022.2086868

**Published:** 2022-06-16

**Authors:** Nada H. El-Dershaby, Soad A. El-Hawash, Shaymaa E. Kassab, Hoda G. Dabees, Ahmed E. Abdel Moneim, Ibrahim A. Abdel Wahab, Mohammad M. Abd-Alhaseeb, Mostafa M. M. El-Miligy

**Affiliations:** a Pharmaceutical Chemistry Department, Faculty of Pharmacy, Damanhour University, Damanhour, Egypt; b Pharmaceutical Chemistry Department, Faculty of Pharmacy, Alexandria University, Alexandria, Egypt; c Department of organic and Medicinal Chemistry, Faculty of Pharmacy, University of Sadat City, Menoufia,Egypt; d Department of Zoology and Entomology, Faculty of Science, Helwan University, Cairo, Egypt; e Microbiology and Immunology Department, Faculty of Pharmacy, Pharos University in Alexandria, Alexandria, Egypt; f Department of Pharmacology and Toxicology, Faculty of Pharmacy, Damanhur University, Damanhour, Egypt

**Keywords:** Sulphonamides, salicylamides, COX-2 inhibitors, PGE2, septicaemia

## Abstract

A new series of co-drugs was designed based on hybridising the dihydropteroate synthase (DHPS) inhibitor sulphonamide scaffold with the COX-2 inhibitor salicylamide pharmacophore through biodegradable linkage to achieve compounds with synergistic dual inhibition of COX-2/PGE2 axis and DHPS enzyme to enhance antibacterial activity for treatment of septicaemia. Compounds **5 b, 5j, 5n** and **5o** demonstrated potent *in vitro* COX-2 inhibitory activity comparable to celecoxib. **5j** and **5o** exhibited ED_50_ lower than celecoxib in carrageenan-induced paw edoema test with % PGE2 inhibition higher than celecoxib. Furthermore, **5 b**, **5j** and **5n** showed gastric safety profile like celecoxib. Moreover, *in vivo* antibacterial screening revealed that, **5j** showed activity against *S.aureus and E.coli* higher than sulfasalazine. While, **5o** revealed activity against *E.coli* higher than sulfasalazine and against *S.aureus* comparable to sulfasalazine. Compound **5j** achieved the target goal as potent inhibitor of COX-2/PGE2 axis and *in vivo* broad-spectrum antibacterial activity against induced septicaemia in mice.

## Graphical Abstract

**Figure:**
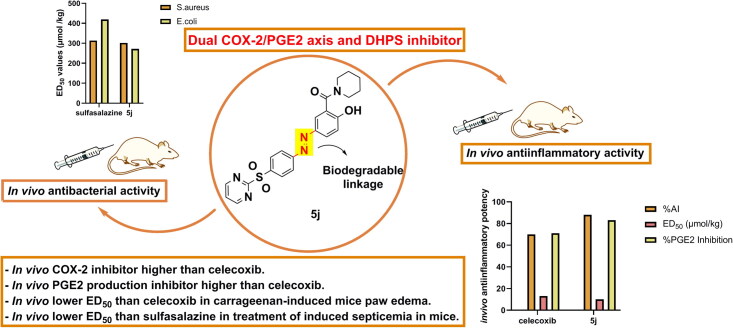

## 1. Introduction

**Figure 1.**
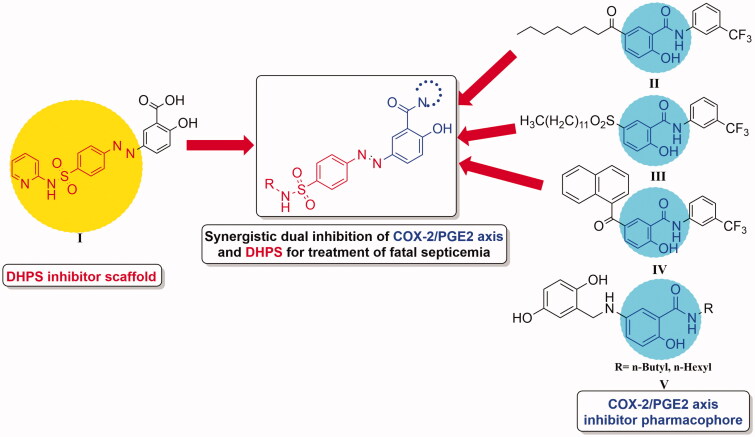
Rational design of dual COX-2/PGE2 axis and DHPS inhibitors.

Prostaglandin E2 (PGE2) is an oxygenated arachidonic acid metabolite produced mainly by the action of Cyclooxygenase enzymes (COX)^1^. Being a pro-inflammatory mediator, PGE2 activates macrophages, neutrophils, and mast cells at early stages of inflammation^2^^,^^3^. Additionally, it had been demonstrated as a powerful immunosuppressant inhibiting pathogen-killing *via* alveolar macrophages and phagocytosis. As a result, it is considered a potent risk factor during inflammation^4^^,^^5^. Moreover, COX-2 isozyme, a key enzyme in the process of PGE2 biosynthesis, had been reported to be induced by infections of the pathogenic *Streptococcus pyogenes*. In addition, several bacterial toxins including *streptococcal cytolysin S* (SLS) and *streptococcal cytolysin O* (SLO) produced by *S. pyogenes* together with *pneumolysin* and *Clostridium difficile toxin A* have been reported as potent inducers of COX-2 expression^6^. besides, it had been confirmed that COX-2/PGE2 production were up-regulated and promoted biofilm formation by *Staphylococcus aureus* thereby enhances its adherence to the human fibronectin and finally motivates its growth^7^. Hence, blocking COX-2/PGE2 pathway introduces a new, an effective and an unexpected manner to overcome bacterial infections. Accordingly, celecoxib, the selective COX-2 inhibitor, had been reported to increase sensitivity of *Staphylococcus aureus* to antibiotics^8^. Interestingly, celecoxib increased the utilisation of ampicillin by bacteria by inhibiting antibiotic resistance genes as well as increasing membrane permeability^9^^,^^10^. Furthermore, it had been reported that the commonly available antibiotics chloramphenicol, cefuroxime and Oxytetracycline exerted a synergistic effect with celecoxib, against MRSA strains with a 4-fold reduction in the cefuroxime and chloramphenicol MICs and 2-fold decrease in the Oxytetracycline MIC^11^^,^^12^^,^^13^. On the other hand, sulphonamides were the first antibacterial agents to be discovered by Gerhard Domagk, since then they have been applied for different clinical indications^14^. Moreover, they exert their action by competitive inhibition of dihydropteroate synthase enzyme (DHPS), a significant anti-folate target^15^. Additionally, the sulphonamide moiety constitutes the prime pharmacophore of most selective COX-2 inhibitors as celecoxib and its derivatives^16^. In fact, sulphonamides comprise an interesting class of drugs with a wide scope of pharmacological activities such as antibacterial^17^^,^^18^, hypoglycemic^19^, diuretic^20^, carbonic anhydrase enzyme inhibitory properties^21–24^, antithyroid^25^ and anti-inflammatory agents^26^. This may be attributed to sulphonamides' phenylamino and sulphonyl amino groups essential for various biological activities as well as metal coordination^27^. Furthermore, Sulfasalazine (**I**), Figure 1 is a sulphonamide prodrug degraded by colonic bacteria via azo reductase enzyme to sulfapyridine, an antibiotic, and 5-aminosalicylic acid, an anti-inflammatory agent, compounds with different modes of action^28^^,^^29^. It has been demonstrated that, sulfasalazine exerted its anti-inflammatory action *via* inhibiting a number of immunological processes, including lymphocyte proliferation, interleukin-2 synthesis in addition to interleukin-1 production by monocytes, but was not classified as an NSAID^30^. On the other hand, salicylamides had been previously reported as inhibitors of the two-component regulatory system (TCS) in bacteria^31^^,^^32^. Besides, "5-substituted salicylamides" mainly designed to act as topical antibiotics, were serendipitously discovered to have innate anti-inflammatory activity. Therefore, those 5-substituted salicylamides as were referred to as lipophilic, antibacterial, anti-inflammatory drugs (LAADs). Salifluor (**II**), Figure 1, the first-generation lead drug candidate of LAADs, had an excellent efficacy against a wide variety of Gram-positive and Gram-negative oral organisms. Trifluorosal (**III**), Figure 1, a second-generation LAAD-type salicylamide, underwent early-stage pharmaceutical development for gingivities treatment. Among the third generation, naphthafluor (**IV**), Figure 1, is currently in development as a treatment for acne as well as gingivitis^33^. Besides, salicylamide derivatives **V,** Figure 1 exhibited significant COX-2 inhibition with significant COX-2 selectivity comparable to celecoxib^34^. As well, salicylamide derivatives have been regarded as one of the promising candidates owing to their interesting biological activities, including antimycobacterial^35^^,^^36^, antifungal^37^, antibacterial^31^^,^^38^, antineoplastic^39^^,^^40^, antianthelminthic^41^, antiparasitic^42^ as well as anti-inflammatory activities^43^^,^^44^. In view of the aforesaid facts, designing new candidates targeting COX-2/DHPS enzymes together with PGE2 production would be more effective in combating inflammatory bacterial infections. Thus, in the current study, we utilised the DHPS inhibiting properties of sulphonamides and the COX-2 inhibiting properties of salicylamides as well as their beneficial antibacterial effects to provide a monotherapy approach targeting COX-2 and DHPS enzymes being effective against bacterial infections together with their sequential inflammatory actions with lower evidence of resistance. Accordingly, herein we designed some new biodegradable molecules combining sulphonamide moiety linked to *N*-Substituted-2-hydroxybenzamides through azo linkage. The newly synthesised compounds were evaluated *in vitro* for their COX-1/COX-2 inhibitory activities and their antibacterial activities against human pathogenic bacteria including a number of Gram-positive and Gram-negative bacteria and the most active compounds were further evaluated *in vivo* for their anti-inflammatory and antibacterial efficacies. In addition, their effect on PGE2 production and ulcerogenic effect were also evaluated. Moreover, molecular docking studies were performed to profile the binding pattern of the potential dual COX-2/DHPS inhibitors with the active site of the targeted COX-2 and dihydropteroate synthase (DHPS) enzymes. Furthermore, *in silico* predictions of physicochemical parameters, drug likeness score and acquiescence to the Lipinski’s rule of five (RO5) were carriedout to the biologically active compounds to estimate their suitability to act as possible orally active dug candidates.

## 2. Experimental

### 2.1. Chemistry

All chemicals were bought from commercial sources and used directly without further purification. Open-glass capillaries were used in measuring melting points on Stuart Scientific melting point apparatus (SMP10) and are uncorrected. The reactions' rates were followed up by thin layer chromatography (TLC) on silica gel pre-coated Merck aluminium GF254 plates, and the spots were visualised at λ 254 nm using UV-lamp for few seconds' exposure to iodine vapours. Thermo Scientific Nicolet iS20 Fourier Transform Infra-red Spectrometer was used for recording Infra-red spectra (IR), using KBr discs, Faculty of Pharmacy, Mansoura University. Proton nuclear magnetic resonance spectra (^1^H-NMR) were scanned on JNM-ECA II 500 MHz JEOL Spectrometer at the NMR unit, Faculty of Science, Mansoura University and on Bruker Avance III 400 MHz Spectrometer at Centre for Drug Discovery Research and Development-Faculty of Pharmacy, Ein Shams University using deuterated dimethylsulphoxide (DMSO-d_6_) as a solvent. Data were interpreted as chemical shifts expressed in δ values (ppm) relative to tetramethylsilane (TMS) as internal standard. Signal splitting type was indicated by one of the following letters: s = singlet, d = doublet, t = triplet, q = quartette, dd = doublet of doublet and m = multiplet. ^13 ^C-NMR spectra were scanned on JNM-ECA II 125 MHz JEOL Spectrometer at the NMR unit, Faculty of Science, Mansoura University. Electron impact mass spectra (EI-MS) were scanned on Direct Inlet part mass analyser in Thermo Scientific GCMS model ISQ at the Regional Centre for Mycology and Biotechnology, Al-Azhar University, Cairo. Elemental analyses (C, H, N and S) was the tool used for checking purity of the new compounds using FLASH 2000 CHNS/O analyser, Thermo Scientific at the Regional Centre for Mycology and Biotechnology (RCMB), Al-Azhar University. The results were within ±0.4% of the calculated values for the proposed formulae. ChemDraw Professional 16.0.1.4. was used for naming the new compounds according to the naming algorithm developed by CambridgeSoft Corporation compounds **2a**^45^ and **2 b-2d**^46^^,^^47^^,^^48^ were prepared as previously reported.

#### 2.1.1. General procedure for the synthesis of compounds 5a-o

The appropriate sulphonamide derivative (2 mmol) was suspended in hydrochloric acid (17%, 2 ml) then ethanol was added until a clear solution was obtained the solution was cooled from 0 °C to −5 °C and a solution of NaNO2 (2 mmol, 0.138 gm) in water (1 ml) was added dropwise. After stirring at 0 °C for 15–30 min, a solution of the appropriate N-substituted-2-hydroxybenzamide (2 mmol) in NaOH (10%, 2 ml) was added to the diazonium salt dropwise and the reaction mixture was stirred overnight at room temperature. The formed precipitate was filtered off, washed with water and crystallised from ethanol.

##### 2.1.1.1. N-Butyl-2-hydroxy-5-((4-sulfamoylphenyl) diazenyl) benzamide (5a)

Pale brown crystals; yield 95%, m.p. 227–229 °C. IR (KBr, cm^−1^): 3529 (OH), 3377, 3269 (NH_2_, NH), 1646 (C = O), 1496 (N = N), 1305, 1149 (SO_2_). ^1^H-NMR (400 MHz, DMSO-d6) δ 0.92 (*t*, *J* = 7.4 Hz, 3H, NH-CH_2_-CH_2_-CH_2_-CH_3_), 1.31–1.42 (*m*, 2H, NH-CH_2_-CH_2_-CH_2_-CH_3_), 1.52–1.61 (*m*, 2H, NH-CH_2_-CH_2_-CH_2_-CH_3_), 3.36–3.39 (*m*, 2H, NH-CH_2_-CH_2_-CH_2_-CH_3_), 7.08 (d, *J* = 8.6 Hz, 1H, salicyl-C_3_-H),7.51 (*s*, 2H, sulfamoylphenyl NH_2_, D_2_O exchangeable), 7.78–7.82 (*m*, 4H, 1-sulfamoylphenyl-C_2,6,3,5_-H), 7.97 (dd, *J* = 8.6, 2.4 Hz, 1H, salicyl-C_4_-H), 8.57 (d, *J* = 2.4 Hz, 1H, salicyl-C_6_-H), 9.14 (*t*, *J* = 5.7 Hz, 1H, CH_2_-NH, D_2_O exchangeable), 13.48 (*s*, 1H, OH, D_2_O exchangeable) 0.13^ ^C-NMR (125 MHz, DMSO-D_6_) δ 13.72 (NH-CH_2_-CH_2_-CH_2_-CH_3_), 19.67 (NH-CH_2_-CH_2_-CH_2_-CH_3_), 30.82 (NH-CH_2_-CH_2_-CH_2_-CH_3_), 46.47 (NH-CH_2_-CH_2_-CH_2_-CH_3_), 115.31(salicyl-C_3_), 118.68 (salicyl-C_1_), 121.79 (sulfamoylphenyl-C_2,6_), 125.44 (salicyl-C_4_), 125.83(salicyl-C_6_), 126.77 (sulfamoylphenyl-C_3,5_), 144.49 (sulfamoylphenyl-C_4_), 150.34 (salicyl-C_5_), 151.68 (sulfamoylphenyl-C_1_), 163.42 (salicyl-C_2_), 168.58 (C = O). Anal. Calcd.for C_17_H_20_N_4_O_4_S (376.43): C, 54.24; H, 5.36; N, 14.88; S, 8.52. Found: C, 54.37; H, 5.48; N, 14.66; S, 8.63.

##### 2.1.1.2. N-Butyl-2-hydroxy-5-((4-(N-(thiazol-2-yl) sulfamoyl) phenyl) diazenyl) benzamide (5 b)

Shiny orang crystals; yield 97%, m.p. 239–241 °C. IR (KBr, cm^−1^): 3528 (OH), 3395, 3373 (NH), 1637 (C = O), 1491 (N = N), 1298, 1143 (SO_2_). ^1^H-NMR (400 MHz, DMSO-d6) δ 0.91 (*t*, *J* = 7.5 Hz, 3H, NH-CH_2_-CH_2_-CH_2_-CH_3_), 1.30–1.40 (*m*, 2H, NH-CH_2_-CH_2_-CH_2_-CH_3_), 1.52–1.60 (*m*, 2H, NH-CH_2_-CH_2_-CH_2_-CH_3_), 3.41–3.47 (*m*, 2H, NH-CH_2_-CH_2_-CH_2_-CH_3_), 6.88 (d, *J* = 4.7 Hz, 1H,thiazolyl-C_5_-H), 7.10 (d, *J* = 8.9 Hz, 1H, salicyl-C_3_-H), 7.29 (d, *J* = 4.7 Hz, 1H,thiazolyl-C_4_-H), 7.94 − 8.01 (*m*, 5H, 1-sulfamoyl phenyl-C_2,3,5,6_-H and salicyl-C_4_-H), 8.59 (*s*, 1H, salicyl-C_6_-H), 9.12 (*t*, *J* = 5.7 Hz, 1H, CH_2_-NH, D_2_O exchangeable), 12.84 (*s*, 1H, sulfamoyl NH, D_2_O exchangeable), 13.52 (*s*, 1H, OH, D_2_O exchangeable). Anal. Calcd.for C_20_H_21_N_5_O_4_S_2_ (459.54): C, 52.27; H, 4.61; N, 15.24; S, 13.95. Found: C, 52.39; H, 4.72; N, 15.13; S, 14.07.

##### 2.1.1.3. N-Butyl-2-hydroxy-5-((4-(N-(pyrimidin-2-yl) sulfamoyl) phenyl) diazenyl) benzamide (5c)

Orang crystals; yield 95%, m.p. 228–230 °C. IR (KBr, cm^−1^): 3653 (OH), 3465, 3425 (NH), 1646 (C = O), 1494 (N = N), 1344,1146 (SO_2_).^1^H-NMR (400 MHz, DMSO-d6) δ 0.91 (*t*, *J* = 7.5 Hz, 3H, NH-CH_2_-CH_2_-CH_2_-CH_3_), 1.31–1.40 (*m*, 2H, NH-CH_2_-CH_2_-CH_2_-CH_3_), 1.52 − 1.60 (*m*, 2H, NH-CH_2_-CH_2_-CH_2_-CH_3_), 3.41–3.48 (*m*, 2H, NH-CH_2_-CH_2_-CH_2_-CH_3_), 7.06 (*t*, *J* = 4.6 Hz, 1H, pyrimidinyl-C_5_-H), 7.11 (d, *J* = 8.8 Hz, 1H, salicyl-C_3_-H), 7.96 − 8.00 (*m*, 3H, 1-sulfamoyl phenyl-C_2,6_-H and salicyl-C_4_-H), 8.17 (d, *J* = 8.6 Hz, 2H, sulfamoyl phenyl-C_3,5_-H), 8.52 (d, *J* = 4.6 Hz, 2H, pyrimidinyl-C_4,6_-H), 8.59 (d, *J* = 2.4 Hz, 1H, salicyl-C_6_-H), 9.12 (*t*, *J* = 5.7 Hz, 1H, CH_2_-NH, D_2_O exchangeable), 12.04 (*s*, 1H, 1-sulfamoyl NH, D_2_O exchangeable), 13.52 (*s*, 1H, OH, D_2_O exchangeable). ^13 ^C-NMR (125 MHz, DMSO-D_6_) δ 13.73 (NH-CH_2_-CH_2_-CH_2_-CH_3_), 19.69 (NH-CH_2_-CH_2_-CH_2_-CH_3_), 30.85 (NH-CH_2_-CH_2_-CH_2_-CH_3_), 46.51 (NH-CH_2_-CH_2_-CH_2_-CH_3_), 115.70 (pyrimidinyl-C_5_), 118.83 (salicyl-C_3_), 122.47 (salicyl-C_1_), 124.07 (sulfamoylphenyl-C_2,6_), 126.15 (salicyl-C_4_), 129.11 (salicyl-C_6_), 137.11(sulfamoylphenyl-C_3,5_) 144.53(sulfamoylphenyl-C_4_), 152.10 (salicyl-C_5_), 154.10 (sulphamoylphenyl-C_1_), 156.79 (pyrimidinyl-C_4,6_), 158.70 (pyrimidinyl-C_2_), 163.97 (salicyl-C_2_), 168.27 (C = O). Anal. Calcd.for C_21_H_22_N_6_O_4_S (454.51): C, 55.50; H, 4.88; N, 18.49; S, 7.05. Found: C, 55.62; H, 4.98; N, 18.37; S, 7.13. EIMS m/z (% relative abundance): 457.96 (2.96) (M^+•^+3), 454.49 (10.42) (M^+•^), 432.78 (18.08), 396.36 (43.52), 327.07 (28.63), 115.05 (38.74), 57.04 (48.32), 44.92 (82.56) 43.03 (100) (base peak).

##### 2.1.1.4. N-Butyl-2-hydroxy-5-((4-(N-(4-methylpyrimidin-2yl) sulfamoyl) phenyl) diazenyl) benzamide (5d)

Deep orang crystals; yield 97%, m.p. 235–238 °C. IR (KBr, cm^−1^): 3676 (OH), 3525, 3394 (NH), 1643 (C = O), 1495 (N = N), 1347, 1149 (SO_2_). ^1^H-NMR (400 MHz, DMSO-d6) δ 0.91 (*t*, *J* = 7.3 Hz, 3H, NH-CH_2_-CH_2_-CH_2_-CH_3_), 1.30 − 1.39 (*m*, 2H, NH-CH_2_-CH_2_-CH_2_-CH_3_), 1.52 − 1.60 (*m*, 2H, NH-CH_2_-CH_2_-CH_2_-CH_3_), 2.32 (*s*, 3H,pyrimidinyl-4-CH_3_), 3.30–3.36 (*m*, 2H, NH-CH_2_-CH_2_-CH_2_-CH_3_), 6.91 (d, *J* = 5.2 Hz, 1H, pyrimidinyl-C_5_-H), 7.11 (d, *J* = 8.9 Hz, 1H, salicyl-C_3_-H), 7.95–7.99 (*m*, 3H, 1-sulfamoyl phenyl-C_2,6_-H, salicyl-C_4_-H), 8.17 (d, *J* = 8.6 Hz, 2H, 1-sulfamoyl phenyl-C_3,5_-H), 8.32 (d, *J* = 5.2 Hz, 1H, pyrimidinyl-C_6_-H), 8.60 (d, *J* = 2.4 Hz, 1H, salicyl-C_6_-H), 9.13 (*t*, *J* = 5.6 Hz, 1H, CH_2_-NH, D_2_O exchangeable), 11.96 (*s*, 1H, sulfamoyl NH, D_2_O exchangeable), 13.53 (*s*, 1H, OH, D_2_O exchangeable). Anal. Calcd.for C_22_H_24_N_6_O_4_S (468.53): C, 56.40; H, 5.16; N, 17.94; S, 6.84. Found: C, 56.52; H, 5.17; N, 17.83; S, 6.94.

##### 2.1.1.5. N-Benzyl-2-hydroxy-5-((4-sulfamoylphenyl) diazenyl) benzamide (5e)

Orang crystals; yield 95%, m.p. 250–252 °C. IR (KBr, cm^−1^): 3452 (OH), 3400, 3361 (NH_2_), 3268 (NH), 1640 (C = O), 1494 (N = N), 1337, 1150 (SO_2_). ^1^H- NMR (400 MHz, DMSO-d_6_) δ 4.56 (*s*, 2H, -benzyl-CH_2_), 7.12 (d, *J* = 9.0 Hz, 1H, salicyl-C_3_-H), 7.28 (*s*, 2H, benzyl-C_2,6_-H), 7.33–7.37 (*m*, 3H, benzyl-C_3,4,5_-H),7.53 (*s*, 2H, sulfamoylphenylNH_2,_ D_2_O exchangeable), 7.97–8.03 (*m*, 5H, sulfamoylphenyl-C_2,3,5,6_-H and salicyl-C_4_-H), 8.64 (*s*, 1H, salicyl-C_6_-H), 9.71 (*s*, 1H, CH_2_-NH, D_2_O exchangeable), 13.30 (br.s, 1H, OH, D_2_O exchangeable) 0.13^ ^C-NMR (125 MHz, DMSO-d_6_) δ 42.65 (benzyl-CH_2_), 116.13 (salicyl-C_3_), 118.95 (salicyl-C_1_), 122.66 (sulfamoylphenyl-C_2,6_), 126.06 (salicyl-C_4_), 126.60 (salicyl-C_6_), 127.09 (benzyl-C_2,6_), 127.49 (benzyl-C_4_), 128.49 (benzyl-C_3,5_), 138.82 (sulfamoylphenyl-C_3,5_), 144.46 (benzyl-C_1_), 145.40 (sulfamoylphenyl-C_4_), 152.00 (salicyl-C_5_), 153.68 (sulfamoylphenyl-C_1_), 163.91(salicyl-C_2_), 168.13 (C = O). Anal. Calcd.for C_20_H_18_N_4_O_4_S (410.45): C, 58.53; H, 4.42; N, 13.65; S, 7.81. Found: C, 58.79; H, 4.55; N, 13.42; S, 7.89. EIMS m/z (% relative abundance): 410.42 (4.8) (M^+•^), 393.47 (10.02), 314.40 (13.69), 167.46 (27.82), 96.14 (32.86), 69.04 (100) (base peak), 53.96 (37.56), 43.98 (43.98).

##### 2.1.1.6. N-Benzyl-2-hydroxy-5-((4-(N-(thiazol-2-yl) sulfamoyl) phenyl) diazenyl) benzamide (5f)

Deep brown crystals; yield 90%, m.p. 281–283 °C. IR (KBr, cm^−1^): 3566 (OH), 3450, 3371 (NH), 1642 (C = O), 1493 (N = N), 1297, 1139 (SO_2_). ^1^H-NMR (400 MHz, DMSO-d6) δ 4.55 (d, *J* = 5.4 Hz, 2H, benzyl-CH_2_), 6.86 (d, *J* = 4.5 Hz, 1H, thiazolyl-C_5_-H), 7.08 (d, *J* = 9.2 Hz, 1H, salicyl-C_3_-H), 7.27 (d, *J* = 4.5 Hz, 2H, benzyl-C_2,6_-H), 7.32 − 7.38 (*m*, 4H, benzyl-C_3,4,5_-H and thiazolyl-C_4_-H), 7.90 − 8.01 (*m*, 5H, sulfamoyl phenyl-C_2,3,5,6_-H and salicyl-C_4_-H), 8.61 (d, *J* = 2.5 Hz, 1H, salicyl-C_6_-H), 9.86 (*t*, J = 5.4 Hz,1H, CH_2_-NH, D_2_O exchangeable),12.06 (*s*, 1H, sulfamoyl NH, D_2_O exchangeable), 13.33 (*s*, 1H, OH, D_2_O exchangeable). Anal. Calcd.for C_23_H_19_N_5_O_4_S_2_ (493.56): C, 55.97; H, 3.88; N, 14.19; S, 12.99. Found: C, 56.09; H, 3.99; N, 14.08; S, 13.11.

##### 2.1.1.7. N-Benzyl-2-hydroxy-5-((4-(N-(pyrimidin-2-yl) sulfamoyl) phenyl) diazenyl) benzamide (5 g)

Deep yellow crystals; yield 93%, m.p. 275–277 °C. IR (KBr, cm^−1^): 3568 (OH), 3393, 3356 (NH), 1648 (C = O), 1493 (N = N), 1337,1145 (SO_2_). ^1^H-NMR (400 MHz, DMSO-d6) δ 4.55 (d, *J* = 5.4 Hz, 2H, benzyl-CH_2_), 7.06 (*t*, *J* = 4.8 Hz, 1H, pyrimidinyl-C_5_-H), 7.13 (d, *J* = 8.9 Hz, 1H, salicyl-C_3_-H), 7.24–7.29 (*m*, 2H, benzyl-C_2,6_-H), 7.33 − 7.39 (*m*, 3H, benzyl-C_3,4,5_-H), 7.98 (d, *J* = 7.8 Hz, 2H, sulfamoyl phenyl-C_2,6_-H), 8.02 (dd, *J* = 8.9, 2.6, 1H, salicyl-C_4_-H), 8.17 (d, *J* = 7.8 Hz, 2H, sulfamoyl phenyl-C_3,5_-H), 8.52 (d, *J* = 4.8 Hz, 2H, pyrimidinyl-C_4,6_-H), 8.63 (d, *J* = 2.6 Hz, 1H, salicyl-C_6_-H), 9.65 (*t*, *J* = 5.4 Hz, 1H, CH_2_-NH, D_2_O exchangeable) 12.06 (*s*, 1H, sulfamoyl NH, D_2_O exchangeable), 13.30 (*s*, 1H, OH, D_2_O exchangeable). ^13 ^C-NMR (126 MHz, DMSO-D6) δ 42.62 (benzyl-CH_2_), 115.94 (pyrimidinyl-C_5_), 118.77(salicyl-C_3_), 122.45(salicyl-C_1_), 126.04 (sulfamoylphenyl-C_2,6_), 126.57 (salicyl-C_4_), 127.05 (salicyl-C_6_), 127.44 (benzyl-C_2,6_), 128.44 (benzyl-C_4_), 129.06 (benzyl-C_3,5_), 132.94 (sulfamoylphenyl-C_3,5_), 138.74 (sulfamoylphenyl-C_4_), 141.74 (benzyl-C_1_), 144.59 (salicyl-C_5_), 154.05 (sulfamoylphenyl-C_1_), 156.76 (pyrimidinyl-C_4,6_), 158.50 (pyrimidinyl-C_2_), 163.62 (salicyl-C_2_), 168.09 (C = O). Anal. Calcd. for C_24_H_20_N_6_O_4_S (488.52): C, 59.01; H, 4.13; N, 17.20; S, 6.56. Found: C, 59.13; H, 4.24; N, 17.09; S, 6.64.

##### 2.1.1.8. 4-((4-Hydroxy-3-(piperidine-1-carbonyl) phenyl) diazenyl) benzenesulfonamide (5 h)

Yellow crystals; yield 95%, m.p. 220–222 °C. IR (KBr, cm^−1^): 3568 (OH), 3384, 3346 (NH_2_), 1681 (C = O), 1482 (N = N), 1349, 1148 (SO_2_). ^1^H-NMR (400 MHz, DMSO-d6) δ 1.41–1.61 (*m*, 6H, piperidinyl-C_3,4,5_-H), 3.14–3.25, 3.51–3.65 (2 m, 4H, piperidinyl-C_2,6_-H), 7.08 (d, *J* = 8.7 Hz, 1H, salicyl-C_3_-H), 7.52 (*s*, 2H, sulfamoyl NH_2_, D_2_O exchangeable), 7.71 (d, *J* = 2.5 Hz, 1H, salicyl-C_6_-H), 7.89 (dd, *J* = 8.7, 2.5 Hz, 1H, salicyl-C_3_-H), 7.95 − 8.01 (*m*, 4H, sulfamoyl phenyl-C_2,3,5,6_-H), 10.87 (*s*, 1H, OH, D_2_O exchangeable). Anal. Calcd. for C_18_H_20_N_4_O_4_S (388.44): C, 55.66; H, 5.19; N, 14.42; S, 8.25. Found: C, 55.78; H, 5.29; N, 14.30; S, 8.36.

##### 2.1.1.9. 4-((4-Hydroxy-3-(piperidine-1-carbonyl) phenyl) diazenyl)-N-(thiazol-2-yl) benzenesulfonamide (5i)

Orang crystals; yield 90%, m.p. 164–166 °C. IR (KBr, cm^−1^): 3651 (OH), 3451 (NH), 1574 (C = O), 1481 (N = N), 1328, 1138 (SO_2_). ^1^H-NMR (400 MHz, DMSO-d6) δ 1.42–1.61 (*m*, 6H, piperidinyl-C_3,4,5_-H), 3.14–3.25, 3.51–3.65 (2 m, 4H, piperidinyl-C_2,6_-H), 6.87 (d, *J* = 4.5 Hz, 1H, thiazolyl-C_5_-H), 7.07 (d, *J* = 8.7 Hz, 1H, salicyl-C_3_-H), 7.28 (d, *J* = 4.5 Hz, 1H, thiazolyl-C_4_-H), 7.69 (d, *J* = 2.4 Hz, 1H, salicyl-C_6_-H), 7.87 (dd, *J* = 8.7, 2.4 Hz, 1H, salicyl-C_4_-H), 7.91–7.99 (m, 4H, sulfamoyl phenyl-C_2,3,5,6_-H), 10.87 (*s*, 1H, sulfamoyl NH, D_2_O exchangeable), 12.86 (*s*, 1H, OH, D_2_O exchangeable) 0.13^ ^C-NMR(126 MHz, DMSO-D6) δ 24.05 (piperidinyl-C_4_), 25.63 (piperidinyl-C_3,5_), 47.34 (piperidinyl-C_2,6_), 108.60 (thiazolyl-C_5_), 116.41(salicyl-C_3_), 119.31(salicyl-C_1_), 122.53(sulfamoylphenyl-C_2,6_), 124.63(salicyl-C_4_), 125.66(salicyl-C_6_), 126.17 (thiazolyl-C_4_), 127.19 (sulfamoylphenyl-C_3,5_), 143.51(sulfamoylphenyl-C_4_), 144.93 (salicyl-C_5_), 153.69 (sulfamoylphenyl-C_1_), 157.39 (salicyl-C_2_),165.61(C = O),169.07(thiazolyl-C_2_). Anal. Calcd.for C_21_H_21_N_5_O_4_S_2_ (471.55): C, 53.49; H, 4.49; N, 14.85; S, 13.60. Found: C, 53.61; H, 4.59; N, 14.73; S, 13.68.

##### 2.1.1.10. 4-((4-Hydroxy-3-(piperidine-1-carbonyl) phenyl) diazenyl)-N-(pyrimidin-2-yl) benzenesulfonamide (5j)

Orang crystals; yield 93%, m.p. 267–269 °C. IR (KBr, cm^−1^): 3651 (OH), 3448 (NH), 1581 (C = O), 1498 (N = N), 1349, 1166 (SO_2_). ^1^H-NMR (400 MHz, DMSO-d6) δ 1.41–1.60 (*m*, 6H, piperidinyl-C_3,4,5_-H), 3.14–3.24, 3.52–3.66 (2 m, 4H, piperidinyl-C_2,6_-H), 7.04–7.08 (*m*, 2H, pyrimidinyl-C_5_-H, salicyl-C_3_-H), 7.70 (d, *J* = 2.2 Hz, 1H, salicyl-C_6_-H), 7.87 (dd, *J* = 8.5, 2.2 Hz, 1H, salicyl-C_4_-H), 7.95 (d, *J* = 8.5 Hz, 2H, sulfamoyl phenyl-C_2,6_-H), 8.14 (d, *J* = 8.5 Hz, 2H, sulfamoyl phenyl-C_3,5_-H), 8.51 (d, *J* = 4.8 Hz, 2H, pyrimidinyl-C_4,6_-H), 10.90 (s, 1H, sulfamoyl NH, D_2_O exchangeable), 12.02 (br.s, 1H, OH, D_2_O exchangeable). Anal. Calcd.for C_22_H_22_N_6_O_4_S (466.52): C, 56.64; H, 4.75; N, 18.01; S, 6.87. Found: C, 56.68; H, 4.87; N, 17.89; S, 6.88.

##### 2.1.1.11. 4-((4-Hydroxy-3-(piperidine-1-carbonyl) phenyl) diazenyl)-N-(4-methylpyrimidin-2-yl) benzenesulfonamide (5k)

Orang crystals; yield 97%, m.p. 208–210 °C. IR (KBr, cm^−1^): 3652 (OH), 3567, 3448 (NH), 1612 (C = O), 1472 (N = N), 1337, 1165 (SO_2_). ^1^H-NMR (400 MHz, DMSO-d6) δ 1.41–1.62 (*m*, 6H, piperidinyl-C_3,4,5_-H), 2.32 (*s*, 3H, pyrimidinyl-4-CH_3_), 3.15–3.25, 3.52–3.66 (2 m, 4H, piperidinyl-C_2,6_-H), 6.90 (d, *J* = 5.3 Hz, 1H, pyrimidinyl-C_5_-H), 7.19 (d, *J* = 8.8 Hz, 1H, salicyl-C_3_-H), 7.74 (d, *J* = 2.4 Hz, 1H, salicyl-C_6_-H), 7.88 (dd, *J* = 8.8, 2.4 Hz, 1H, salicyl-C_4_-H), 7.94 (d, *J* = 8.6 Hz, 2H, sulfamoyl phenyl-C_2,6_-H), 8.14 (d, *J* = 8.6 Hz, 2H, sulfamoyl phenyl-C_3,5_-H), 8.31 (d, *J* = 5.3 Hz, 1H, pyrimidinyl-C_6_-H), 10.89 (s, 1H, sulfamoyl NH, D_2_O exchangeable), 11.99 (br.s, 1H, OH, D_2_O exchangeable) 0.13^ ^C-NMR (125 MHz, DMSO-D_6_) δ 23.22 (pyrimidinyl-4-CH_3_-C), 24.07 (piperidinyl-C_4_), 25.69 (piperidinyl-C_3,5_), 47.25 (piperidinyl-C_2,6_) 112.45 (pyrimidinyl-C_5_), 116.44 (salicyl-C_3_), 119.36 (salicyl-C_1_), 122.31(sulfamoylphenyl-C_2,6_), 125.62 (salicyl-C_4_), 126.24 (salicyl-C_6_), 129.25 (sulfamoylphenyl-C_3,5_), 137.59 (sulfamoylphenyl-C_4_), 145.00 (salicyl-C_5_), 153.93(sulfamoylphenyl-C_1_), 156.42 (pyrimidinyl-C_6_), 157.51(pyrimidinyl-C_2_), 159.49 (salicyl-C_2_), 165.62 (pyrimidinyl-C_4_), 168.54 (C = O). Anal. Calcd.for C_22_H_22_N_6_O_5_S (482.52): C, 54.76; H, 4.60; N, 17.42; S, 6.64. Found: C, 54.84; H, 4.64; N, 17.34; S, 6.72. EIMS m/z (% relative abundance): 482.49 (11.78) (M^+•^), 458.70 (48.60), 395.30 (34.33), 360.25 (41.93), 349.02 (50.06), 308.18 (51.43), 282.03 (100) (base peak), 193.02 (35.65).

##### 2.1.1.12. 4-((4-Hydroxy-3-(morpholine-4-carbonyl) phenyl) diazenyl) benzenesulfonamide (5 l)

Pale brown crystals; yield 93%, m.p. 272–274 °C. IR (KBr, cm^−1^): 3450 (OH), 3340 (NH_2_), 1610 (C = O), 1489 (N = N), 1345, 1154 (SO_2_). ^1^H-NMR (400 MHz, DMSO-d6) δ 3.53–3.69 (*m*, 8H, morpholinyl-H), 7.11 (d, *J* = 8.8 Hz, 1H, salicyl-C_3_-H), 7.50 (*s*, 2H, sulfamoyl NH_2_, D_2_O exchangeable), 7.77 (d, *J* = 2.4 Hz, 1H, salicyl-C_6_-H), 7.91 (dd, *J* = 8.8, 2.4 Hz, 1H, salicyl-C_4_-H), 7.96 − 8.01 (*m*, 4H, sulfamoyl phenyl-C_2,3,5,6_-H), 11.00 (*s*, 1H, OH, D_2_O exchangeable). Anal. Calcd.for C_17_H_18_N_4_O_5_S (390.41): C, 52.30; H, 4.65; N, 14.35; S, 8.21. Found: C, 52.41; H, 4.77; N, 14.25; S, 8.29.

##### 2.1.1.13. 4-((4-Hydroxy-3-(morpholine-4-carbonyl)phenyl)diazenyl)-N-(thiazol-2-yl)benzenesulfonamide (5 m)

Brown crystals; yield 95%, m.p. 235–237 °C. IR (KBr, cm^−1^): 3653 (OH), 3454 (NH), 1603 (C = O), 1483 (N = N), 1411, 1141 (SO_2_). ^1^H-NMR (400 MHz, DMSO-d6) δ 3.52–3.69 (*m*, 8H, morpholinyl-H), 6.86 (d, *J* = 4.6 Hz, 1H, thiazolyl-C_5_-H), 7.14 (d, *J* = 8.9 Hz, 1H, salicyl-C_3_-H), 7.27 (d, *J* = 4.6 Hz, 1H, thiazolyl-C_4_-H), 7.74 (d, *J* = 2.5 Hz, 1H, salicyl-C_6_-H), 7.88 (dd, *J* = 8.9, 2.5 Hz, 1H, salicyl-C_4_-H), 7.92–7.98 (m, 4H, sulfamoyl phenyl-C_2,3,5,6_-H), 11.06 (s, 1H, sulfamoyl NH, D_2_O exchangeable), 12.87 (br.s, 1H, OH, D_2_O exchangeable) 0.13^ ^C-NMR (125 MHz, DMSO-D_6_) δ 46.62 (morpholinyl-C_3,5_), 66.50 (morpholinyl-C_2,6_), 108.62 (thiazolyl-C_5_), 116.52 (salicyl-C_3_), 119.32 (salicyl-C_1_), 120.87 (sulfamoylphenyl-C_2,6_), 124.91(salicyl-C_4_), 125.16 (salicyl-C_6_), 125.55 (thiazolyl-C_4_), 129.02(sulfamoylphenyl-C_3,5_), 137.16 (sulfamoylphenyl-C_4_), 145.96 (salicyl-C_5_), 153.08 (sulfamoylphenyl-C_1_), 159.79 (salicyl-C2), 168.64 (C = O), 169.21(thiazolyl-C_2_). Anal. Calcd.for C_20_H_19_N_5_O_5_S_2_ (473.52): C, 50.73; H, 4.04; N, 14.79; S, 13.54. Found: C, 50.85; H, 4.12; N, 14.68; S, 13.58. EIMS m/z (% relative abundance): 476.32 (3.62) (M^+•^+3), 473.46 (13.41) (M^+•^), 401.27 (19.48), 393.28 (38.19), 371.15 (17.19), 157.01 (15.13), 125.34 (20.48), 84.94 (24.86), 70.99 (34.08), 44.41 (100) (base peak).

##### 2.1.1.14. 4-((4-Hydroxy-3-(morpholine-4-carbonyl) phenyl) diazenyl)-N-(pyrimidin-2-yl) benzenesulfonamide (5n)

Shiny brown crystals; yield 95%, m.p. 241–243 °C. IR (KBr, cm^−1^): 3651 (OH), 3450 (NH), 1583 (C = O), 1500 (N = N), 1335, 1162 (SO_2_). ^1^H-NMR (400 MHz, DMSO-d6) δ 3.51–3.67 (*m*, 8H, morpholinyl-H), 7.05 (*t*, *J* = 4.9 Hz, 1H, pyrimidinyl-C_5_-H), 7.11 (d, *J* = 8.8 Hz, 1H, salicyl-C_3_-H), 7.75 (d, *J* = 2.5 Hz, 1H, salicyl-C_6_-H), 7.89 (dd, *J* = 8.8, 2.5 Hz, 1H, salicyl-C_4_-H), 7.95 (d, *J* = 8.6 Hz, 2H, sulfamoyl phenyl-C_2,6_-H), 8.15 (d, *J* = 8.6 Hz, 2H, sulfamoyl phenyl-C_3,5_-H), 8.51 (d, *J* = 4.9 Hz, 2H, pyrimidinyl-C_4,6_-H), 11.03 (s, 1H, sulfamoyl NH, D_2_O exchangeable), 11.97 (*s*, 1H, OH, D_2_O exchangeable). ^13 ^C-NMR (125 MHz, DMSO-D6) δ 46.91(morpholinyl-C_3,5_), 66.35 (morpholinyl-C_2, 6_), 116.57(pyrimidinyl-C_5_), 119.22(salicyl-C_3_), 122.49(salicyl-C_1_), 123.19(sulfamoylphenyl-C_2,6_), 124.78(salicyl-C_4_), 126.50(salicyl-C_6_), 129.05(sulfamoylphenyl-C_3,5_), 132.15(sulfamoylphenyl-C_4_), 145.02(salicyl-C_5_), 154.13(sulfamoylphenyl-C_1_), 156.80(pyrimidinyl-C_4,6_), 157.54(pyrimidinyl-C_2_), 158.39 (salicyl-C_2_), 165.98 (C = O). Anal. Calcd.for C_21_H_20_N_6_O_5_S (468.49): C, 53.84; H, 4.30; N, 17.94; S, 6.84. Found: C, 53.92; H, 4.34; N, 17.83; S, 6.92.

##### 2.1.1.15. 4-((4-Hydroxy-3-(morpholine-4-carbonyl) phenyl) diazenyl)-N-(4-methylpyrimidin-2-yl) benzenesulfonamide (5o)

Shiny brown crystals; yield 95%, m.p. 205–207 °C. IR (KBr, cm^−1^): 3652 (OH), 3450 (NH), 1612 (C = O), 1472 (N = N), 1337, 1165 (SO_2_). ^1^H-NMR (400 MHz, DMSO-d6) δ 2.32 (*s*, 3H, pyrimidinyl-4-CH_3_), 3.51–3.66 (m, 8H, morpholinyl-H), 6.90 (d, *J* = 5.3 Hz, 1H, pyrimidinyl-C_5_-H), 7.19 (d, *J* = 8.8 Hz, 1H, salicyl-C_3_-H), 7.74 (d, *J* = 2.4 Hz, 1H, salicyl-C_6_-H), 7.88 (dd, *J* = 8.8, 2.4 Hz, 1H, salicyl-C_4_-H), 7.94 (d, *J* = 8.6 Hz, 2H, sulfamoyl phenyl-C_2,6_-H), 8.14 (d, *J* = 8.6 Hz, 2H, sulfamoyl phenyl-C_3,5_-H), 8.31 (d, *J* = 5.3 Hz, 1H, pyrimidinyl-C_6_-H), 11.21 (s, 1H, sulfamoyl NH, D_2_O exchangeable), 11.85 (*s*, 1H, OH, D_2_O exchangeable). Anal. Calcd.for C_22_H_22_N_6_O_5_S (482.52): C, 54.76; H, 4.60; N, 17.42; S, 6.64. Found: C, 54.84; H, 4.71; N, 17.32; S, 6.68.

### 2.2. Biological evaluation

#### 2.2.1. *In vitro* COX-1 and COX-2 enzymatic assay

All the synthesised target compounds were screened for their COX-1 and COX-2 inhibitory activities according to the previously reported methods^49^^,^^50^ (page S34, supplementary file).

#### 2.2.2. Carrageenan-induced paw edoema in mice

According to the reported procedures for carrageenan-induced paw edoema test in mice^51^, this test was carried out for compounds **5 b, 5j, 5n** and **5o**. (Approved by HU-IACUC)^52^ (Pages S34 and S35, supplementary file).

#### 2.2.3. Determination of ED_50_

ED_50_ of compounds **5 b, 5j, 5n** and **5o** was calculated as reported^53^. (Page S35, supplementary file).

#### 2.2.4. Estimation of rat plasma PGE2

Plasma PGE2 concentration of compounds **5 b, 5j, 5n** and **5o** was also measured as previously reported^54^. (Approved by HU-IACUC)^52^ (Page S35, supplementary file).

#### 2.2.5. Gastric ulcerogenic activity

The acute gastric ulcerogenic effect of compounds **5 b, 5j, 5n** and **5o** in adult male Wistar rats was evaluated as previously reported^55^. (Approved by HU-IACUC)^52^ (Pages S35 and S36, supplementary file).

#### 2.2.6. Antibacterial screening

##### 2.2.6.1. Inhibition-zone measurements

Inhibition zones of the target compounds was calculated as previously reported^56^. (Page S36, supplementary file).

##### 2.2.6.2. Minimal inhibitory concentration (MIC) measurement

MIC of test compounds was measured according to the reported methods^57^. (Page S36, supplementary file).

##### 2.2.6.3. Minimal bactericidal concentration (MBC) measurement

MBC tests were also carried out as reported^58^. (Page S37, supplementary file).

#### 2.2.7. *In vivo* antibacterial screening in mice (bacteremic infection)

The *in vivo* antibacterial activity of compounds **5 b, 5j, 5n** and **5o** against *E.coli* and *Staphylococcus aureus* using sulfasalazine as a reference drug^59^ was performed as reported^60^. (Approved by HU-IACUC)^52^ (Pages S37 and S38, supplementary file).

### 2.3. Docking studies

Molecular Operating Environment (MOE 2018.0802) software^61^^,^^62^ https://www.chemcomp.com/Research-Citing_MOE.htm, Chemical Computing Group (Chemical Computing Group, Quebec, Canada, Montreal, Canada) was utilised in performing Molecular docking studies. Protein preparation was performed as previously reported^63^, page S38, supplementary file.

### 2.4. *In silico* prediction of physicochemical properties, drug likeness score, pharmacokinetics, toxicity profile and ligand efficiency metrics

In the present study, prediction of the physicochemical properties was performed using Molinspiration chemoinformatic server, pharmacokinetics by Pre-ADMET calculator, drug likeness score and toxicological effects by Osiris property explorer, page S38, supplementary file.

## 3. Results and discussion

### 3. 1. Chemistry

**Scheme 1.**
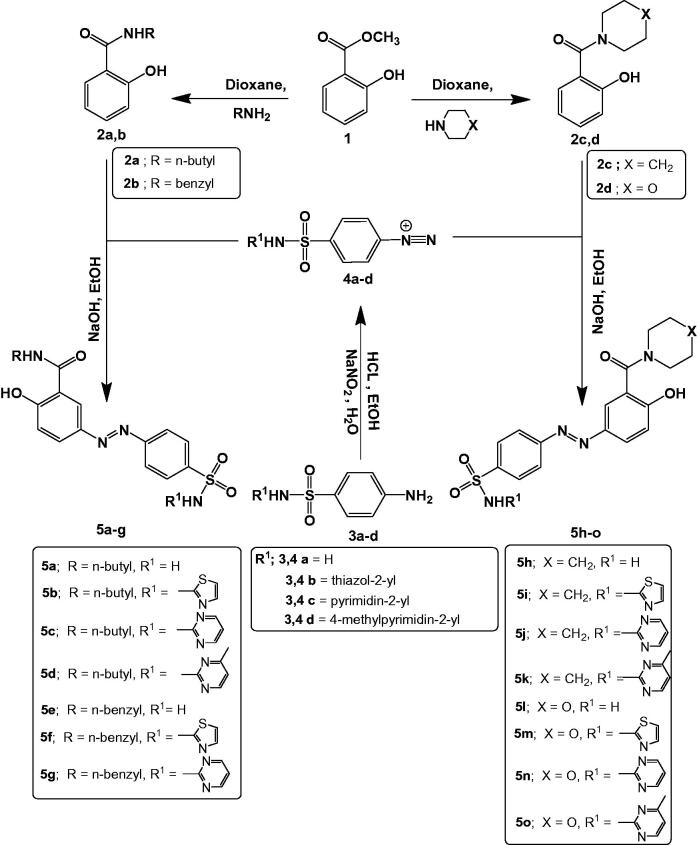
Synthesis of the target co-drugs (**5a-o**).

The synthetic routes for the preparation of the intermediates and target compounds are outlined in Scheme 1. Aminolysis reaction of methyl salicylate **(1)** with the selected primary or secondary amines was employed to obtain N-Substituted-2-hydroxybenzamides **(2a-d)** in good yields and with pure regioselectivity as reported previously^45^^,^^64^. The target azo compounds **(5a-o)** were attained by diazotisation of the appropriate sulphonamide with NaNO_2_ and HCl at 0 °C followed by addition of the appropriate benzamide **(2a-d)**. Moreover, integrity of the structures of the newly synthesised compounds was justified by microanalysis, IR, ^1^H-NMR, ^13 ^C-NMR and MS data (see chemistry experimental section and supporting information). The IR spectra of compounds **5a-o** were characterised by the absorption bands specific for OH, NH, C = O, N = N and SO_2_ functional groups. ^1^H-NMR spectra of compounds **5a-d** were characterised by appearance of one D_2_O exchangeable triplet signal around δ 9.12 ppm stands for n-butyl NH proton in addition to two D_2_O exchangeable singlets stands for sulfamoyl NH proton in the range δ 11.96 − 12.84 ppm for **5 b, 5c** and **5d** and sulfamoyl NH_2_ protons at δ 7.51 ppm for **5a** together with OH proton around δ 13.52 ppm. Moreover, compounds **5e-g** were distinguished by appearance of one signal around δ 4.55 ppm corresponding to benzyl CH_2_ protons, three D_2_O exchangeable signals; in the range δ 9.65 − 9.9.86 ppm corresponding to n-benzyl NH proton, around δ 12.06 ppm corresponding to sulfamoyl NH proton for **5f** and **5 g**, around δ 7.53 ppm corresponding to sulfamoyl NH_2_ protons for **5e** and around δ 13.30 ppm corresponding to OH proton. Besides, compounds **5 h-k** were differentiated by three multiplets in the ranges δ 1.41–1.62, 3.14–3.25 and 3.51–3.66 ppm corresponding to the ten piperidinyl protons, in addition to one D_2_O exchangeable singlet around δ 10.90 ppm corresponding to sulfamoyl NH proton for **5i, 5j** and **5k** and around δ 7.52 ppm corresponding to sulfamoyl NH_2_ protons for **5 h** together with one D_2_O exchangeable singlet in the range δ 10.87–12.86 ppm corresponding to OH proton. Furthermore, compounds **5 l-o** were distinguished by appearance of multiplet signal in the range δ 3.51–3.69 ppm stands for the eight morpholinyl protons together with two D_2_O exchangeable singlets; around δ 7.50 ppm corresponding to sulfamoyl NH_2_ protons for **5 l**, in the range δ 11.03–11.21 ppm corresponding to sulfamoyl NH proton for **5 m, 5n** and **5o** and in the range δ 11.00–12.87 ppm corresponding to OH proton. ^13 ^C-NMR spectra proved the appearance of C = O signal at its expected region (δ165.61–168.64 ppm). It also showed the presence of signals assigned to n-butyl moiety around δ 13.72, 19.67, 30.82 and 46.47 ppm for compounds **5a and 5c**, signal assigned to benzyl-CH_2_ around δ 42.65 ppm for compounds **5e** and **5 g**, three signals around δ 24.05, 25.63 and 47.34 ppm corresponding to piperidinyl carbons for compounds **5i** and **5k**, in addition to two signal around δ 46.62 and 66.50 ppm assigned to morpholinyl carbons for compounds **5 m** and **5n**. Finally, MS spectra showed the molecular ion peak (M^+•^) at *m/z* 454.49 for **5c**, at 410.42 for **5e**, at 482.49 for **5k** and at 473.46 for **5 m**.

### 3.2. Biological evaluation

#### 3.2.1. *In vitro* COX-1 and COX-2 enzymatic assay

Compounds **5a-o** were screened for their *in vitro* COX-1/COX-2 inhibitory activities using an ovine COX-1/human recombinant COX-2 assay kit (Catalog no. 560131; Cayman Chemicals Inc. Ann Arbour, MI, USA). Celecoxib and Rofecoxib were used as reference selective COX-2 inhibitors and indomethacin and Diclofenac sodium were used as reference non-selective COX inhibitors. The data recorded in Table 1 revealed that all compounds were more potent COX-2 inhibitors than Diclofenac sodium. Compared to indomethacin, compounds **5 b, 5j, 5n** and **5o** were more potent COX-2 inhibitors while compounds **5c, 5i** and **5 l** were equipotent. Moreover, compounds **5d, 5 g, 5k** and **5 m** were nearly comparable to indomethacin. All compounds exhibited weak inhibition of COX-1 compared to indomethacin. Considering Celecoxib, compound **5j** displayed equipotent COX-2 inhibitory activity while compounds **5 b, 5c, 5i, 5 l, 5n** and **5o** were nearly comparable to Celecoxib. Interestingly, all compounds showed balanced weak COX-1 and more potent COX-2 inhibition with selectivity indices (SI) ranging from 58 to 239. The resulted SI values exceeded those for the non-selective COX inhibitors while being inferior to selective COX-2 inhibitors. This could also be considered advantageous by avoiding the cardiovascular side effects of the highly selective COX-2 inhibitors^65^. Careful inspection of the structures of the tested compounds revealed that compounds **5 b, 5j, 5n** and **5o** were the most potent COX-2 inhibitors in this series. The highest activity was observed with the piperidin-1-yl and sulfadiazine derivative **5j**. Replacement of 4-sulfamoylphenyl moiety in compound **5a** with thiazol-2-yl (**5 b**), pyrimidin-2-yl (**5c**) and 4-methylpyrimidin-2-yl (**5d**) respectively seemed to greatly enhance COX-2 inhibitory activity with higher selectivity indices. As observed with compounds **5e-g**, introduction of benzyl moiety decreased COX-2 inhibitory activity. Moreover, the presence of 4-sulfamoylphenyl moiety in compounds **5e** and **5 h** did not enhance COX-2 inhibitory activity making them the least active compounds in this series. Concerning compounds **5i-5k, 5 l, 5n** and **5o**, introduction of piperidin-1-yl and morpholin-1-yl moieties to methyl salicylate portion of the compound significantly enhanced COX-2 inhibitory activity comparable to that of Celecoxib.

**Table 1.** *In vitro* COX-1 and COX-2 inhibitory IC_50_ values and COX SI values of the target compounds (5a-o).

Compound ID	IC_50_ µM		SI^c^ COX-1/COX-2
	COX-1^a^	COX-2^b^	
**Celecoxib**	14.7 ± 0.06	0.05 ± 0.0003	294
**Rofecoxib**	14.5 ± 0.06	0.03 ± 0.0006	483.3
**Indomethacine**	0.1 ± 0.003	0.08 ± 0.0003	1.25
**Diclofenac sodium**	3.8 ± 0.03	0.84 ± 0.003	4.5
**5a**	9.6 ± 0.06	0.11 ± 0.0003	87.3
**5b**	12.2 ± 0.06	0.07 ± 0.0005	187.7
**5c**	11.2 ± 0.08	0.08 ± 0.0003	145.5
**5d**	10.6 ± 0.08	0.09 ± 0.001	116.5
**5e**	8.3 ± 0.06	0.12 ± 0.003	69.2
**5f**	9.7 ± 0.06	0.11 ± 0.003	88.2
**5g**	10.5 ± 0.08	0.10 ± 0.0003	109.4
**5h**	8.2 ± 0.12	0.14 ± 0.000	58.6
**5i**	11.2 ± 0.11	0.08 ± 0.0008	136.6
**5j**	12.2 ± 0.06	0.05 ± 0.0005	239.2
**5k**	10.3 ± 0.08	0.09 ± 0.0003	111.9
**5l**	10.2 ± 0.08	0.08 ± 0.001	134.2
**5m**	9.9 ± 0.00	0.10 ± 0.0008	102.1
**5n**	10.6 ± 0.08	0.07 ± 0.0005	153.6
**5o**	12.9 ± 0.05	0.06 ± 0.0008	211.5

^a,b^Concentration of the compound that causes 50% inhibition of enzymatic activity of cyclooxygenase 1 and 2 (COX-1 and COX-2), respectively and all values are expressed as Mean ± SEM of triplicate determinations.

**^c^**COX-2 selectivity index: (COX-1 _IC50_/COX-2 _IC50_).

#### 3.2.2. Carrageenan-induced paw oedema in mice

**Figure 2.**
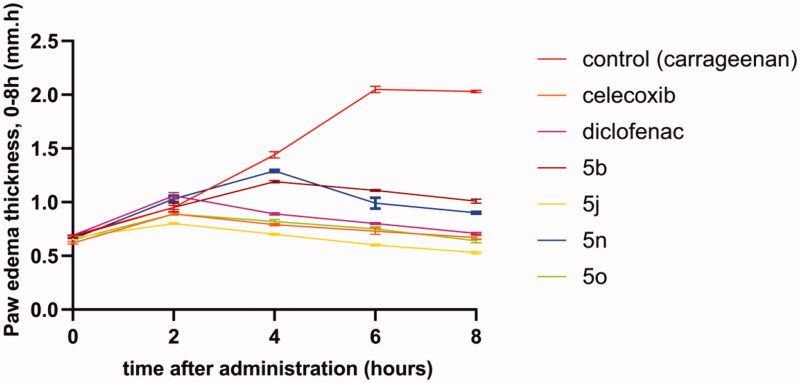
Effects of the target compounds 5 b, 5j, 5n and 5o on the thickness of carrageenan-induced paw edoema in mice along interval of 0 – 8 h after injecting carrageenan (mm) together with the reference drugs celecoxib and Diclofenac in a dose of 10 µmol/kg.

Although most of the newly synthesised compounds exhibited a promising *in vitro* anti-inflammatory activity, it is important to assess their efficacy inside the biological system this can be attributed to their azo co-drug nature. As a result, they must be subjected to azo reductase enzyme, a metabolising enzyme in the colon, liberating two active metabolites; the sulphonamide portion and the 5-amino salicylamide portion being ready for systemic absorption. Consequently, the synthesised compounds, **5 b, 5j, 5n** and **5o** showing the most potent and selective COX-2 inhibitory activity were subjected to *in vivo* carrageenan-induced paw oedema bioassay in mice using celecoxib and Diclofenac as reference drugs. The results illustrated in Table 2 and Figure 2 revealed that, after 2 h, both **5j** and **5o** exhibited similar pharmacokinetic profiles to celecoxib as revealed from their rapid onset of action. Interestingly, after 2 h, compound **5j** showed more potent inhibition of paw edoema than both celecoxib and Diclofenac. To conclude the anti-inflammatory activity of the tested compounds, the anti-inflammatory activity after 8 h was taken as a point for comparison which showed that, compound **5j** showed the best % inhibition of paw oedema (88.50%) being more than that was displayed by both celecoxib (70.87%) and Diclofenac (69.13%). Furthermore, compound **5o** displayed comparable inhibition of paw oedema (72.17%) to that of celecoxib and Diclofenac. Compounds **5 b** and **5n** also showed moderate anti-inflammatory activities (56.09% and 60.87%, respectively). Furthermore, the ED_50_ values (Table 2) showed that all compounds were nearly equipotent (10.94–18.69 µmol/kg). However, compound **5j** was found to be the most potent among this series with ED_50_ = 10.94 µmol/kg exceeding those for celecoxib (13.07 µmol/kg) and Diclofenac (11.46 µmol/kg). In addition, the ED_50_ of compound **5o** (13.27 µmol/kg) was the same as those for celecoxib.

**Table 2.** Effects of the target compounds 5 b, 5j, 5n and 5o on carrageenan-induced paw edoema in mice (mm), their percentage anti-inflammatory activity and their ED_50_ values (µmol/kg) (95% confidence level).

Compound NO.^a^	Thickness of edoema (mm)^b^					ED_50_ ( µmol /kg) (95% confidence level)
	**0h**	**2h**	**4h**	**6h**	**8h**	
**Control (carrageenan)**	0.69 ± 0.004	0.95 ± 0.02	1.44 ± 0.03	2.05 ± 0.03	2.03 ± 0.01	
**Celecoxib**	0.62 ± 0.01	0.89 ± 0.01^c^	0.79 ± 0.01^c^	0.73 ± 0.03^c^	0.67 ± 0.02^c^	13.07 (10.65 − 15.93)
		(6.32%)^d^	(45.14%)	(64.39%)	(70.87%)	
**Diclofenac**	0.69 ± 0.004	1.06 ± 0.03^c^	0.89 ± 0.01^c^	0.80 ± 0.01^c^	0.71 ± 0.01^c^	11.46 (9.98 − 13.16)
		(-11.58%)	(38.19%)	(60.98%)	(69.13%)	
**5 b**	0.68 ± 0.01	0.95 ± 0.04	1.19 ± 0.01	1.11 ± 0.01	1.01 ± 0.02	18.69 (16.41 − 21.10)
		(0%)	(20.83%)	(45.85%)	(56.09%)	
**5j**	0.67 ± 0.02	0.80 ± 0.01^c^	0.70 ± 0.01^c^	0.60 ± 0.01^c^	0.53 ± 0.01^c^	10.94 (9.48 − 12.65)
		(15.79%)	(51.39%)	(70.73%)	(88.50%)	
**5n**	0.66 ± 0.004	1.03 ± 0.03	1.29 ± 0.01	0.99 ± 0.05	0.90 ± 0.01	14.39 (11.69 − 17.53)
		(-8.42%)	(10.42%)	(51.71%)	(60.87%)	
**5o**	0.65 ± 0.004	0.89 ± 0.01^c^	0.82 ± 0.02^c^	0.75 ± 0.02^c^	0.64 ± 0.02^c^	13.27 (11.81 − 14.88)
		(6.32%)	(43.06%)	(63.41%)	(72.17%)	

Data were analysed by one-way ANOVA followed by Tukey’s Karmer *post hoc* test for multiple comparisons.

^a^Dose level for all compounds, po: 10 µmol/kg b.wt.

^b^Values are expressed as Mean ± SEM (number of animals *n* = 5 mice).

^c^Means are significantly different from the control group (*P* < 0.05).

^d^Values between parentheses: (percentage anti-inflammatory activity (AI%).

#### 3.2.3. Estimation of rat plasma PGE2

**Figure 3.**
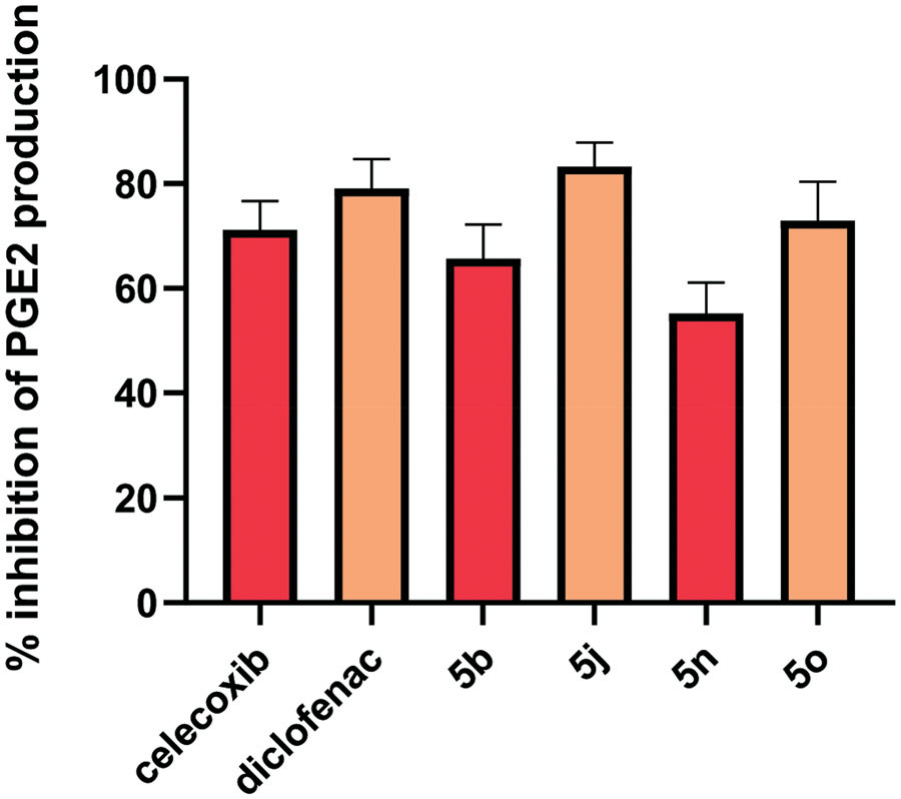
% inhibition of rat serum PGE2 production after 8 h of injecting 10 µmol/kg b.wt of the tested compounds as well as the reference drugs celecoxib and Diclofenac.

Estimating serum levels of PGE2 is a critical parameter to assess the *in vivo* anti-inflammatory efficacies of COX-2 inhibitors. Consequently, compounds **5 b, 5j**, **5n** and **5o** were subjected to estimation of PGE2 in rat serum and results were summarised in Figure 3 (Table 3, page S4 supplementary file). The results showed that, the most active compound was **5j** recording plasma PGE2% inhibition of 83.25% higher than reference celecoxib (% inhibition = 71.18) and Diclofenac (% inhibition = 79.10). Moreover, compound **5o** (72.92%) showed PGE2-diminishing activity comparable to that of celecoxib but slightly lower than that of Diclofenac. Also, **5 b** and **5n** were acceptable PGE2-lowering agents with % PGE2-inhibition of 65.70% and 55.18%, respectively. Finally, the results showed good relevance between selective COX-2 inhibitory activity and decreasing production of plasma PGE2 as one of the prime mediators released through COX-2 enzyme pathway.

#### 3.2.4. Gastric ulcerogenic activity

**Figure 4.**
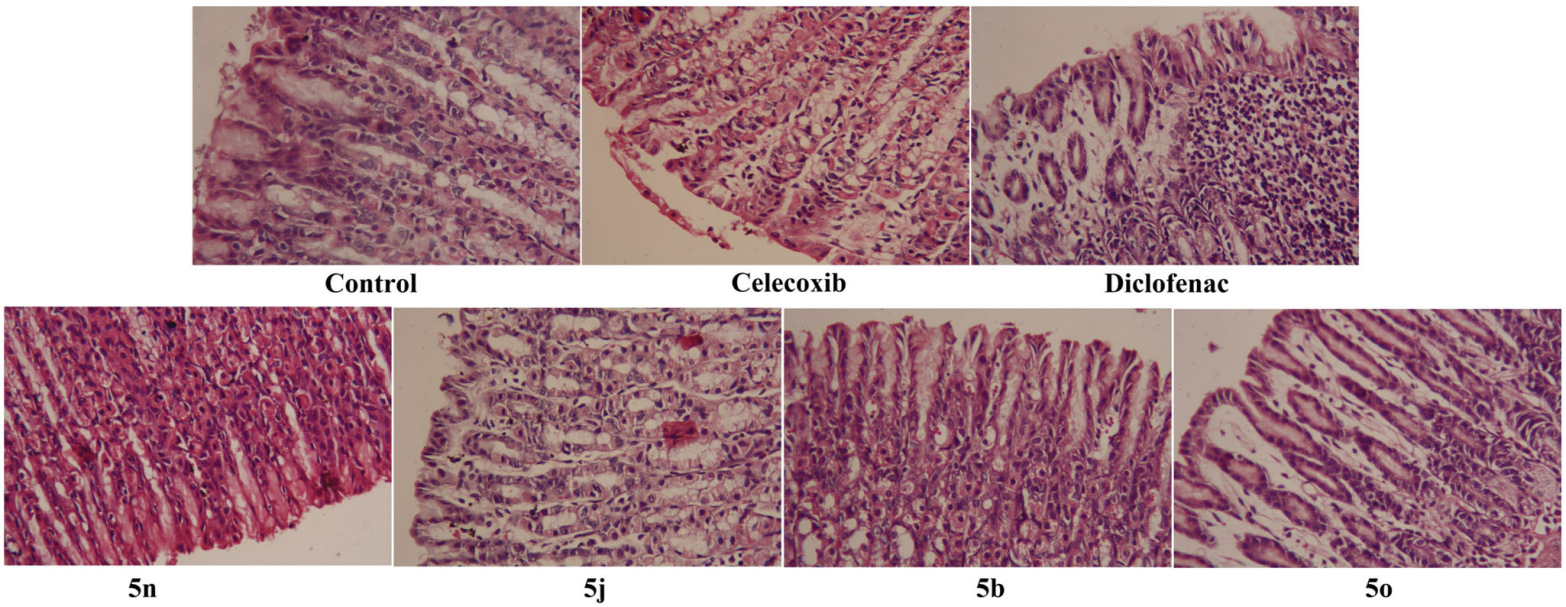
The ulcerogenic effect of the tested compounds 5 b, 5j, 5n and 5o as well as celecoxib and Diclofenac as reference drugs on gastric layers.

Inclusive monitoring of the isolated fasted-rat stomachs revealed a normal stomach texture for compounds **5 b, 5j** and **5n** as well as the reference celecoxib whereas compound **5o** expressed signs of gastric ulcers and hyperaemia as the reference Diclofenac. Furthermore, the degree of inflammatory reactions of the tested compounds in the gastric layers was confirmed by histopathological examination (Figure 4). The results disclosed the gastrointestinal safety profile of compounds **5 b, 5j** and **5n** as well as celecoxib. Nevertheless, the tested compound **5o** exhibited erosion in the gastric layers as the reference Diclofenac.

#### 3.2.5. *In vitro* antibacterial screening

All the newly synthesised compounds were evaluated for their *in vitro* antibacterial activities against the human pathogens: *Staphylococcus aureus (RCMB 0100183)*, *Staphylococcus epidermidis (RCMB 0100183)*, *Streptococcus mutans (RCMB 0100172)*, and *Bacillus subtilis (RCMB 0100162)* as examples of Gram-positive bacteria and *Pseudomonas aeruginosa (RCMB 0100243)*, *Escherichia coli (RCMB 010052)*, *Salmonella typhi (RCMB 0100104)*, *Shigella dysenteriae (RCMB 0100542)* and *Proteus vulgaris (RCMB 010085)* as examples of Gram-negative bacteria using Ampicillin and levofloxacin as standard Gram-positive and Gram-negative antibacterial agents respectively[6] (Table 5, page S5 supplementary file) see experimental section. The results showed that, most of the tested compounds did not exhibit significant *in vitro* antibacterial activity, whereas compounds (**5 b, 5i, 5j, 5k, 5 m, 5n,** and **5o**) displayed weak *in vitro* antibacterial activity this could be assigned to the fact that the investigated compounds were azo co-drugs. Hence, metabolic biotransformation by azo-reductase enzyme into their active metabolites is a requirement for expressing their activities.

#### 3.2.6. *In vivo* antibacterial screening in mice

Compounds **5 b, 5j, 5n** and **5o** as well as **sulfasalazine** as a reference drug were evaluated for their *in vivo* antibacterial efficacy against experimental bacteremic infections caused by *S. aureus* as gram-positive and *E.coli* as gram-negative bacteria in mice and their ED_50_ values were determined, summarised and listed in Table 3. Concerning infection caused by *S. aureus*, all tested compounds improved the survival rate of model mice compared to positive control group which died. Compound **5j** displayed the most potent activity with ED_50_ value of 302.1 µmol/kg being slightly potent *S.aureus* inhibitor than **sulfasalazine** (313.7 µmol/kg). Moreover, compounds **5 b, 5n** and **5o** showed good antibacterial activity with ED_50_ values ranging from 352.7 to 413.1 µmol/kg. By investigating the inhibitory activity against *E.coli*, all the tested compounds displayed antibacterial activity higher than sulfasalazine activity (ED50 = 272.7 − 415.7 µmol/kg vs 419.2 µmol/kg). Compound **5j** was the most potent one against E.coli (ED_50_ = 272.7 µmol/kg). Furthermore, compound **5 b** was found to have equal inhibitory effects against both *S.aureus* and *E.coli* (ED_50_ = 413.1 and 415.7 µmol/kg, respectively).

**Table 3.** The protective effects of promising compounds and the reference drug, sulfasalazine against bacteremic infections in mice ED_50_ values in µmol/kg/day (95% confidence limit).

organism	Challenge dose(CFU/mouse)	drug	ED_50_ (µmol/kg/day)	95% confidence limit
*Staphylococcus aureus*	9.5x10^6^	Sulfasalazine	313.7	(84.62 – 523.8)
		5b	413.1	(318.5 – 511.4)
		5j	302.1	(214.7 – 373.3)
		5n	397.6	(78.40 – 583.3)
		5o	352.7	(315.4 – 398.1)
*Escherichia coli*	7.1x10^3^	sulfasalazine	419.2	(194.5 – 690.8)
		5b	415.7	(275.6 – 534.5)
		5j	272.7	(157.1 – 386.2)
		5n	367.1	(166.9 – 565.7)
		5o	306.4	(160.0 – 445.5)

### 3.3. Docking studies of the potential dual COX-2/DHPS inhibitors

We docked the target azo-dye prodrugs **(5 b)**, **(5n)**, **(5o)**, and **(5j)** into COX-2 active site (pdb entry 3LN1)^66^ which showed potential anti-inflammatory in both the *in vitro* (Table 1) and *in vivo* (Table 2 and Figure 2) testing. We also docked the expected metabolite of **(5j)** into DHPS active site (pdb entry 3TZF)^67^ after exposure to the azo-reductase^68^into the biological system which showed the most potential anti-bacterial activity in the *in vivo* testing (Table 3).

#### 3.3.1. Docking of the compounds (5 b), (5n), (5o), and (5j) as COX-2 inhibitors:

**Figure 5.**
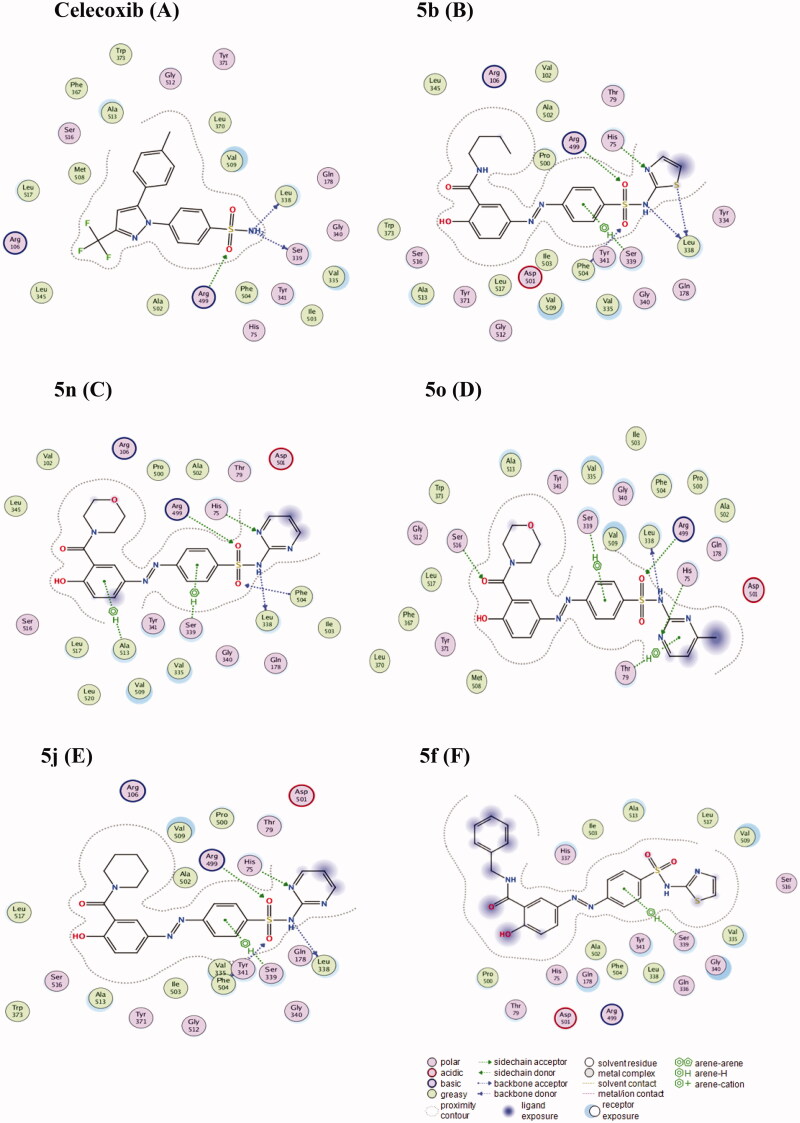
2 D-style of docking solutions of the potential COX-2 inhibitors, **5 b**; **5n**; **5o**; **5j**, and **Celecoxib** as the co-crystallised ligand inhibitor in the active site of COX-2 (PBD code 3LN1). Besides, the docking solution of compound **(5f)** as the least potent inhibitor to visualise its distinct binding pattern with the same active site. Amino acid residues presented by the protein sequence code of three letters and numbers; interaction forces presented by green dotted lines which are categorised according to the description scheme associated with the figure.

The docking solutions of the compounds **(5 b)**, **(5n)**, **(5o)**, and **(5j)** (Figure 5) verified the potential activities against COX-2 as anti-inflammatory agents because the drug-receptor interactions of the best poses are very comparable to that of the co-crystallised ligand inhibitor, celecoxib (Figure 5). All the docked compounds interacted with the Leu 338, Arg499, and Ser 339 amino acids which are redundantly reported^69^ as a part of the polar side pocket of COX-2 active site and interaction with it is required for the selective inhibition of the enzyme. On the other side, we docked compound **(5f)** as one of the compounds of the least potencies against COX-2 (Table 1) to realise the reasons stand behind such low potency. Docking solution of **(5f)** showed failure of the compound to relax freely into the active site in a way to interact with the polar side pocket amino acids except for Ser339. And, it lacks the carboxylic acid group which might have enabled it interact with Arg120 as an alternative path to inhibit the enzyme^69^.

#### 3.3.2. Docking of compound (5j-metabolite) as a potential DHPS inhibitor:

**Figure 6.**
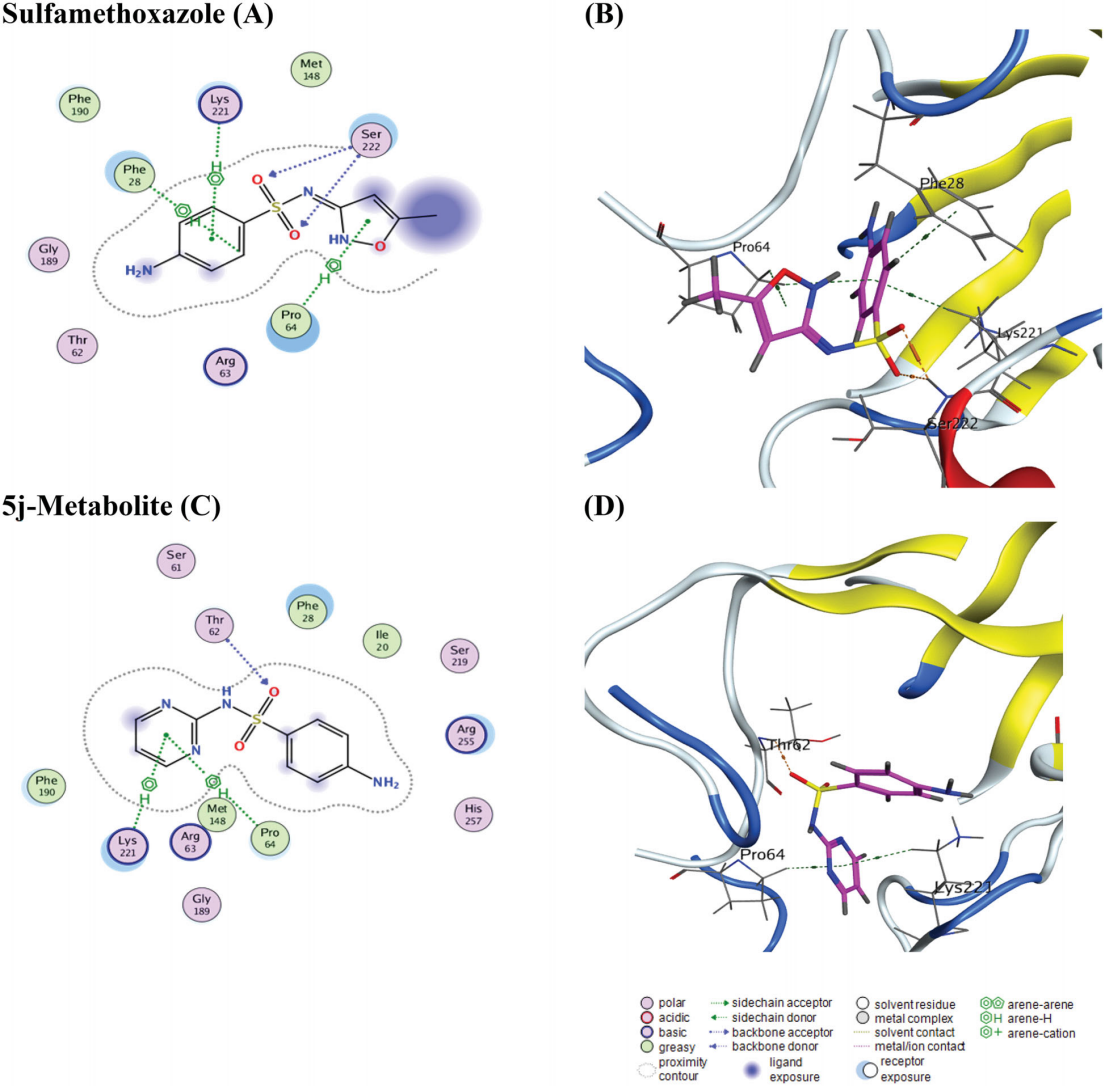
In the left side: 2 D-style of the docking solutions of the co-crystallised ligand inhibitor, **sulfamethoxazole**, and **5j-metabolite** in the active site of DHPS (PBD code 3TZF) to visualise the interaction forces with the active site. In the right side: there are the corresponding 3 D-style docking solutions of **sulfamethoxazole**, and **5j-metabolite** to visualise the orientation of the docked compound in the active site. Amino acid residues presented by the protein sequence code of three letters and numbers, atoms assigned by colours, (blue nitrogen; red oxygen; yellow sulphur; grey carbon). Interaction forces in the 2 D-style are categorised according to the description scheme associated with the figure while in the 3 D-style, it appeared as brown dotted lines for the hydrogen bonding and green dotted lines for the hydrophobic interactions.

Docking solution of **(5j-**metabolite**)** revealed that the compound interacted with the receptor active site with acceptable conformity to the co-crystallised ligand inhibitor, Sulfamethoxazole (Figure 6). The metabolite interacted with Pro64, and Lys221but failed to interact with Phe28 and Ser222 amino acids. It is worthy to mention that the metabolite failed to sit into the active site aligned with the sulfamethoxazole but preferred to invert on its horizontal axis in the opposite direction to relax freely. This might be a reason for the failure of the compound to interact with both Phe28, and Ser222 amino acids as shown in (Figure 6).

### 3.4. *In silico* prediction of the physicochemical properties, drug likeness score, pharmacokinetics, toxicity profile and ligand efficiency metrics

Early prediction of the physicochemical and pharmacokinetic properties of new drug candidates is an infrastructure for lead optimisation as well as drug development process^70^. Accordingly, the *in silico* physicochemical characters, drug likeness, pharmacokinetic parameters, toxicity profile and ligand efficiency metrics of the most active compounds **5 b, 5j, 5n** and **5o** were predicted by Molinspiration^71^. Pre-ADMET^72^ and Osiris property explorer^73^ online soft wares (Table 6, pages S7 and 8 supplementary file).

The calculated values predicted that all the tested compounds comply with Lipinski’s rule of 5 and satisfied Veber’s criteria. Moreover, the results predicted good oral bioavailability and an acceptable molecular flexibility. Additionally, their percentages of absorption (%ABS; calculated as %ABS = 109–0.345 × TPSA)^74^, as well as their solubility satisfied the solubility requirement (>0.0001 mg/L)^75^^,^^76^. Furthermore, all the compounds expected to have low cell permeability in both Caco-2 and MDCK cell models but with low CNS penetration. Meanwhile, all of them were predicted to be excellently absorbed and strongly bound to plasma proteins (>90%)^76^^,^^77^. In another context, the values of LE and LLE for the most active compounds regarding their COX-2 inhibitory activity were calculated by the reported equations^78^, then compared to celecoxib as a reference drug. The results expected the expediency of these compounds as lead-like candidates (for more details see pages S6, 7 and 8 supplementary file).

## 4. Conclusion

Seeking for damping bacterial resistance together with broadening spectrum of the biological activities there by dominating inflammatory bacterial infections, our study employed pharmacophoric hybridisation strategy by combining the antibacterial features of sulphonamides with the anti-inflammatory and antibacterial features of salicylamides through azo-linkage to design a novel series of biodegradable compounds that may be powerful tools to combat resistant bacteria as well as their consecutive inflammatory diseases. The newly synthesised compounds were challenged *in vitro* for their expected antibacterial and COX-inhibitory activities. Compounds **5 b**, **5j**, **5n** and **5o** had been identified as the most potent COX-2 inhibitors among the series with IC_50_ values ranging from 0.05 µm to 0.07 µm and SI values from 153.6 to 239.2. Moreover, all compounds displayed moderate *in vitro* antibacterial activities with MIC values ranging from 25 − 200 µg/ml which may be attributed to their prodrug nature and they become active only inside the biological system after being exposed to azo reductase enzyme. Consequently, the most active COX-2 inhibitors **5 b**, **5j**, **5n** and **5o** were evaluated for their *in vivo* anti-inflammatory and antibacterial activities. Surprisingly, compounds **5j** and **5o** were found to be the most potent inhibitors of carrageenan induced paw oedema in mice with % inhibition 88.50% and 72.17%, respectively and ED_50_ values of 10.94 µmol/kg and 13.27 µmol/kg, respectively. In addition, investigating rat serum concentration of PGE2 revealed that all of the four compounds are potent PGE2 inhibitors with % inhibition in the range of 55.18% − 83.25% which confirmed their COX-2 inhibitory properties. Moreover, *in vivo* antibacterial activity against *S.aureus and E.coli* infections confirmed their potent antibacterial action with ED_50_ values in the range of 302.1 − 413.1 µmol/kg against *S.aureus* and 272.7 − 419.2 µmol/kg against *E.coli*. Furthermore, docking studies of the same four compounds into COX-2 active site emphasised their anti-inflammatory potential in addition to being harmonised to a great extent with their IC_50_ values and selectivity indices obtained from the *in vitro* assay. Besides, docking of **5j** metabolite into DHPS active site illustrated the inhibitory activity of the new candidates against bacteria. Finally, *in silico* prediction of the pharmacokinetic properties and toxicity profile as well as ligand efficiency metrics furnished extra support for the lead-like character of the target compounds. Compound **5j** achieved the target goal as potent *in vivo* dual COX-2/DHPS inhibitor. In general, the antibacterial characters together with anti-inflammatory properties of the newly synthesised compounds confirmed their ability for further optimisation process as potential antibacterial agents.

## Supplementary Material

Supplemental Material

## Abbreviations

COX-2: cyclooxygenase-2 isozyme
PGE2: prostaglandin E2
DHPS: dihydropteroate synthase enzyme
SLS: *streptococcal cytolysin S*
SLO: *streptococcal cytolysin O*
NSAIDs: non-steroidal antiinflammatory drugs

## References

[1] Park JY, Pillinger MH, Abramson SB. Prostaglandin E2 synthesis and secretion: the role of PGE2 synthases. Clin Immunol 2006;119:1737–40. 10.1016/j.clim.2006.01.016 16540375

[2] Nakayama T, Mutsuga N, Yao L, Tosato G. Prostaglandin E2 promotes degranulation-independent release of MCP-1 from mast cells. J Leukoc Biol 2006;79:95–104. 16275896 10.1189/jlb.0405226

[3] Kalinski P. Regulation of Immune Responses by Prostaglandin E2. J Immunol 2012;188:21–8. 22187483 10.4049/jimmunol.1101029 PMC3249979

[4] Serezani CH, Chung J, Ballinger MN, et al. Prostaglandin E 2 Suppresses Bacterial Killing in Alveolar Macrophages by Inhibiting NADPH Oxidase. Am J Respir Cell Mol Biol 2007;37:562–70. 17585108 10.1165/rcmb.2007-0153OC PMC2048683

[5] Obermajer N, Wong JL, Edwards RP, et al. PGE(2)-driven induction and maintenance of cancer-associated myeloid-derived suppressor cells. Immunol Invest 2012;41:635–57. 23017139 10.3109/08820139.2012.695417

[6] Blaschke U, Beineke A, Klemens J, et al. Induction of Cyclooxygenase 2 by Streptococcus pyogenes Is Mediated by Cytolysins. J Innate Immun 2017;9:587–97. 28813715 10.1159/000479153

[7] Wang Y, Ren B, Zhou X, et al. Growth and adherence of Staphylococcus aureus were enhanced through the PGE2 produced by the activated COX-2/PGE2 pathway of infected oral epithelial cells. PLoS One 2017;12:e0177166–21. 28472126 10.1371/journal.pone.0177166 PMC5417706

[8] Annamanedi M, Kalle AM. Celecoxib sensitizes Staphylococcus aureus to antibiotics in macrophages by modulating SIRT1. PLoS One 2014;9:e99285–9. 24950067 10.1371/journal.pone.0099285 PMC4064976

[9] Kalle AM, Rizvi A. Inhibition of bacterial multidrug resistance by celecoxib, a cyclooxygenase-2 inhibitor. Antimicrob Agents Chemother 2011;55:439–42. 20937780 10.1128/AAC.00735-10 PMC3019655

[10] Varma GYN, Kummari G, Paik P, Kalle AM. Celecoxib potentiates antibiotic uptake by altering membrane potential and permeability in Staphylococcus aureus. J Antimicrob Chemother 2019;74:3462–72. 31586409 10.1093/jac/dkz391

[11] Ahmed EF, El-baky RMA, Ahmed ABF, et al. Antibacterial activity of some non-steroidal anti-inflammatory drugs against bacteria causing urinary tract infection. Am J Infect Dis Microbiol 2017;5:66–73.

[12] Elaine WLC, Ling W, Yee ZY, et al. Synergistic effect of non - steroidal anti - inflammatory drugs (NSAIDs) on antibacterial activity of cefuroxime and chloramphenicol against methicillin - resistant Staphylococcus aureus. Integr Med Res 2010;10: 70–4. 10.1016/j.jgar.2017.03.012 28673701

[13] Altaf M, Ijaz M, Ghaffar A, et al. Antibiotic susceptibility profile and synergistic effect of non-steroidal anti-inflammatory drugs on antibacterial activity of resistant antibiotics (Oxytetracycline and Gentamicin) against methicillin resistant Staphylococcus aureus (MRSA). Microb Pathog 2019;137:103755. 31542423 10.1016/j.micpath.2019.103755

[14] Jeśman C, Młudzik A, Cybulska M. [History of antibiotics and sulphonamides discoveries]. Pol Merkur Lekarski 2011;30:320–2. 21675132

[15] El-attar MAZ, Shaaban OG, Elbayaa RY, et al. Design, synthesis, antibacterial evaluation and molecular docking studies of some new quinoxaline derivatives targeting dihyropteroate synthase enzyme. Bioorg Chem 2018;76:437–48. 29275262 10.1016/j.bioorg.2017.12.017

[16] Bano S, Javed K, Ahmad S, et al. Synthesis and biological evaluation of some new 2-pyrazolines bearing benzene sulfonamide moiety as potential anti-inflammatory and anti-cancer agents. Eur J Med Chem 2011;46:5763–8. 22019186 10.1016/j.ejmech.2011.08.015

[17] Zhang H, He S, Peng Y, et al. European Journal of Medicinal Chemistry Design, synthesis and antimicrobial evaluation of novel benzimidazole-incorporated sulfonamide analogues. Eur J Med Chem 2017;136:165–83. 28494254 10.1016/j.ejmech.2017.04.077

[18] Davison EK, Mcgowan JE, Li FF, et al. Bioorganic & Medicinal Chemistry C-2 derivatized 8-sulfonamidoquinolines as antibacterial compounds. Bioorg Med Chem 2021;29:115837. 33223463 10.1016/j.bmc.2020.115837

[19] Taha M, Irshad M, Imran S, et al. Synthesis of piperazine sulfonamide analogs as diabetic-II inhibitors and their molecular docking study. Eur J Med Chem 2017;141:530–7. 29102178 10.1016/j.ejmech.2017.10.028

[20] Sethi KK, Verma SM, Tanç M, et al. Carbonic anhydrase inhibitors: synthesis and inhibition of the cytosolic mammalian carbonic anhydrase isoforms I, II and VII with benzene sulfonamides incorporating 4,5,6,7-tetrachlorophthalimide moiety. Bioorg Med Chem 2013;21:5168–74. 23867389 10.1016/j.bmc.2013.06.035

[21] Küçükbay FZ, Küçükbay H, Tanc M, Supuran CT. Synthesis and carbonic anhydrase I, II, IV and XII inhibitory properties of N-protected amino acid - sulfonamide conjugates. J Enzyme Inhib Med Chem 2016;31:1476–83. 26899532 10.3109/14756366.2016.1147438

[22] Eldehna WM, Al-Ansary GH, Bua S, et al. Novel indolin-2-one-based sulfonamides as carbonic anhydrase inhibitors: synthesis, in vitro biological evaluation against carbonic anhydrases isoforms I, II, IV and VII and molecular docking studies. Eur J Med Chem 2017;127:521–30. 28109946 10.1016/j.ejmech.2017.01.017

[23] Buğday N, Küçükbay FZ, Küçükbay H, et al. Synthesis of novel dipeptide sulfonamide conjugates with effective carbonic anhydrase I, II, IX, and XII inhibitory properties. Bioorg Chem 2018;81:311–8. 30176570 10.1016/j.bioorg.2018.08.032

[24] Küçükbay H, Buğday N, Küçükbay FZ, et al. Synthesis and carbonic anhydrase inhibitory properties of novel 4-(2-aminoethyl)benzenesulfonamide-dipeptide conjugates. Bioorg Chem 2019;83:414–23. 30419497 10.1016/j.bioorg.2018.11.003

[25] Ghorab MM, Ragab FA, Heiba HI, et al. In vitro anticancer screening and radiosensitizing evaluation of some new quinolines and pyrimido[4,5-b]quinolines bearing a sulfonamide moiety. Eur J Med Chem 2010;45:3677–84. 20684857 10.1016/j.ejmech.2010.05.014

[26] Al-said MS, Ghorab MM, Al-qasoumi SI, et al. European Journal of Medicinal Chemistry Synthesis and in vitro anticancer screening of some novel benzenesulfonamides. Eur J Med Chem 2010;45:3011–8. 20413187 10.1016/j.ejmech.2010.03.030

[27] Liu Y, Chen Y, Zhang HY. Handbook of macrocyclic supramolecular assembly. Singapore: Springer; 2020. doi:10.1007/978-981-13-1744-6

[28] PEPPERCORN MA, Boston M. Sulfasalazine. Pharmacology, clinical use, toxicity, and related new drug development 1984;101:377–86. 10.7326/0003-4819-101-3-377 6147110

[29] Hoult RS. Section 1 mode of action pharmacological and biochemical actions of sulphasalazine. Drugs 1986;32:18–26. 10.2165/00003495-198600321-00005 2877850

[30] Bach MK, Brashler JR, Johnson MA. Inhibition by sulfasalazine of ltc synthetase and of rat liver glutathione s-transferases. Biochem Pharmacol 1985;34:2695–704. 10.1016/0006-2952(85)90570-2 2861822

[31] Stephenson K, Yamaguchi Y, Hoch JA. The mechanism of action of inhibitors of bacterial two-component signal transduction systems. J Biol Chem 2000;275:38900–4. 10.1074/jbc.M006633200 10978341

[32] Hilliard JJ, Goldschmidt RM, Licata L, et al. Multiple mechanisms of action for inhibitors of histidine protein kinases from bacterial two-component systems. Antimicrob Agents Chemother 1999;43:1693–9. 10.1128/aac.43.7.1693 PMC89345 10390224

[33] Dunn JA, Coburn RA, Evans RT, et al. Novel topically active antimicrobial and anti-inflammatory compounds for acne. Dermatologic, Cosmeceutic, Cosmet Dev Ther Nov Approaches 2007;243–9.

[34] El-Nagar MKS, Abdu-Allah HHM, Salem OIA, et al. Novel N-substituted 5-aminosalicylamides as dual inhibitors of cyclooxygenase and 5-lipoxygenase enzymes: synthesis, biological evaluation and docking study. Bioorg Chem 2018;78:80–93. 29550533 10.1016/j.bioorg.2018.02.023

[35] Férriz JM, Vávrová K, Kunc F, et al. Salicylanilide carbamates: antitubercular agents active against multidrug-resistant Mycobacterium tuberculosis strains. Bioorg Med Chem 2010;18:1054–61. 10.1016/j.bmc.2009.12.055 20060303

[36] Imramovsky A, Vinšová J, Férriz JM, et al. New antituberculotics originated from salicylanilides with promising in vitro activity against atypical mycobacterial strains. Bioorganic & Medicinal Chemistry 2009;17:3572–9. 19403314 10.1016/j.bmc.2009.04.008

[37] Imramovsky A, Vinšová J, Férriz JM, et al. Salicylanilide esters of N-protected amino acids as novel antimicrobial agents. Bioorganic & Medicinal Chemistry Letters 2009;19:348–51. 19081718 10.1016/j.bmcl.2008.11.080

[38] Imramovsky A, Pesko M, Kralova K, et al. Investigating spectrum of biological activity of 4- and 5-chloro-2-hydroxy-n-[2-(arylamino)-1-alkyl-2-oxoethyl]benzamides. Molecules 2011;16:2414–30. 10.3390/molecules16032414 PMC6259751 21403599

[39] Deng W, Guo Z, Guo Y, et al. Acryloylamino-salicylanilides as EGFR PTK inhibitors. Bioorg Med Chem Lett 2006;16:469–72. 10.1016/j.bmcl.2005.06.088 16275081

[40] Benesova L, Minarik M, Jancarikova D, et al. Multiplicity of EGFR and KRAS mutations in non-small cell lung cancer (NSCLC) patients treated with tyrosine kinase inhibitors. Anticancer Res 2010;1672:1667–71. 20592359

[41] Bartram DJ, Leathwick DM, Taylor MA, et al. Veterinary Parasitology The role of combination anthelmintic formulations in the sustainable control of sheep nematodes. Vet Parasitol 2012;186:151–8. 22245073 10.1016/j.vetpar.2011.11.030

[42] Bak A, Kos J, Michnova H, et al. Consensus-based pharmacophore mapping for new set of N - (disubstituted-phenyl)-3. Int J Mol Sci 2020;21:6583. 10.3390/ijms21186583 PMC7555178 32916824

[43] Brown ME, Fitzner JN, Stevens T, et al. Salicylanilides: selective inhibitors of interleukin-12p40 production. Bioorg Med Chem 2008;16:8760–4. 10.1016/j.bmc.2008.07.024 18715785

[44] Angom RS, Zhu J, Wu AT, et al. LCC-09, a novel salicylanilide derivative, exerts anti-inflammatory effect in vascular endothelial cells. J Inflamm Res 2021;14:4551–65. 34526801 10.2147/JIR.S305168 PMC8436973

[45] Lima RN, Silva VR, De Santos L, et al. Fast synthesis of amides from ethyl salicylate under microwave radiation in a solvent-free system. RSC Adv 2017;7:56566–74.

[46] Fahmy HHI, Soliman GA. Synthesis of New Salicylamide Derivatives with Evaluation of Their Antiinflammatory. Analgesic and Antipyretic Activities 2001;24:180–9. 10.1007/BF02978253 11440073

[47] Gnanaprakasam B, Milstein D. Synthesis of amides from esters and amines with liberation of H 2 under neutral conditions. J Am Chem Soc 2011;133:1682–5. 10.1021/ja109944n 21247162

[48] Modak A, Dutta U, Kancherla R, Maity S, Bhadra M, Mobin SM, Maiti D. Predictably selective (sp^3^)C-O bond formation through copper catalyzed dehydrogenative coupling: Facile synthesis of dihydro-oxazinone derivatives. Org Lett 2014;16:2602–5. 10.1021/ol500670h 24761793

[49] AlFadly ED, Elzahhar PA, Tramarin A, et al. Tackling neuroinflammation and cholinergic deficit in Alzheimer's disease: multi-target inhibitors of cholinesterases, cyclooxygenase-2 and 15-lipoxygenase. Eur J Med Chem 2019;167:161–86. 30771604 10.1016/j.ejmech.2019.02.012

[50] Rashad AY, Kassab SE, Daabees HG, et al. Febuxostat-based amides and some derived heterocycles targeting xanthine oxidase and COX inhibition. Synthesis, in vitro and in vivo biological evaluation, molecular modeling and in silico ADMET studies. Bioorg Chem 2021;113:104948. 34052736 10.1016/j.bioorg.2021.104948

[51] Alfayomy AM, Abdel-Aziz SA, Marzouk AA, Shaykoon MSA, et al. Design and synthesis of pyrimidine-5-carbonitrile hybrids as COX-2 inhibitors: anti-inflammatory activity, ulcerogenic liability, histopathological and docking studies. Bioorg Chem 2021;108:104555. 33376011 10.1016/j.bioorg.2020.104555

[52] El-Dershaby NH, El-Hawash SA, Kassab SE, et al. 2021. Design, Synthesis and Biological Evaluation of Some Novel Sulfonamide Derivatives as Dual Antiinflammatory and Antibacterial Agents, Institutional Animal Care and Use Committee (HU-IACUC), Faculty of Science, Helwan University, Egypt.: Approval No. HU2021/Z/AE0721-01.

[53] El-Hawash SAM, Badawey ESAM, El-Ashmawey IM. Nonsteroidal antiinflammatory agents-part 2 antiinflammatory, analgesic and antipyretic activity of some substituted 3-pyrazolin-5-ones and 1,2,4,5,6,7-3H-hexahydroindazol-3-ones. Eur J Med Chem 2006;41:155–65. 16375992 10.1016/j.ejmech.2005.09.006

[54] Shabaan MA, Kamal AM, Faggal SI, et al. Synthesis and biological evaluation of pyrazolone analogues as potential anti-inflammatory agents targeting cyclooxygenases and 5-lipoxygenase. Arch Pharm (Weinheim) 2020;353:1900308. 10.1002/ardp.201900308 32031284

[55] Srivastava SK, Nath C, Gupta MB, Vrat S, et al. Protection against gastric ulcer by verapamil. Pharmacol Res 1991;23:81–6. 2047362 10.1016/s1043-6618(05)80109-4

[56] Al-Omary FAM, Abou-Zeid LA, Nagi MN, et al. Non-classical antifolates. Part 2: synthesis, biological evaluation, and molecular modeling study of some new 2,6-substituted-quinazolin-4-ones. Bioorg Med Chem 2010;18:2849–63. 20350811 10.1016/j.bmc.2010.03.019

[57] CLSI CLSI. Methods for Dilution Antimicrobial Susceptibility Tests for Bacteria That Grow Aerobically. Clin Lab Standars Inst 2018;32:18.

[58] Weinstein MP, Patel JB, Burnhman C-A, ZImmer BL. MO7: methods for dilution antimicrobial susceptibility tests for bacteria that grow aerobically standard, approval CDM-A. Wayne, PA: Clinical and Laboratory Standards Institute; 2018.

[59] Gout PW, Buckley AR, Simms CR, Bruchovsky N. Sulfasalazine, a potent suppressor of lymphoma growth by inhibition of the x(c)- cystine transporter: a new action for an old drug. Leukemia 2001;15:1633–40. 10.1038/sj.leu.2402238 11587223

[60] Yamaguchi K, Domon H, Miyazaki S, et al. In vitro and in vivo antibacterial activities of CS-834, a new oral carbapenem. Antimicrob Agents Chemother 1998;42:555–63. 9517932 10.1128/aac.42.3.555 PMC105498

[61] Bussiere DE, Xie L, Srinivas H, et al. Structural basis of indisulam-mediated RBM39 recruitment to DCAF15 E3 ligase complex. Nat Chem Biol 2020;16:15–23. 31819272 10.1038/s41589-019-0411-6

[62] Abdellatif KRA, Abdelall EKA, Lamie PF, et al. New pyrazole derivatives possessing amino/methanesulphonyl pharmacophore with good gastric safety profile: design, synthesis, cyclooxygenase inhibition, anti-inflammatory activity and histopathological studies. Bioorg Chem 2020;95:103540. 31911297 10.1016/j.bioorg.2019.103540

[63] Wang M, Lu J, Wang M, et al. Discovery of SHP2-D26 as a First, Potent, and Effective PROTAC Degrader of SHP2 Protein. J Med Chem 2020;63:7510–28. 32437146 10.1021/acs.jmedchem.0c00471

[64] Basha A, Lipton M, Weinreb SM. T&r&e&-on Letters No. $3, pp 4171 - 4174, 1977. Tetrahedron Lett 1977;18:4171–4.

[65] Patrono C. Cardiovascular effects of nonsteroidal anti-inflammatory drugs. Curr Cardiol Rep 2016;18:25–8. 10.1007/s11886-016-0702-4 26841787

[66] Wang JL, Limburg D, Graneto MJ, et al. The novel benzopyran class of selective cyclooxygenase-2 inhibitors. Part 2: the second clinical candidate having a shorter and favorable human half-life. Bioorg Med Chem Lett 2010;20:7159–63. 20709553 10.1016/j.bmcl.2010.07.054

[67] Yun M-K, Wu Y, Li Z, et al. Catalysis and sulfa drug resistance in dihydropteroate synthase. Science 2012;335:1110–4. 22383850 10.1126/science.1214641 PMC3531234

[68] Misal SA, Gawai KR. Azoreductase: a key player of xenobiotic metabolism. Bioresour Bioprocess 2018;5:17.

[69] Kassab SE, Khedr MA, Ali HI, Abdalla MM. Discovery of new indomethacin-based analogs with potentially selective cyclooxygenase-2 inhibition and observed diminishing to PGE2 activities. Eur J Med Chem 2017;141:306–21. 29031075 10.1016/j.ejmech.2017.09.056

[70] Can NÖ, Osmaniye D, Levent S, et al. Design, synthesis and biological assessment of new thiazolylhydrazine derivatives as selective and reversible hMAO-A inhibitors. Eur J Med Chem 2018;144:68–81. 29248751 10.1016/j.ejmech.2017.12.013

[71] Molinspiation. Available from: https://www.molinspiration.com/ [last accessed 4 Feb 2022].

[72] Pre-ADMET. Available from: https://preadmet.bmdrc.kr/ [last accessed 14 Feb 2022].

[73] Osiris Property Explorer. Available from: https://www.organic-chemistry.org/prog/peo/ [last accessed 16 Feb 2022].

[74] Zhao YH, Abraham MH, Le J, et al. Rate-limited steps of human oral absorption and QSAR studies. Pharm Res 2002;19:1446–57. 12425461 10.1023/a:1020444330011

[75] Ahsan MJ, Govindasamy J, Khalilullah H, et al. POMA analyses as new efficient bioinformatics’ platform to predict and optimise bioactivity of synthesized 3a,4-dihydro-3H-indeno[1,2-c]pyrazole-2-carboxamide/carbothioamide analogues. Bioorg Med Chem Lett 2012;22:7029–35. 23099090 10.1016/j.bmcl.2012.09.108

[76] Hassan NW, Saudi MN, Abdel-Ghany YS, et al. Novel pyrazine based anti-tubercular agents: design, synthesis, biological evaluation and in silico studies. Bioorg Chem 2020;96:103610. 32028062 10.1016/j.bioorg.2020.103610

[77] Elzahhar PA, Abd El Wahab SM, Elagawany M, et al. Expanding the anticancer potential of 1,2,3-triazoles via simultaneously targeting Cyclooxygenase-2, 15-lipoxygenase and tumor-associated carbonic anhydrases. Eur J Med Chem 2020;200:112439. 32485532 10.1016/j.ejmech.2020.112439

[78] Hopkins AL, Keserü GM, Leeson PD, et al. The role of ligand efficiency metrics in drug discovery. Nat Rev Drug Discov 2014;13:105–21. 24481311 10.1038/nrd4163

