# Supplementary material for: Rational design of biodegradable sulphonamide candidates treating septicaemia by synergistic dual inhibition of COX-2/PGE2 axis and DHPS enzyme
Source: J Enzyme Inhib Med Chem. 2022 Jun 16;37(1):1737–51. doi: 10.1080/14756366.2022.2086868 (PMC9225712; doi:10.1080/14756366.2022.2086868)
Supplement: Supplemental Material [file IENZ_A_2086868_SM1619.pdf]

Supporting information

Biological screening data:

Table 1. ***In vitro*** **COX-1 and COX-2 inhibitory IC_{50} values and COX SI values of the target compounds.**

Compound ID	Structure	IC_{50} μM		SI[c] COX-1/COX-2
		COX-1[a]	COX-2[b]	
**Celecoxib**	-	14.7±0.06	0.05±0.0003	294
**Rofecoxib**	-	14.5±0.06	0.03±0.0006	483.3
**Indomethacine**	-	0.1±0.003	0.08±0.0003	1.25
**Diclofenac sodium**	-	3.8±0.03	0.84±0.003	4.5
**5a**	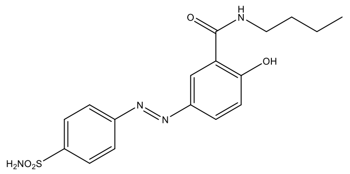	9.6±0.06	0.11±0.0003	87.3
**5b**	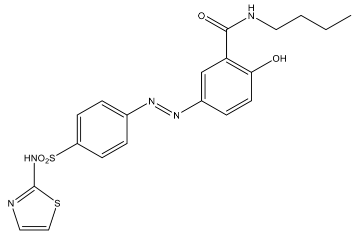	12.2±0.06	0.07±0.0005	187.7
**5c**	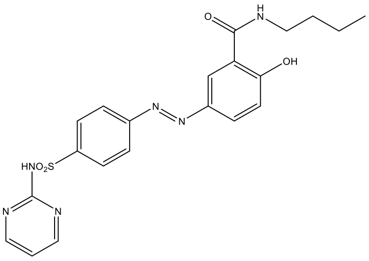	11.2±0.08	0.08±0.0003	145.5
**5d**	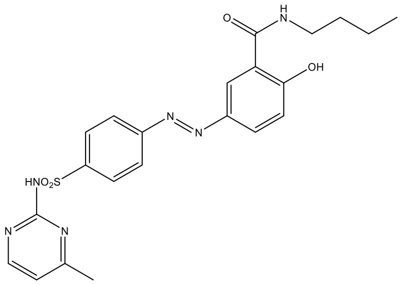	10.6±0.08	0.09±0.001	116.5
**5e**	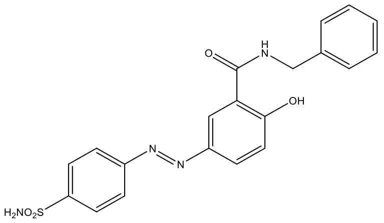	8.3±0.06	0.12±0.003	69.2

**5f**	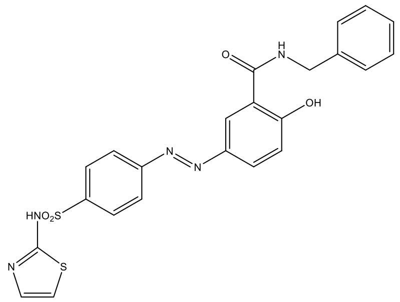	9.7±0.06	0.11±0.003	88.2
**5g**	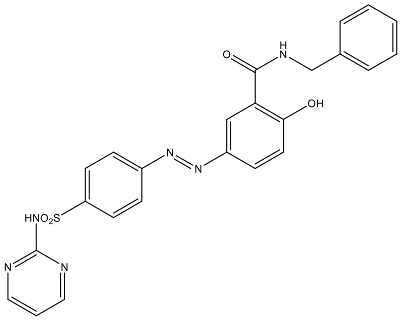	10.5±0.08	0.10±0.0003	109.4
**5h**	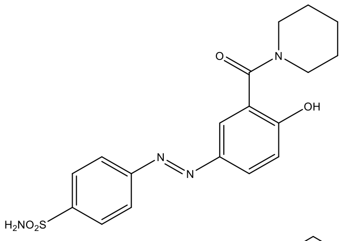	8.2±0.12	0.14±0.000	58.6
**5i**	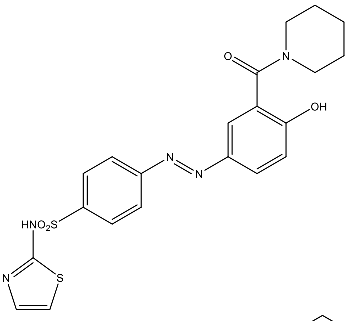	11.2±0.11	0.08±0.0008	136.6
**5j**	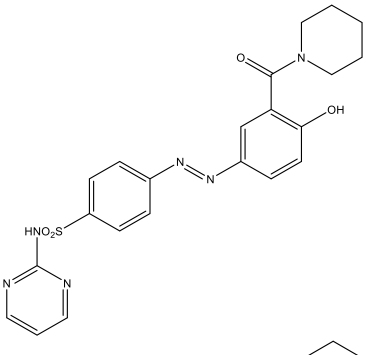	12.2±0.06	0.05±0.0005	239.2
**5k**	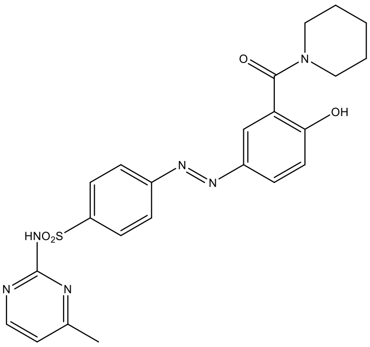	10.3±0.08	0.09±0.0003	111.9

**5l**	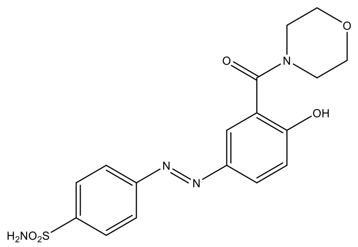	10.2±0.08	0.08±0.001	134.2
**5m**	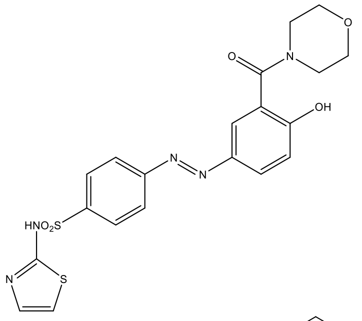	9.9±0.00	0.10±0.0008	102.1
**5n**	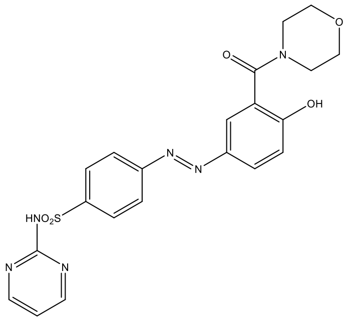	10.6±0.08	0.07±0.0005	153.6
**5o**	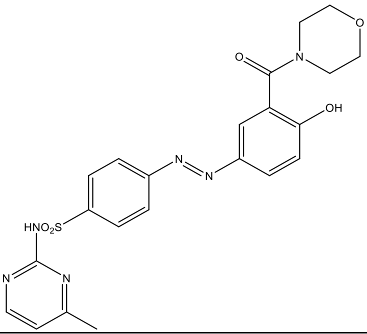	12.9±0.05	0.06±0.0008	211.5

[a,b] Concentration of the compound that causes 50% inhibition of enzymatic activity of cyclooxygenase 1 and 2 (COX-1 and COX-2), respectively.
[a,b] All values are expressed as Mean ± SEM of triplicate determinations.
[c] COX-2 selectivity index: (COX-1 $_{IC50}$ / COX-2 $_{IC50}$).

Table 2. Effects of the target compounds 5b, 5j, 5n and 5o on carrageenan-induced paw edema in mice (mm), their percentage anti-inflammatory activity and their ED_{50} values (µmol/kg) (95% confidence level)

**Compound NO.**[a]	**Thickness of edema (mm)**[b]					**ED_{50} (µmol /kg) (95% confidence level)**
	**0h**	**2h**	**4h**	**6h**	**8h**	
**Control (carrageenan)**	0.69±0.004	0.95±0.02	1.44±0.03	2.05±0.03	2.03±0.01	

**Celecoxib**	0.62±0.01	0.89±0.01[c] (6.32%)[d]	0.79±0.01[c] (45.14%)	0.73±0.03[c] (64.39%)	0.67±0.02[c] (70.87%)	13.07 (10.65 – 15.93)
**Diclofenac**	0.69±0.004	1.06±0.03[c] (-11.58%)	0.89±0.01[c] (38.19%)	0.80±0.01[c] (60.98%)	0.71±0.01[c] (69.13%)	11.46 (9.98 – 13.16)
**5b**	0.68±0.01	0.95±0.04 (0%)	1.19±0.01 (20.83%)	1.11±0.01 (45.85%)	1.01±0.02 (56.09%)	18.69 (16.41 – 21.10)
**5j**	0.67±0.02	0.80±0.01[c] (15.79%)	0.70±0.01[c] (51.39%)	0.60±0.01[c] (70.73%)	0.53±0.01[c] (88.50%)	10.94 (9.48 – 12.65)
**5n**	0.66±0.004	1.03±0.03 (-8.42%)	1.29±0.01 (10.42%)	0.99±0.05 (51.71%)	0.90±0.01 (60.87%)	14.39 (11.69 – 17.53)
**5o**	0.65±0.004	0.89±0.01[c] (6.32%)	0.82±0.02[c] (43.06%)	0.75±0.02[c] (63.41%)	0.64±0.02[c] (72.17%)	13.27 (11.81 – 14.88)

Data were analyzed by one-way ANOVA followed by Tukey's Karmer post hoc test for multiple comparisons.

[a] Dose level for all compounds, po: 10 µmol/kg b.wt.

[b] Values are expressed as Mean ± SEM (number of animals n = 5 mice).

[c] Means are significantly different from the control group ($P < 0.05$).

[d] Values between parentheses: (percentage anti-inflammatory activity (AI%).

Table 3. rat serum PGE2 and % inhibition of PGE2-production of the test compounds

Compound NO.[a]	PGE2 serum conc. (pg/ml)[b]	% inhibition
**Control (pre-carrageenan)**	150.339±5.18[c, d]	_
**Control 1 (post-carrageenan)**	1320.561±7.07[d]	_
**Celecoxib**	380.636±5.53[c, d]	71.18%
**Diclofenac**	276.052±5.60[c, d]	79.10%
**5b**	452.954±6.48[c, d]	65.70%
**5j**	221.236±4.62[c, d]	83.25%
**5n**	591.842±5.96[c, d]	55.18%
**5o**	357.636±7.48[c, d]	72.92%

Data were analyzed by one-way ANOVA followed by Tukey's Karmer post hoc test for multiple comparisons.

[a] Dose level for all compounds, po: 10μmol/kg b.wt.

[b] values are expressed as Mean ± SEM (number of animals n = 5 mice).

[c] Means are significantly different from the control group1 ($P < 0.0001$).

[d] Means are significantly different from each other ($P < 0.0001$).

Table 4. the inhibition zones (IZ) in mm diameter of the target compounds

	Gram positive organisms				Gram negative organisms				
**Compound Number**	***S. aureus***	***S. epidermidis***	***S. mutans***	***B. subtillis***	***P.aeruginosa***	***E.coli***	***S. typhi***	***S. dysenteriae***	***P. vulgaris***
**5a**	26	33	29	17	14	14	15	25	18
**5b**	29	34	27	20	16	14	14	22	19
**5c**	29	30	31	19	16	16	13	25	17
**5d**	28	32	28	20	18	16	16	26	19
**5e**	32	35	30	16	18	16	16	25	21
**5f**	24	36	27	17	16	16	16	25	17
**5g**	26	32	30	20	14	16	13	26	17
**5h**	22	29	29	20	16	14	18	24	19
**5i**	25	30	31	19	18	16	15	22	18
**5j**	28	30	27	19	16	14	15	25	17
**5k**	19	32	28	18	14	16	14	25	19
**5l**	18	32	26	20	14	14	17	26	19
**5m**	25	33	29	20	18	18	16	24	16
**5n**	16	33	28	17	14	17	14	24	18
**5o**	21	35	27	20	18	16	18	23	19
**Ampicillin**	30	32	30	15	_	_	_	_	_
**Levofloxacin**	_	_	_	_	8	11	11	20	15

Table 5. antibacterial minimal inhibitory concentrations (MIC, µmol) and minimal bactericidal concentrations (MBC, µmol) of the target compounds

	Gram positive organisms								Gram negative organisms									
**Compound Number**	***S. aureus***		***S. epidermidis***		***S. mutans***		***B. subtillis***		***P.aeruginosa***		***E.coli***		***S. typhi***		***S. dysenteriae***		***P. vulgaris***	
	**MIC**	**MBC**	**MIC**	**MBC**	**MIC**	**MBC**	**MIC**	**MBC**	**MIC**	**MBC**	**MIC**	**MBC**	**MIC**	**MBC**	**MIC**	**MBC**	**MIC**	**MBC**
**5a**	265.65	265.65	531.31	531.31	66.41	66.41	66.41	132.83	265.65	531.31	265.65	265.65	66.41	132.83	132.83	265.65	265.65	265.65
**5b**	217.61	435.22	108.80	217.61	435.22	435.22	54.40	108.80	108.80	217.61	108.80	217.61	108.80	217.61	108.80	217.61	217.61	217.61
**5c**	55	110.01	110.01	220.02	110.01	165.01	220.02	220.02	220.02	440.03	110.01	220.02	110.01	220.02	330.03	330.03	220.02	220.02
**5d**	213.43	213.43	213.43	320.15	213.43	213.43	213.43	213.43	213.43	213.43	213.43	213.43	213.43	426.87	213.43	213.43	53.36	106.72
**5e**	243.64	243.64	487.27	487.27	304.54	304.54	243.64	243.64	121.82	121.82	243.64	487.27	243.64	487.27	121.82	243.64	121.82	243.65

**5f**	202.61	202.61	101.30	202.61	50.65	50.65	202.61	202.61	202.61	405.22	101.30	101.30	101.30	202.61	202.61	202.61	50.65	50.65
**5g**	51.17	102.35	204.70	204.70	307.05	307.05	102.35	102.35	204.70	204.70	204.70	409.40	102.35	204.70	409.40	409.40	204.70	204.70
**5h**	64.36	128.72	257.44	386.16	257.44	386.16	128.72	128.72	128.72	257.44	257.44	257.44	257.44	514.88	514.88	514.88	128.72	128.72
**5i**	106.03	106.03	212.07	318.1	318.1	318.1	53.02	106.03	212.07	212.07	53.02	106.03	212.07	424.13	212.07	212.07	212.07	424.13
**5j**	53.58	214.35	214.35	428.71	53.58	53.58	107.18	214.35	214.35	428.71	53.58	214.35	214.35	428.71	214.35	214.35	53.58	107.18
**5k**	51.81	103.62	207.25	207.25	414.49	414.49	51.81	207.25	207.25	414-49	103.62	103.62	155.43	207.25	103.62	207.25	103.62	207.25
**5l**	256.14	256.14	256.14	384.21	256.14	256.14	256.14	256.14	256.14	256.14	256.14	256.14	256.14	512.28	256.14	256.14	64.04	128.07
**5m**	52.80	105.59	211.18	211.18	422.37	422.37	52.80	211.18	211.18	422.37	105.59	105.59	158.39	211.18	105.59	211.18	105.59	211.18
**5n**	213.45	213.45	426.90	426.90	53.36	53.36	53.36	106.73	213.45	426.90	213.45	213.45	53.36	106.73	106.73	213.45	213.45	213.45
**5o**	103.62	103.62	207.25	310.87	310.87	310.87	51.81	103.62	207.25	207.25	51.81	103.62	207.25	414.9	207.25	207.25	207.25	414.49
**Ampicillin**	17.89	-	35.78	-	35.78	-	35.78	-		-	-	-		_		_		_
**Levofloxacin**		_		_		_		_	34.59	-	11.07	-	5.53	-	11.07	-	34.59	-

2.4. *In silico* prediction of the physicochemical properties, drug likeness score, pharmacokinetics, toxicity profile and ligand efficiency metrics.

Early prediction of the physicochemical and pharmacokinetic properties of new drug candidates is an infrastructure for lead optimization as well as drug development process[1]. Accordingly, the *in silico* physicochemical characters, drug likeness, pharmacokinetic parameters, toxicity profile and ligand efficiency metrics of the most active compounds **5b, 5j, 5n** and **5o** were predicted by Molinspiration[2]. Pre-ADMET[3] and Osiris property explorer[4] online soft wares (**Table 6**).

The values presented in table 6 showed that all the tested compounds comply with Lipinski's rule of 5, where MW range 459.55-482.52 (≤500), Octanol/water partition coefficient (logP) rang 2.35-5.00 (≤5), HBA range 5-10 (≤10) and HBD range 2-3(≤5). Moreover, all tested compounds complied with Veber's criteria displaying nROTB values of 4–9 (≤10) and only **5n** and **5o** violated

the topographical polar surface area (TPSA; a sum of polar atoms' surfaces: a descriptor for drug absorption, penetrability and bioavailability) showing a value of146.45 Å^2 for each compound (≤140 Å^2). These results indicate good oral bioavailability and an acceptable molecular flexibility. Additionally, the percentage of absorption (%ABS; calculated as %ABS = 109–0.345 × TPSA)[5], as well as the solubility of the compounds were determined. all the tested compounds showed % ABS range 58.47-63.07%, which indicated a considerable bioavailability upon oral administration and their solubility values ranging from 0.16-11.51mg/L, satisfying the solubility requirement (>0.0001 mg/L)[6,7]. The drug-likeness score values showed that all the evaluated compounds gave positive values ranging from 0.05 to 0.07 which were comparable with sulfasalazine (0.07). Furthermore, ADME properties were also predicted using Pre-ADMET software and the results revealed that all the compounds showed low cell permeability in Caco-2 cell model (1.81 – 2.66 nm/s) in relation to the recommended range (4–70 nm/ s). In addition, all of them also displayed low permeability in MDCK cell model (0.28 – 12.53 nm/s) as compared with the acceptable range (25–500 nm/s). Considering the HIA, the tested compounds were excellently absorbed, showing high HIA values (90.86 – 94.98%). Meanwhile, all the evaluated compounds exhibited low CNS penetration as they displayed BBB permeability coefficient in the range of 0.017 – 0.034. Nevertheless, all the tested compounds bound more strongly to plasma proteins (>90%) ranging from 92.28% to 96.85%[7,8]. In another context, to normalize the biological activity of the evaluated compounds with their physicochemical properties, other metrics have been devised. They also measure how the structural features of the molecule affect its binding to the target[9]. Among these metrics: ligand efficiency (LE) and lipophilic ligand efficiency (LLE). LE is the binding energy of the ligand per atom and it evaluates the potency with respect to its molecular size, where the acceptable LE for lead-likeness should be around 0.3 and > 0.3 for drug-likeness. Meanwhile, LEE indicates if lipophilicity is responsible for the specificity of a molecule in binding to the target and the potency. The recommended LLE value should be ≥ 3for lead compound and ≥ 5 for a drug like candidate[10,11]. The values of LE and LLE for the four evaluated compounds regarding their COX-2 inhibitory activity were calculated by the reported equations[11], then compared to celecoxib as a reference drug. The results showed that all the evaluated compounds were concurrent with the LE acceptable value for lead-likeness (around 0.3)with values ranging between (0.29 - 0.30) regarding COX-2 inhibitory action, except for compound 5b whose LE value (0.32) was concordant with acceptable LE value for drug candidates(>0.3). Regarding LLE values of the target compounds, all compounds had LLE values (3.20-4.89) complied with the optimum value for lead-like candidates (≥ 3). Interestingly, in silico predictions confirmed the expediency of these compounds as lead-like candidates.

Table 6. ***In silico*** **prediction of the physicochemical properties, drug likeness, pharmacokinetics and ligand efficiency metrics of compounds 5b, 5j, 5n and 5o**

	5b	5j	5n	5o	sulfasalazine	celecoxib
**Physicochemical parameters and drug-likeness**						
**Log P**[a]	5.00	3.41	2.35	2.79	3.90	3.61
**M.Wt**[b]	459.55	466.52	468.50	482.52	398.40	381.38
**HBA**[c]	9	10	11	11	9	5
**HBD**[d]	3	2	2	2	3	2
**Lipinski's violation**	1	0	1	1	0	0 1
**NROTB**[e]	9	6	6	6	6	4
**TPSA**[f]	133.12	137.22	146.45	146.45	141.32	77.99
**%ABS**[g]	63.07	61.66	58.47	58.47	60.24	82.09
**Volume**[h]	382.72	394.44	386.62	403.18	320.66	298.65
**S**[i]	0.16	3.13	11.51	5.41	1.45	2.08
**Drug-likeness score**[j]	0.05	0.06	0.07	0.07	0.07	0.37
**Pharmacokinetics (ADME)**						
**Caco-2**[k]	1.87	2.25	1.81	2.66	0.38	0.49
**MDCK**[l]	0.28	2.35	12.53	5.15	0.32	45.05
**HIA**[m]	90.86	94.98	92.95	93.66	89.73	96.69
**BBB**[n]	0.021	0.017	0.034	0.029	0.014	0.027
**PPB**[o]	92.28	96.74	96.21	96.85	100	91.08
**Ligand efficiency metrics**						
**LE (COX-2)**[p]	0.32	0.30	0.29	0.29	NT[r]	0.38
**LLE**[q]	3.20	3.81	4.89	4.67	NT	2.87

[a] n-Octanol and water partition coefficient.
[b] Molecular weight.
[c] Number of H-bond acceptors.
[d] Number of H-bond donors.
[e] Lipinski's rule of 5 violations (log P ≤ 5, M.Wt. ≤ 500, HBA ≤ 10 and HBD ≤ 5).
[f] Number of rotatable bonds (Veber's criteria ≤ 10).
[g] Topological polar surface area (Veber's criteria ≤ 140 Å^2).
[h] Percentage of absorption = 109 – (0.345*TPSA).
[i] Molecular volume.
[j] Solubility in mg/L.
[k] Caco-2 permeability (nm/sec): Permeability through cells derived from human colon adenocarcinoma; < 4 nm/sec (low permeability), values from 4 to 70 nm/sec (medium permeability) and > 70 nm/sec (high permeability). Permeability through human colon adenocarcinoma cells.
[l] MDCK permeability (nm/sec): Permeability through Madin–Darby canine kidney cells; < 25 nm/sec (low permeability), values from 25 to 500 nm/sec (medium permeability) and > 500 nm/sec (high permeability). Permeability through Madin-Darby canine kidney cells.
[m] Human intestinal absorption percentage: values from 0 to 20% (poorly absorbed), values from 20 to 70% (moderately absorbed) and values from 70 to 100% (well absorbed).

[n] Blood brain barrier penetration coefficient; values < 0.1 (low CNS penetration), values from 0.1 to 2 (medium CNS absorption) and values > 2 (high CNS absorption).
[o] Plasma protein binding; values < 90% (poorly bound) and > 90% (strongly bound).
[p] Ligand efficiency = (pIC_{50}*1.37) / non-hydrogen atoms.
[q] Lipophilic ligand efficiency = pIC_{50}-logP
[r]NT: not tested.

Experimental data:

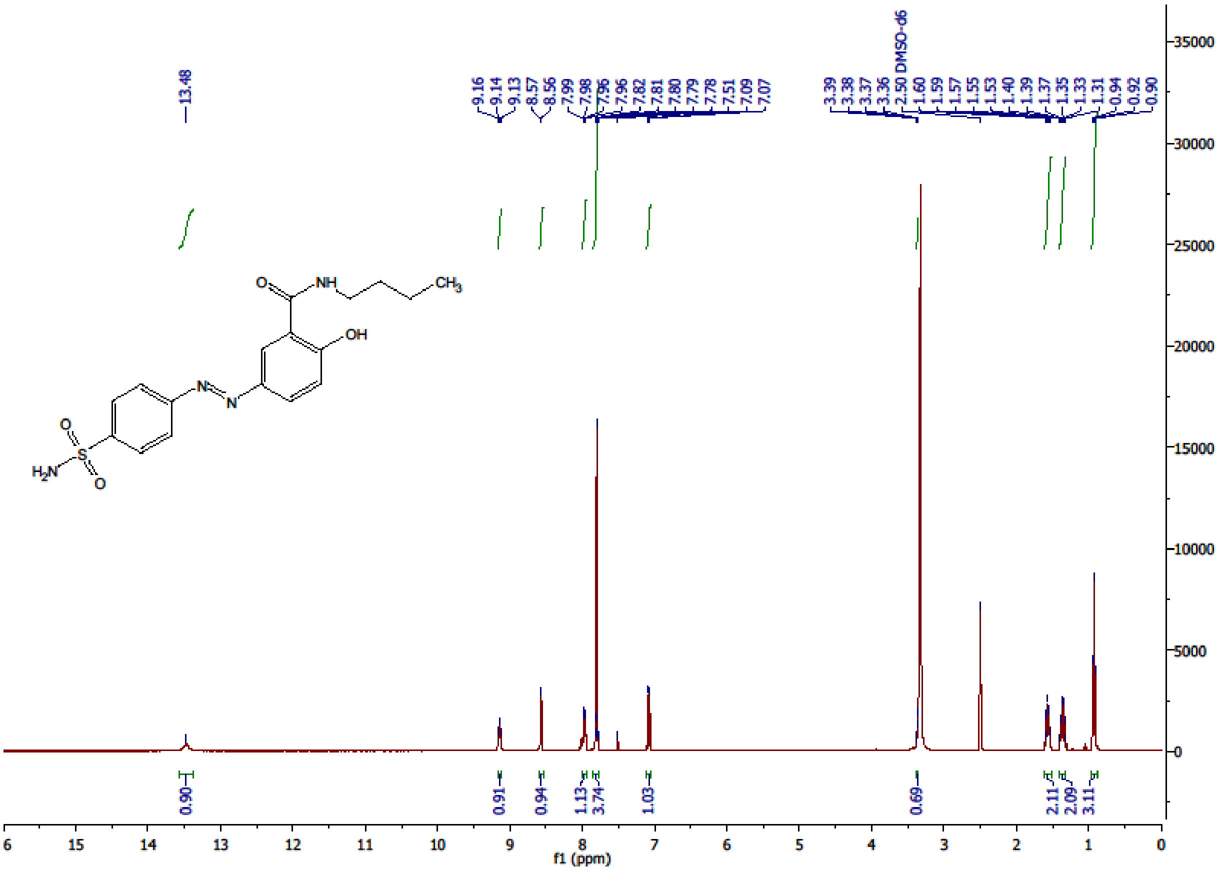

Figure 2. ^{1}**H-NMR spectrum of compound 5a (DMSO-*d*$_6$)**

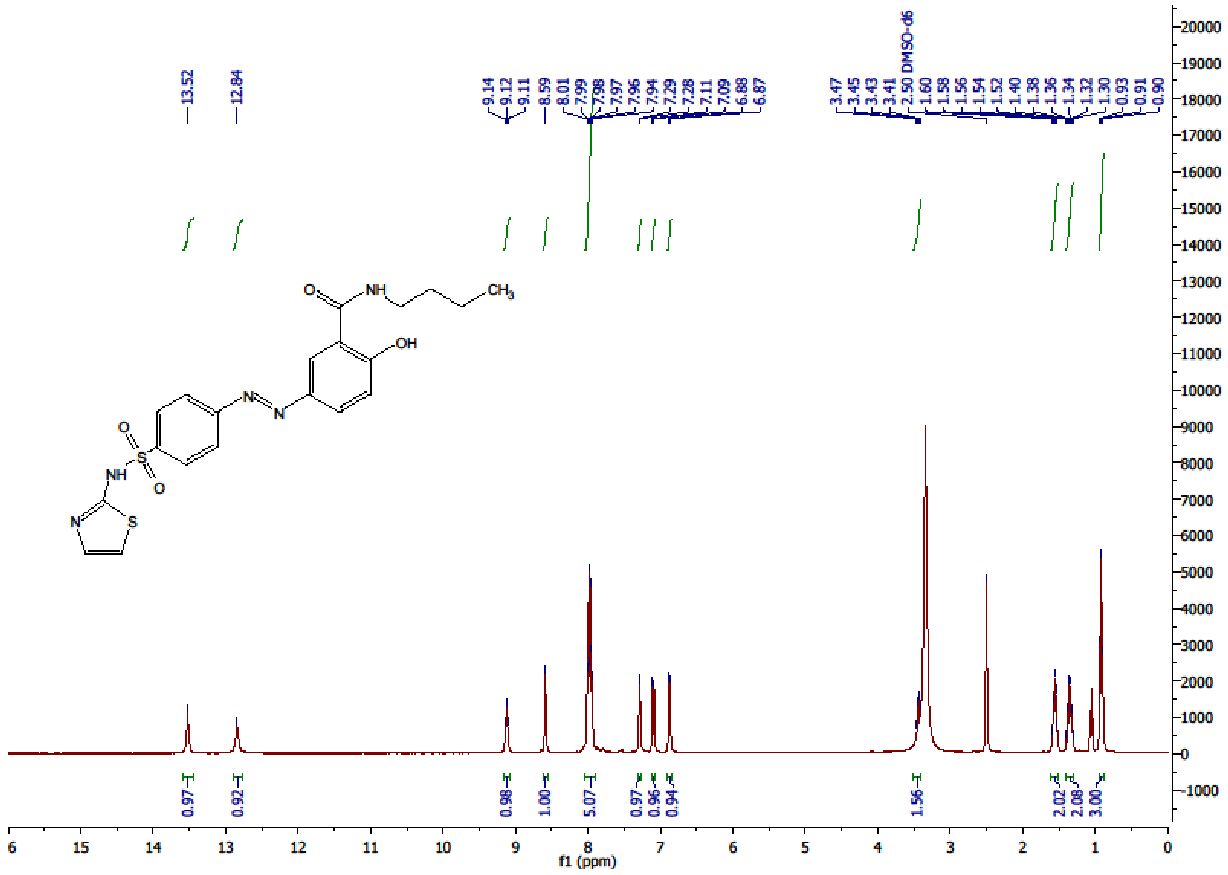

Figure 3. ^{1}H-NMR spectrum of compound 5b (DMSO-d_6)

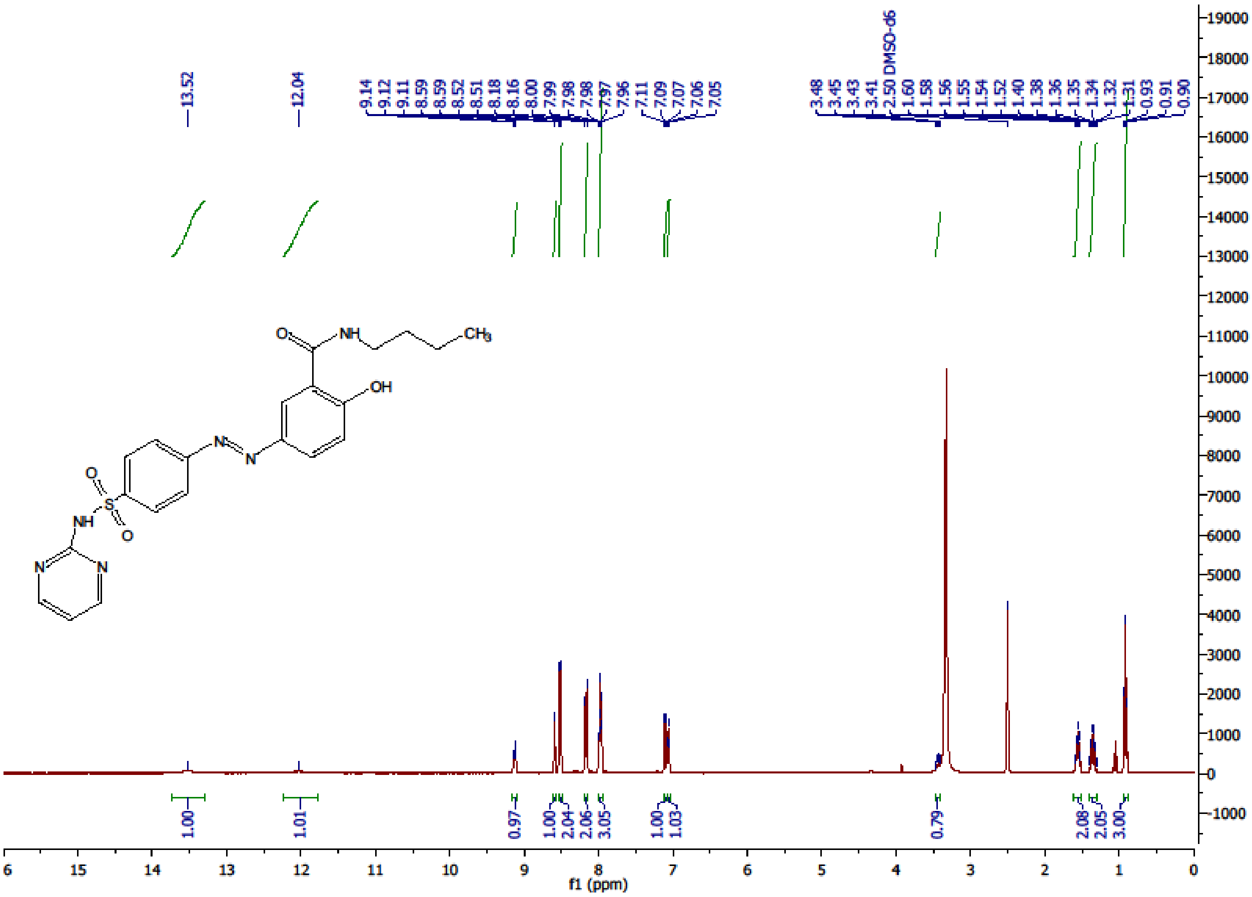

Figure 4. ^{1}H-NMR spectrum of compound 5c (DMSO-d_6)

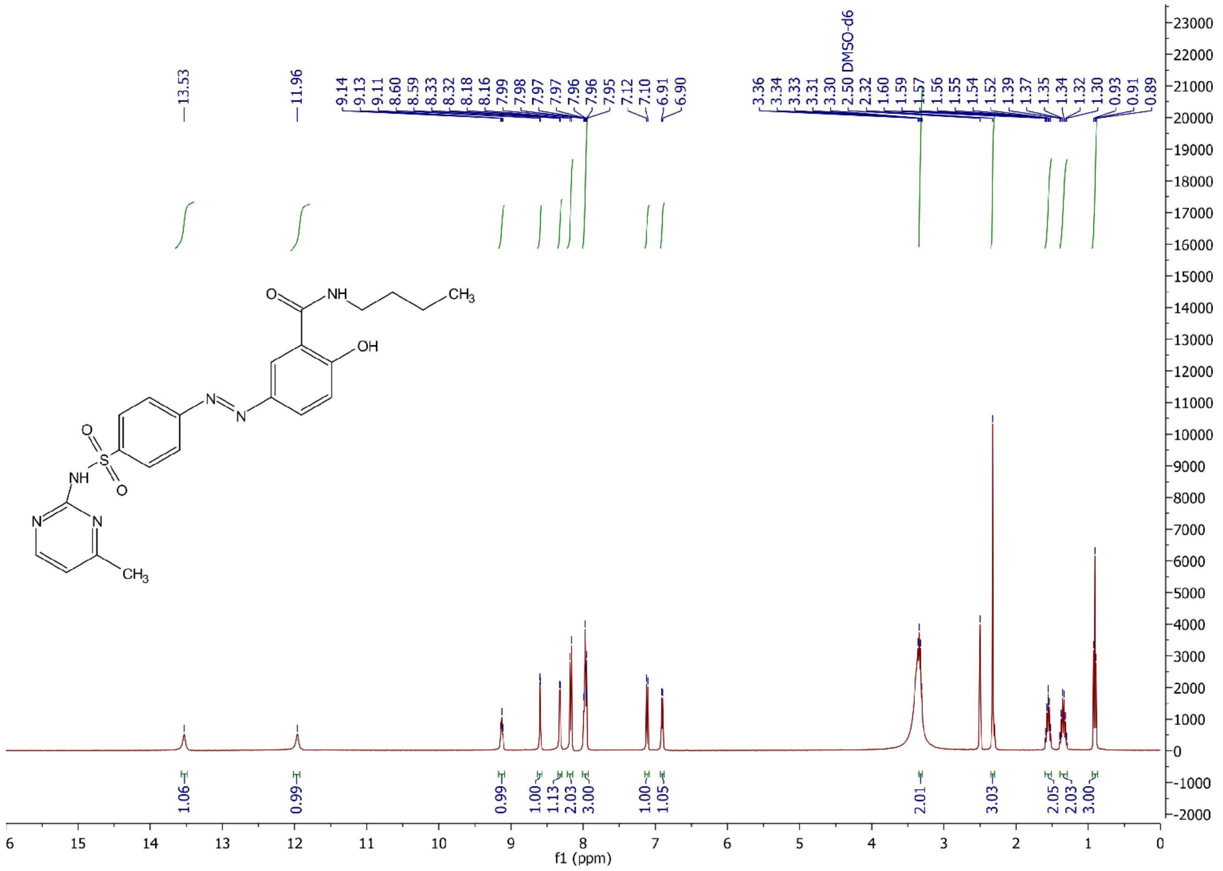

Figure 5.^{1}H-NMR spectrum of compound 5d (DMSO-*d*$_6$)

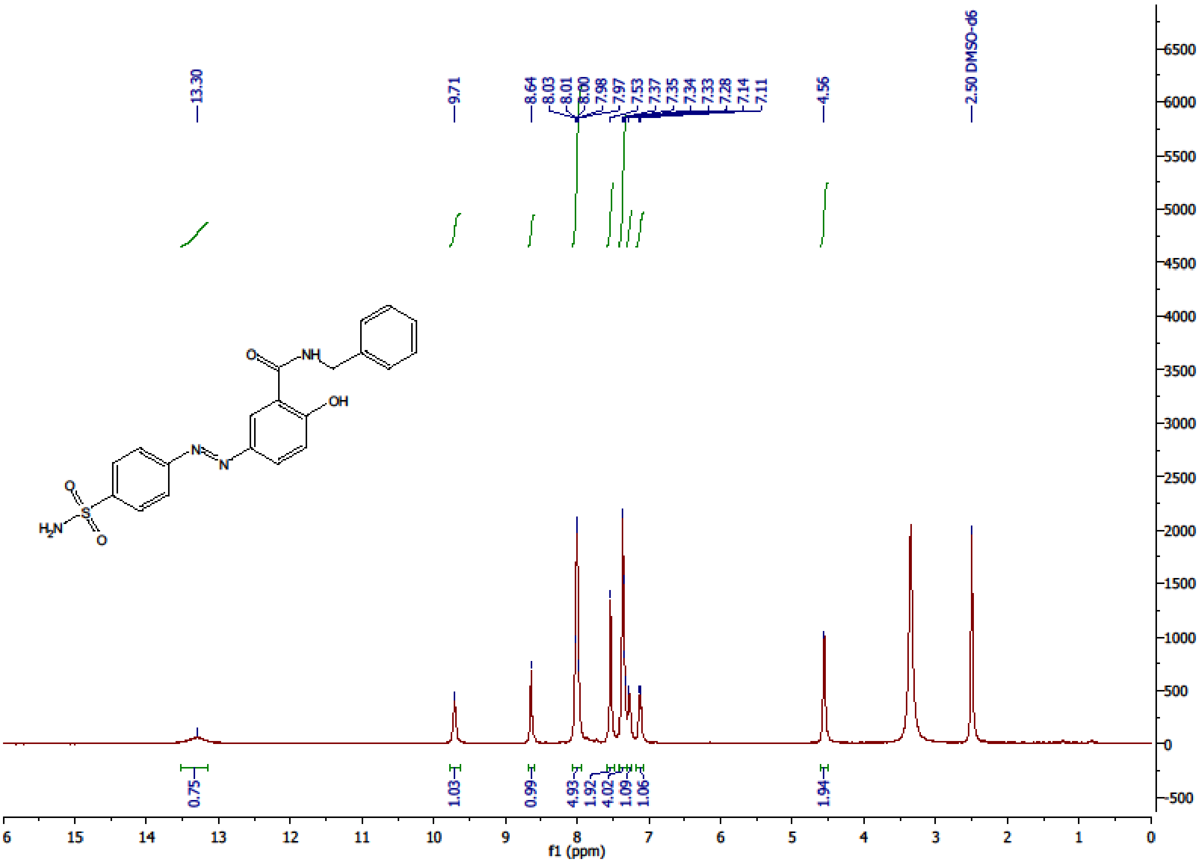

Figure 6. ^{1}**H-NMR spectrum of compound 5e (DMSO-*d*$_{6}$)**

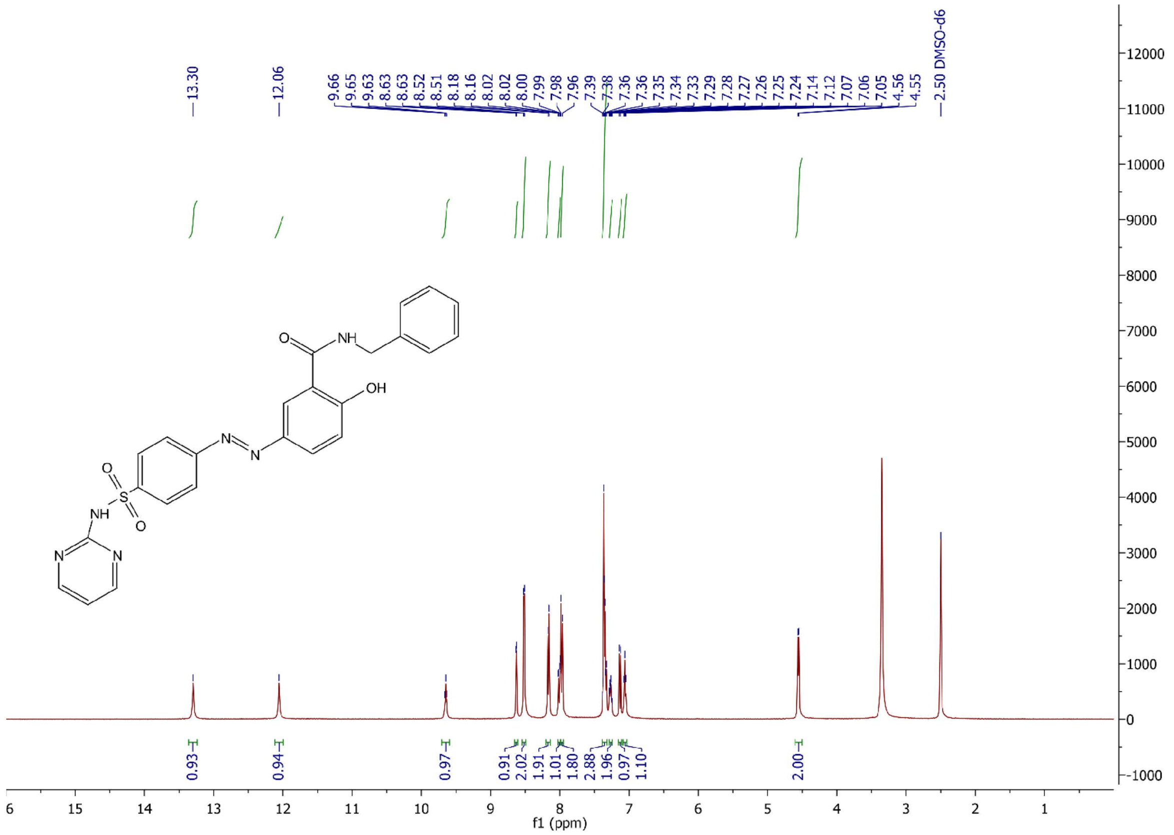

Figure 7. ^{1}H-NMR spectrum of compound 5f (DMSO-*d₆*)

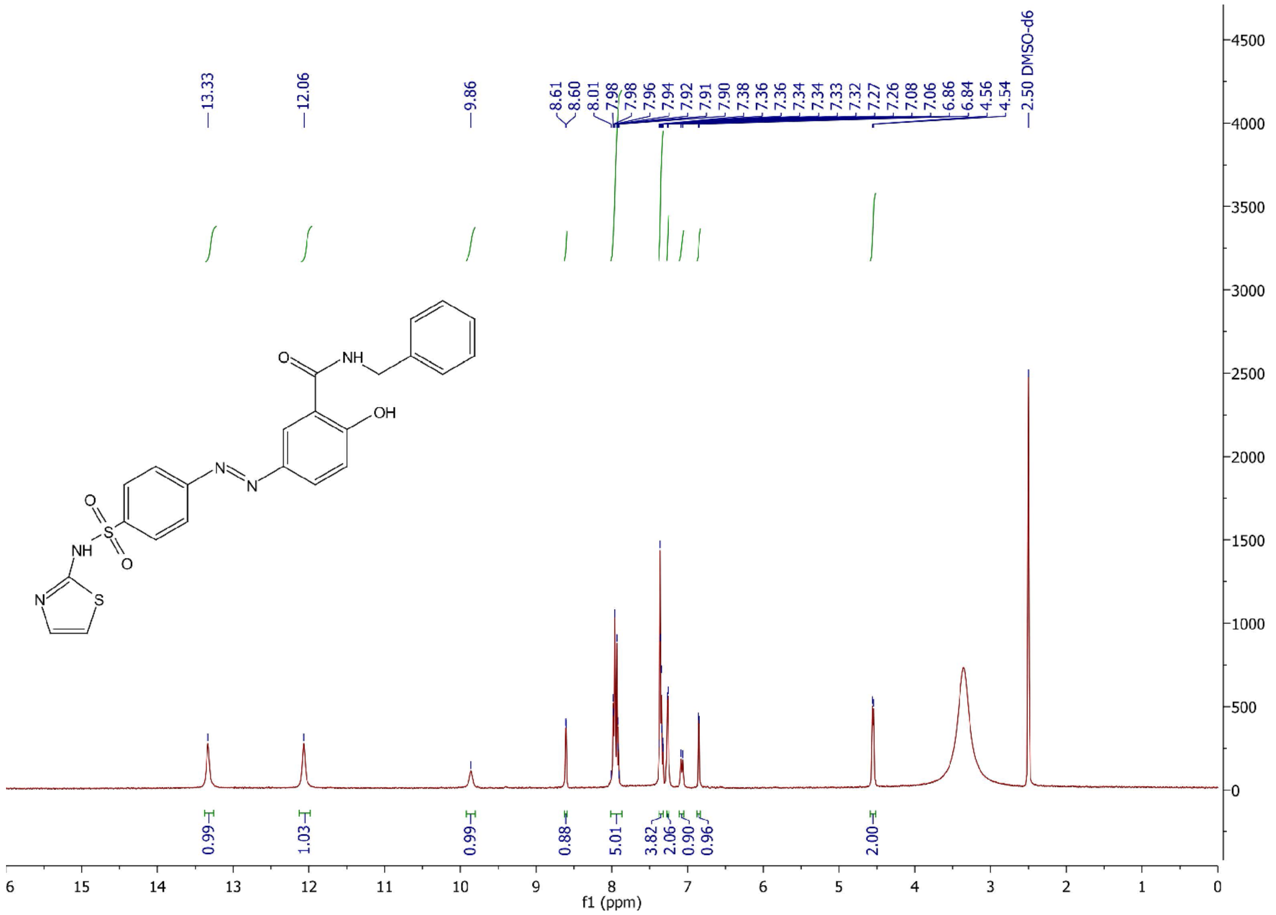

Figure 8. ^{1}H-NMR spectrum of compound 5g (DMSO-d_6)

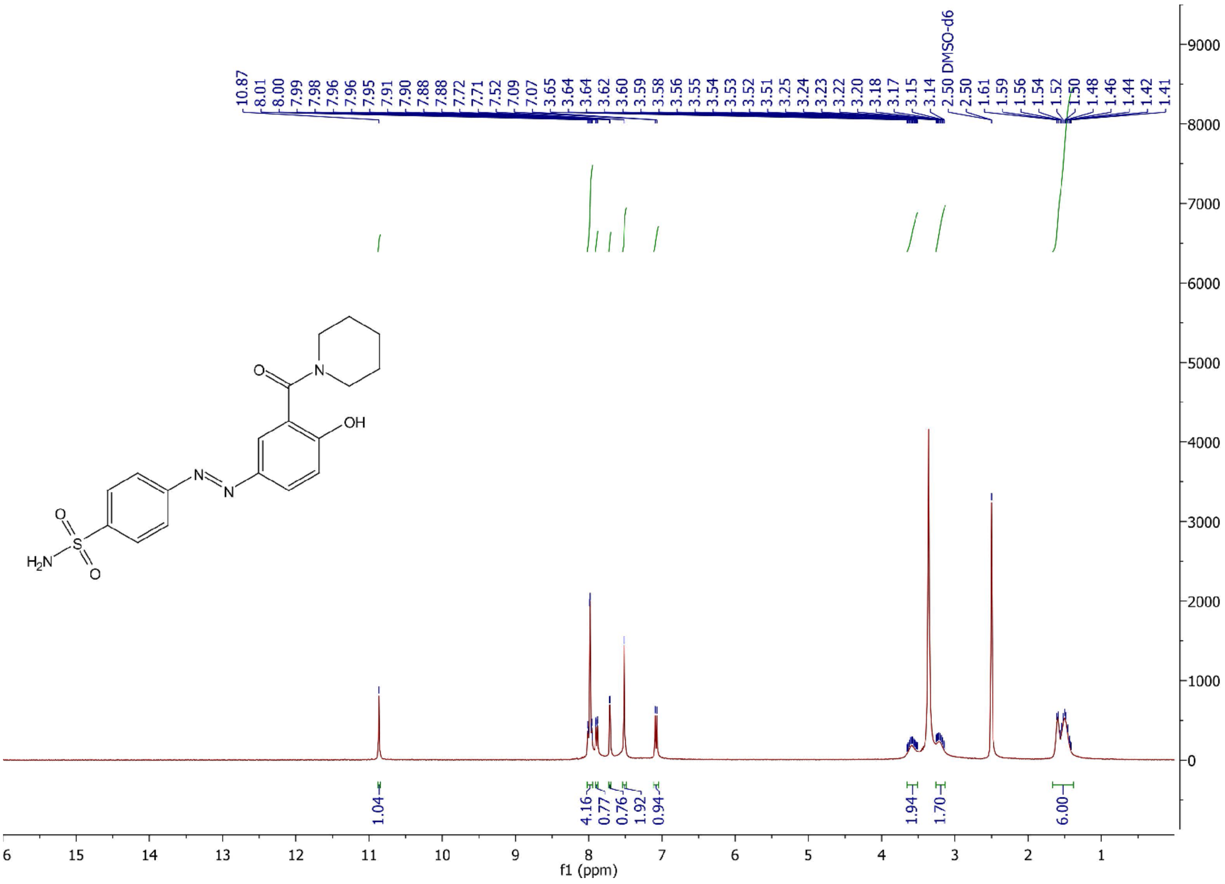

Figure 9. ^{1}H-NMR spectrum of compound 5h (DMSO-d_6)

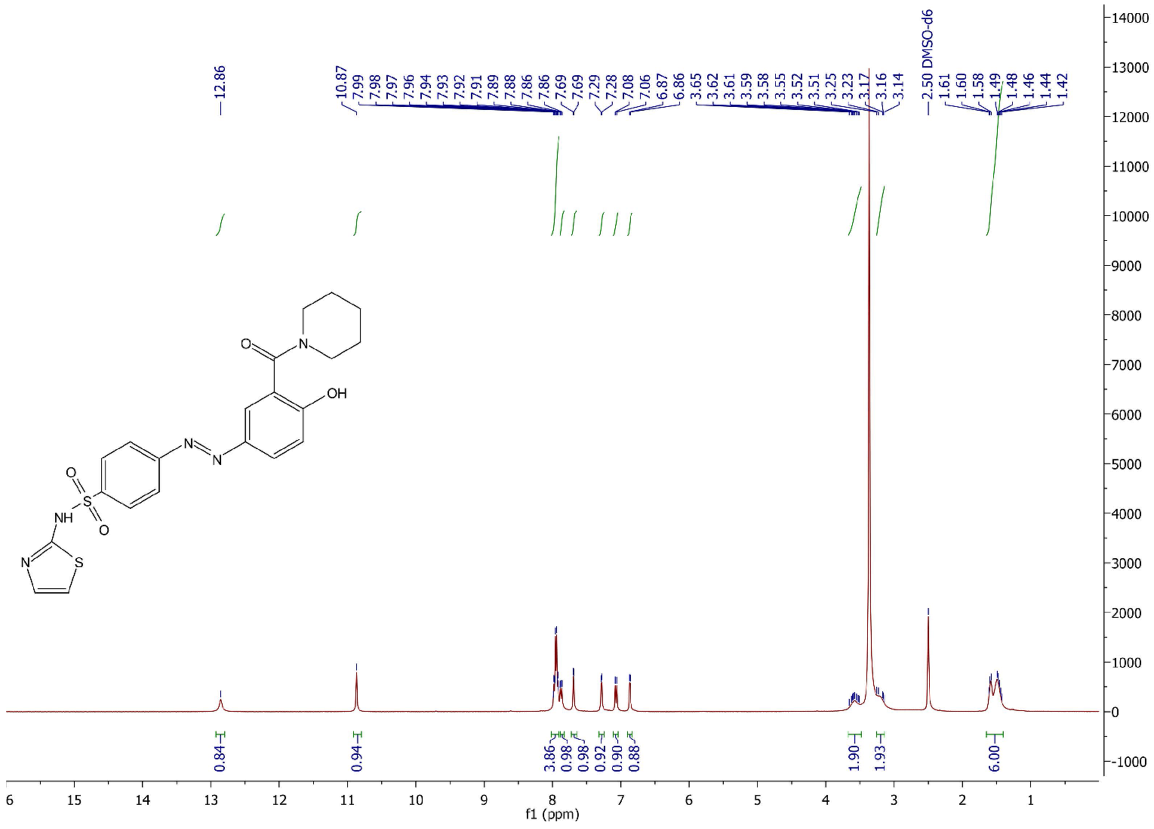

Figure 10. ^{1}H-NMR spectrum of compound 5i (DMSO-*d₆*)

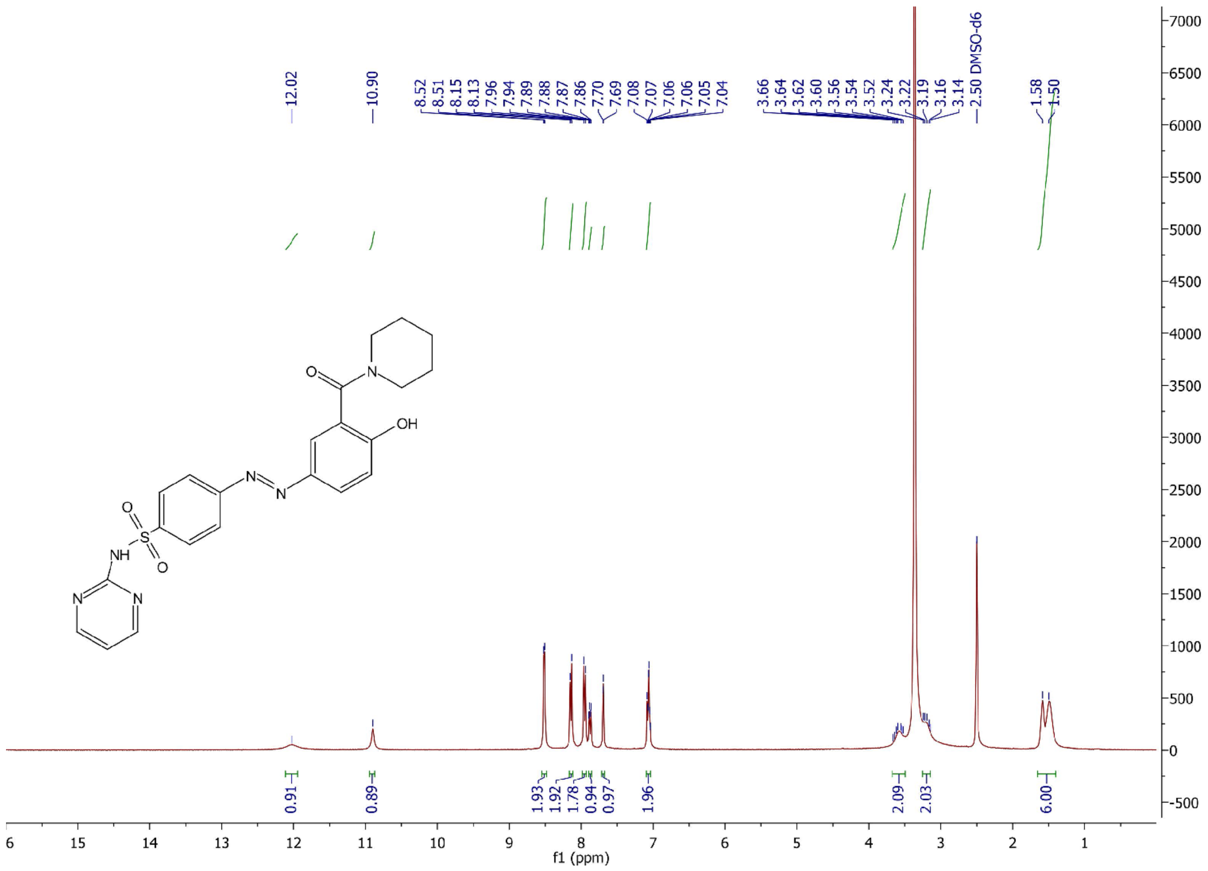

Figure 11. ^{1}H-NMR spectrum of compound 5j (DMSO-d_6)

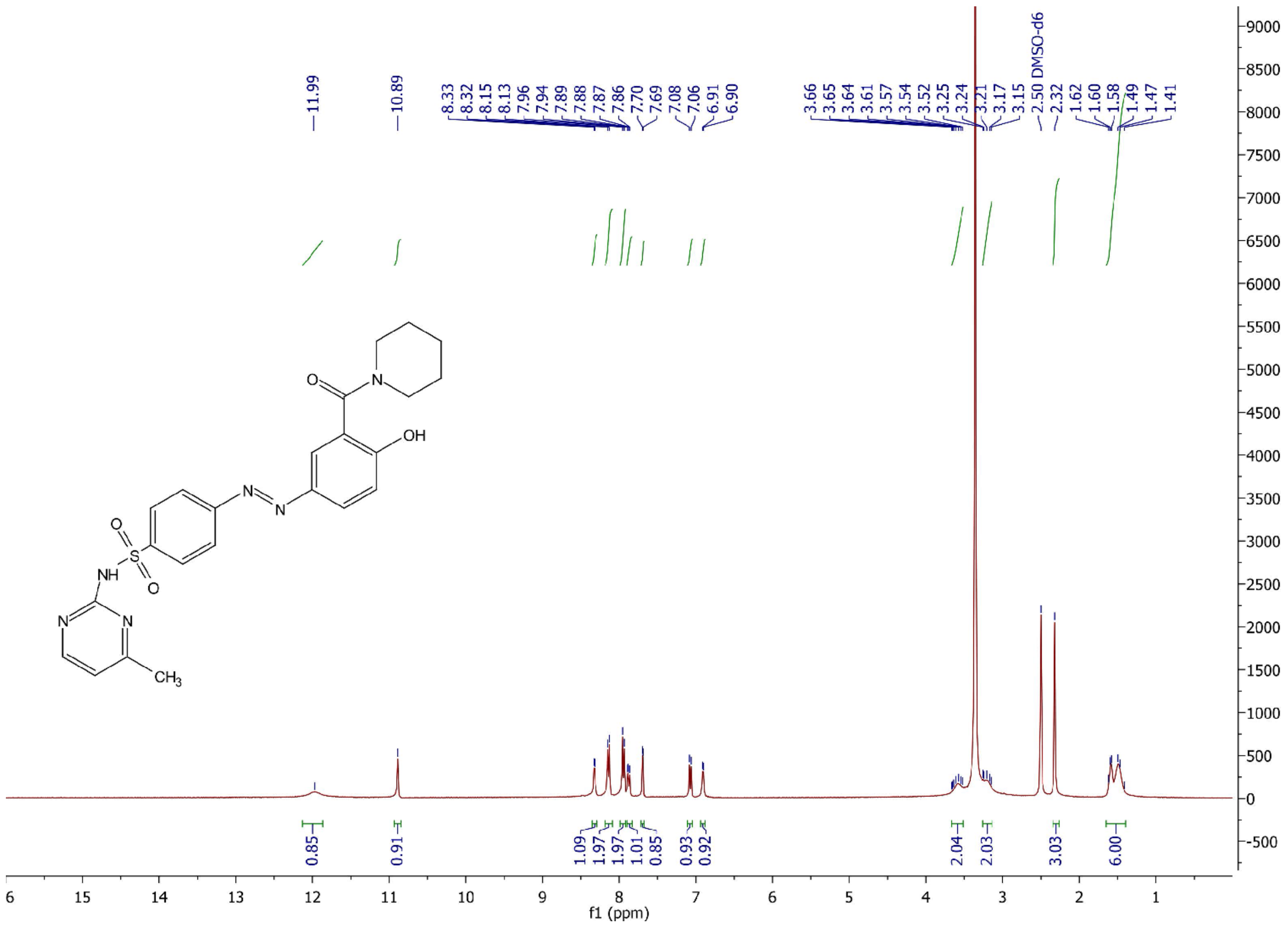

Figure 12. ^{1}H-NMR spectrum of compound 5k (DMSO-*d₆*)

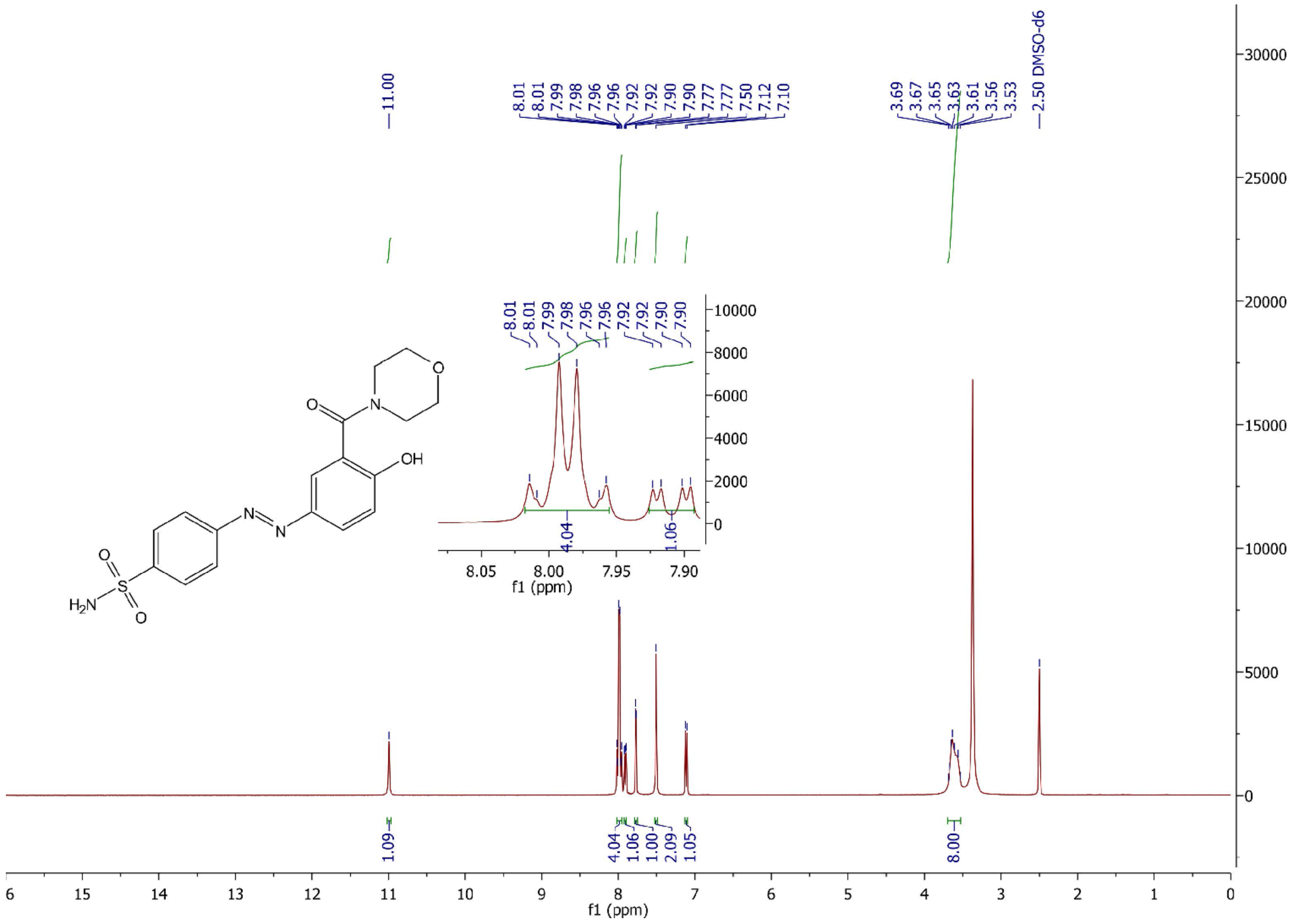

Figure 13. ^{1}H-NMR spectrum of compound 5l (DMSO-*d*$_6$)

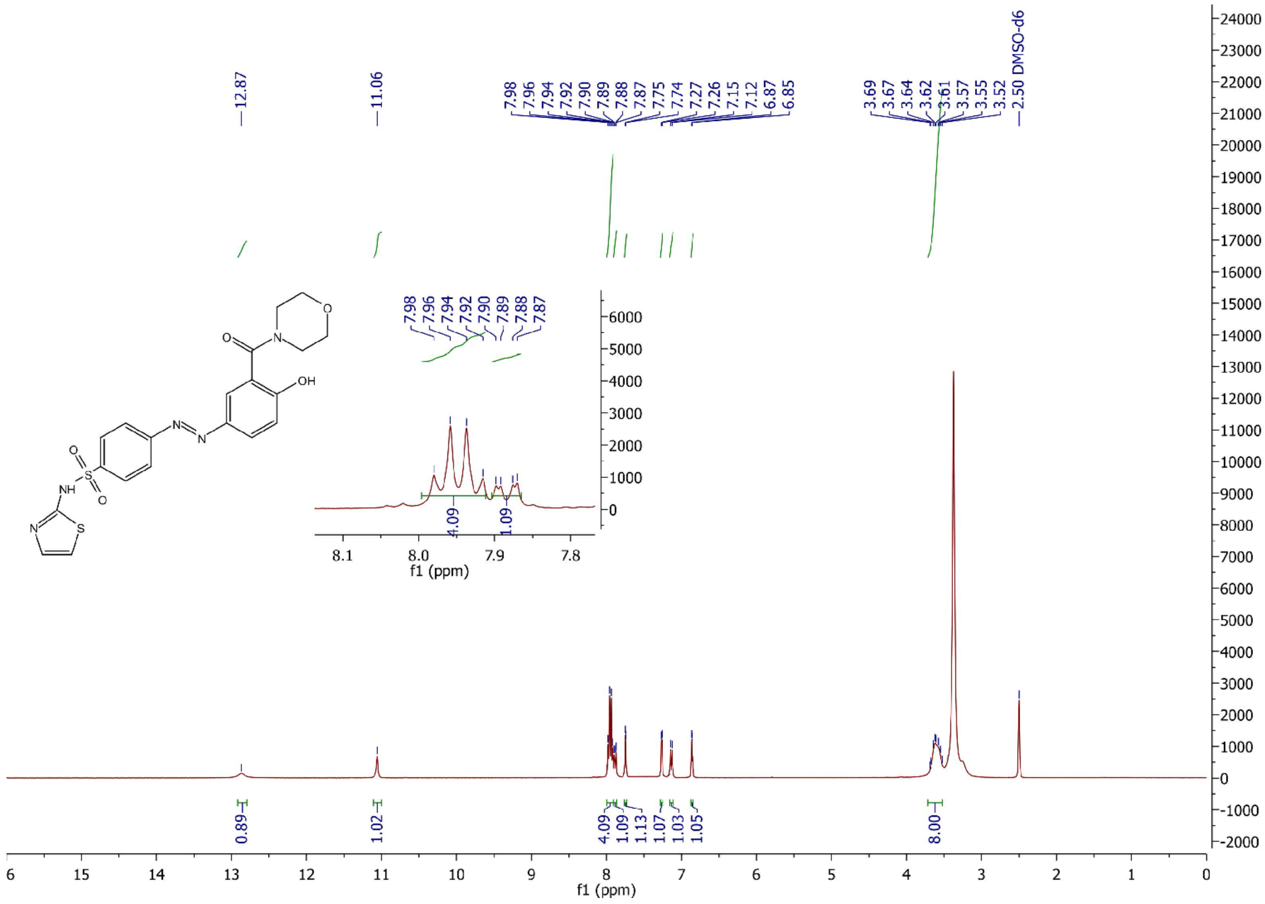

Figure 14. ^{1}**H-NMR spectrum of compound 5m (DMSO-*d$_6$*)**

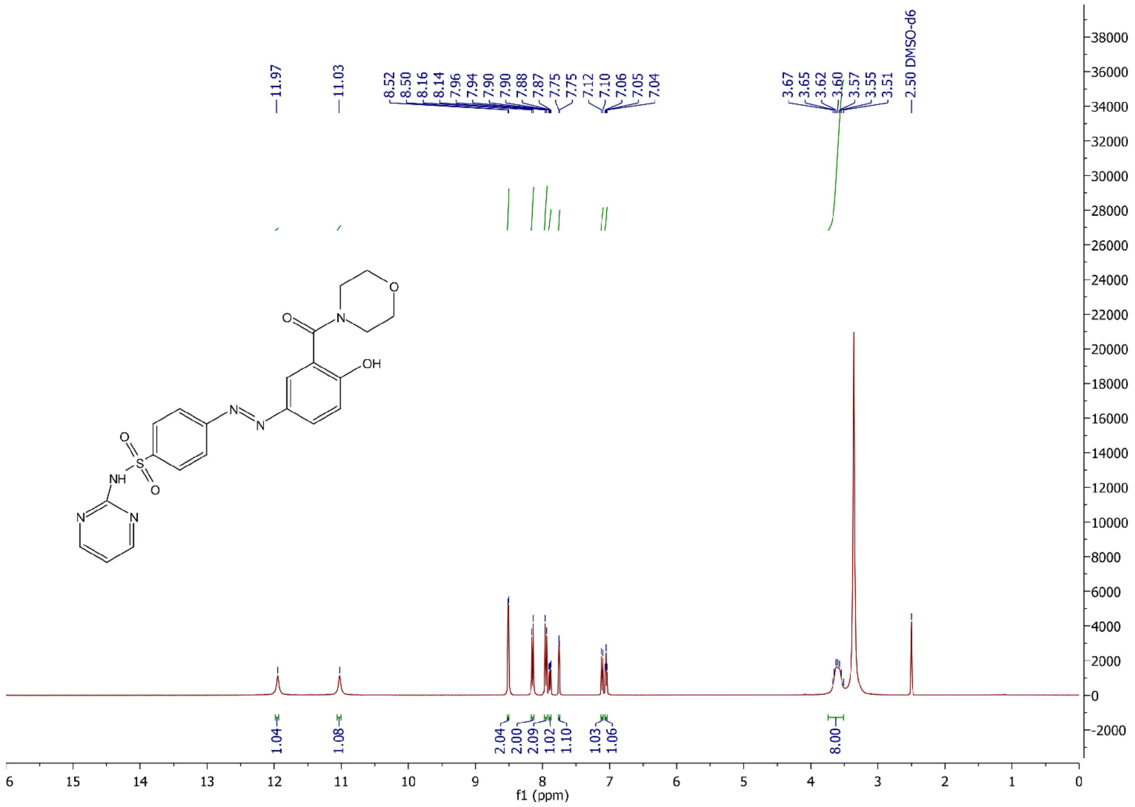

Figure 15. ^{1}H-NMR spectrum of compound 5n (DMSO-d_6)

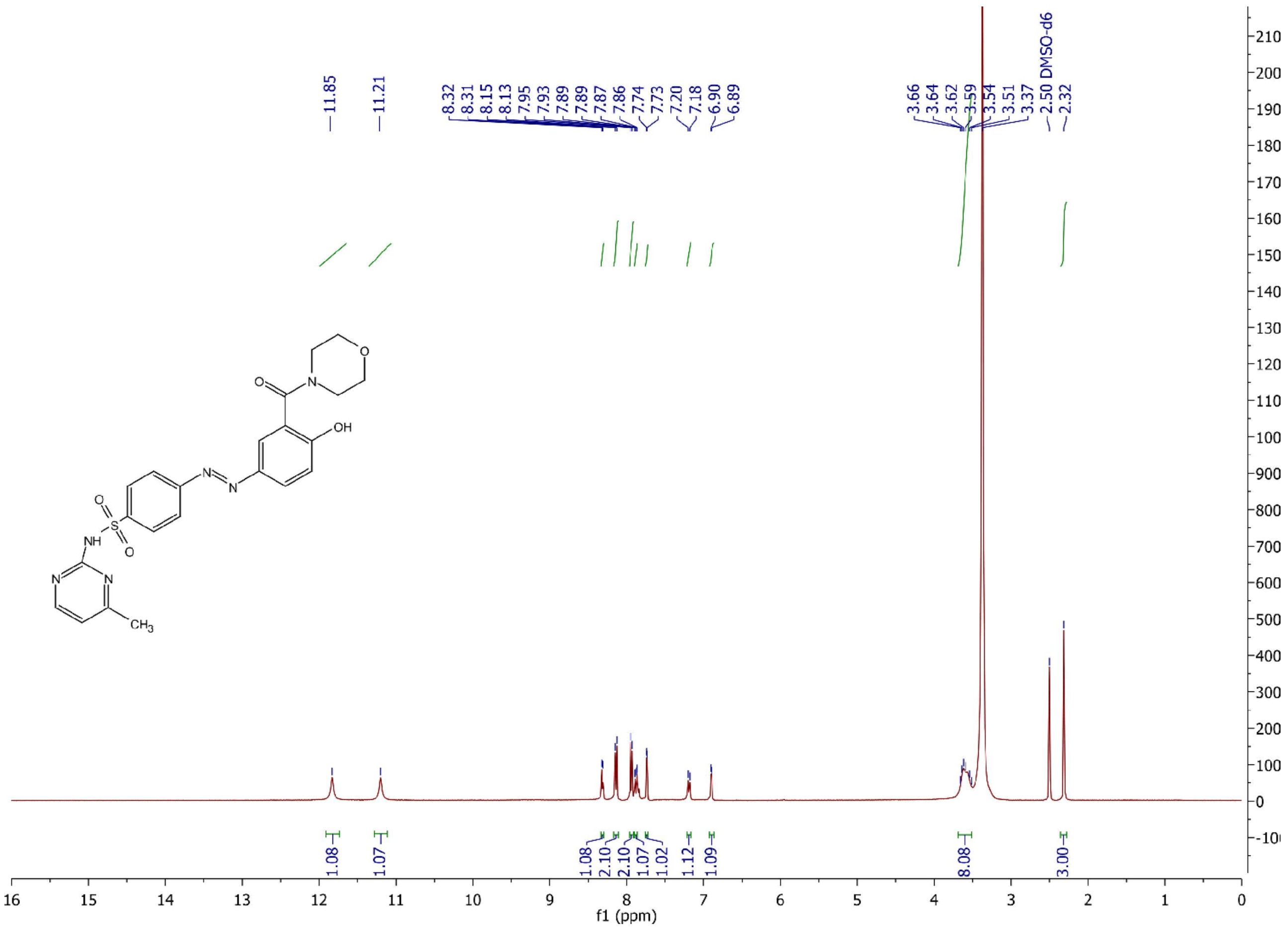

Figure 16. ^{1}H-NMR spectrum of compound 5o (DMSO-d_6)

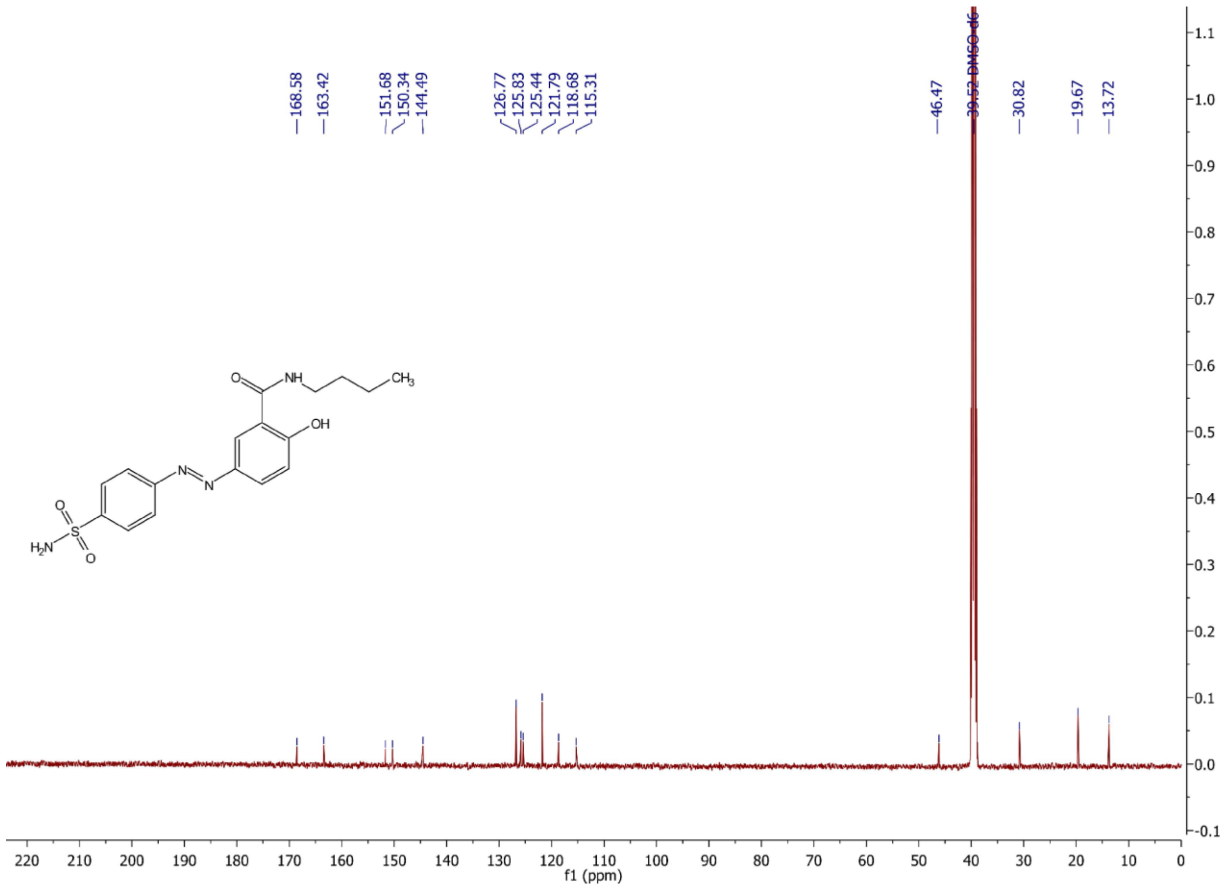

Figure 17. ^{13}C-NMR spectrum of compound 5a (DMSO-*d$_6$*)

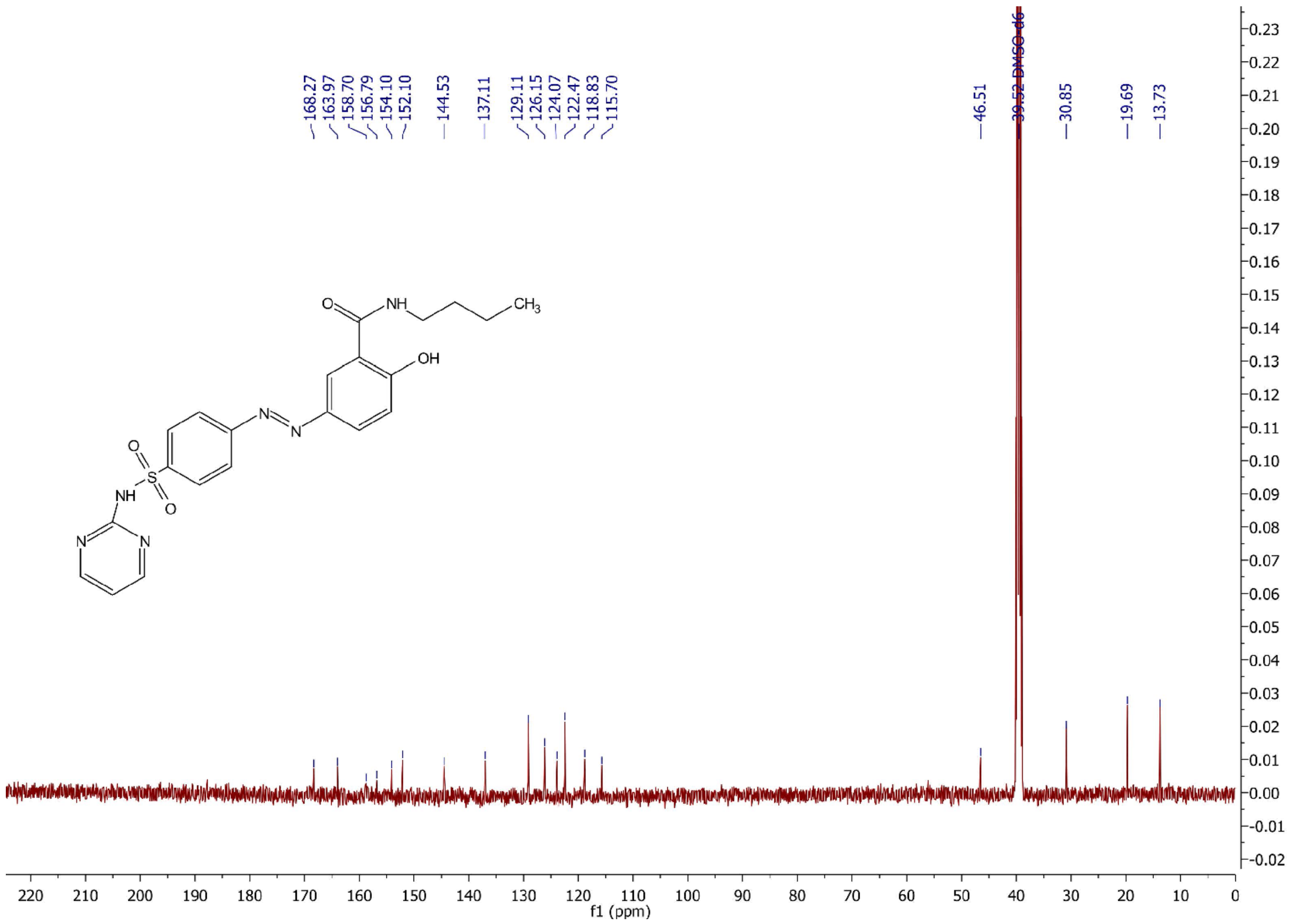

Figure 18. ^{13}**C-NMR spectrum of compound 5c (DMSO-*d*$_6$)**

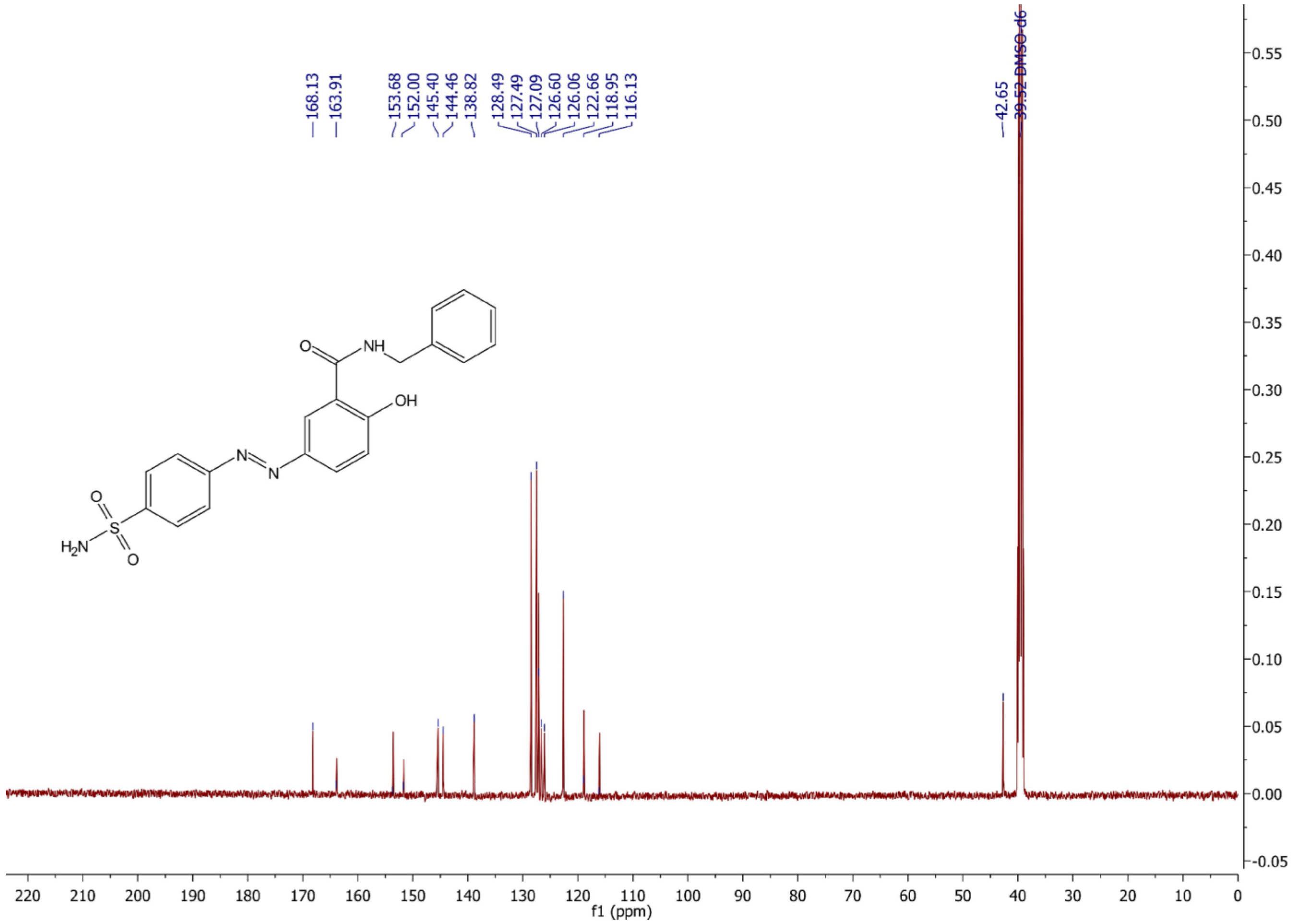

Figure 19. ^{13}C-NMR spectrum of compound 5e (DMSO-*d$_6$*)

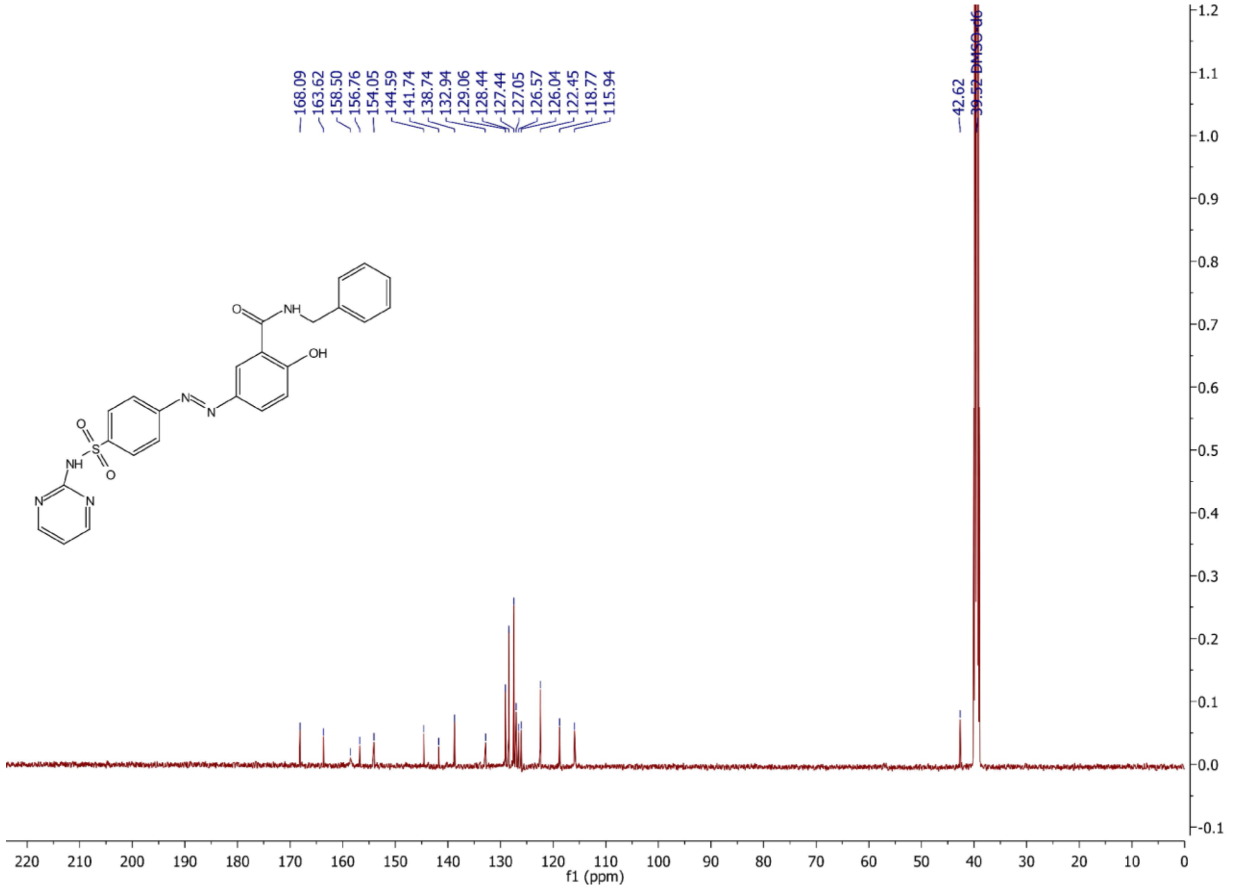

Figure 20. ^{13}C-NMR spectrum of compound 5g (DMSO-d_6)

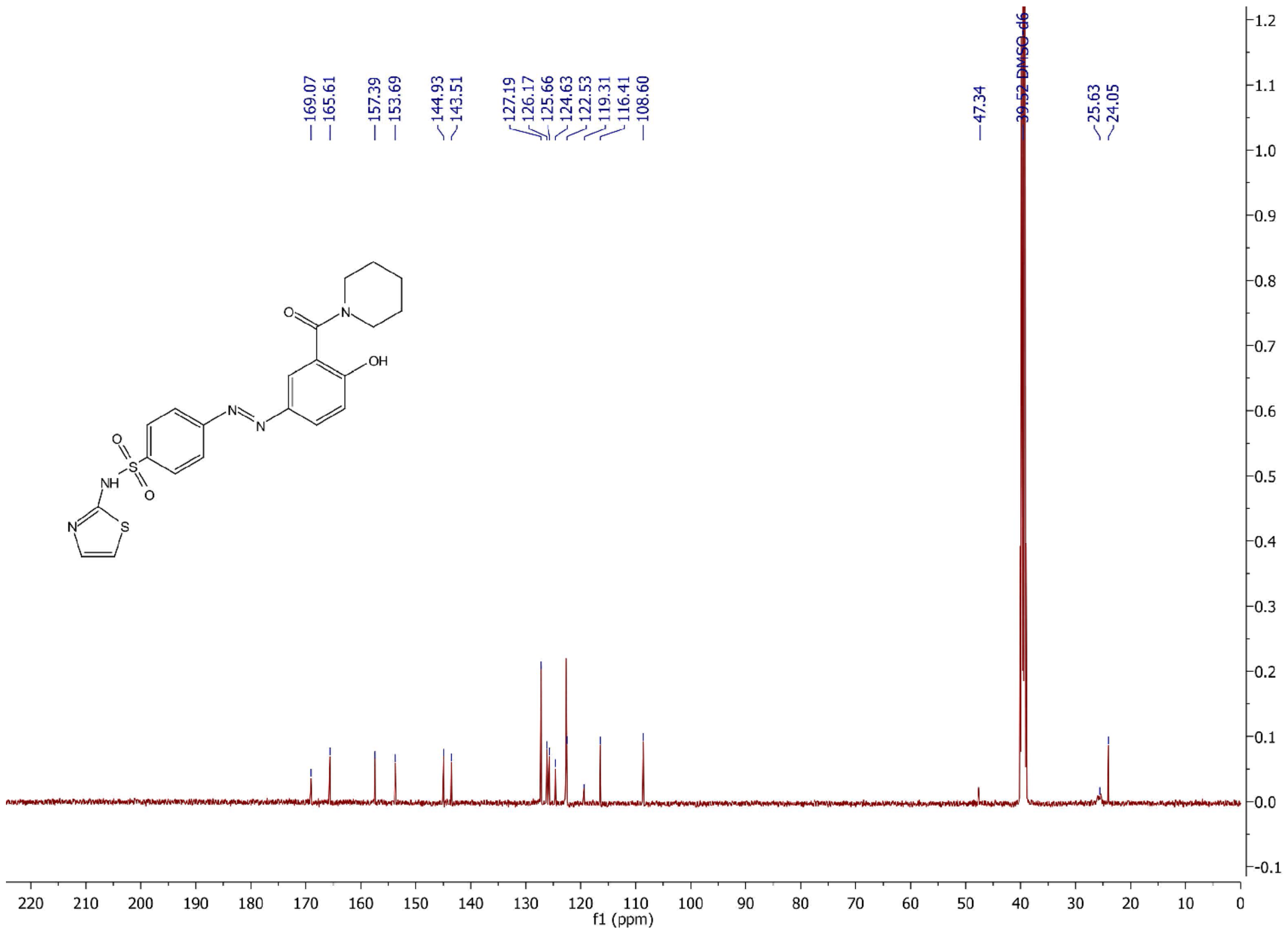

Figure 21. ^{13}C-NMR spectrum of compound 5i (DMSO-*d*$_6$)

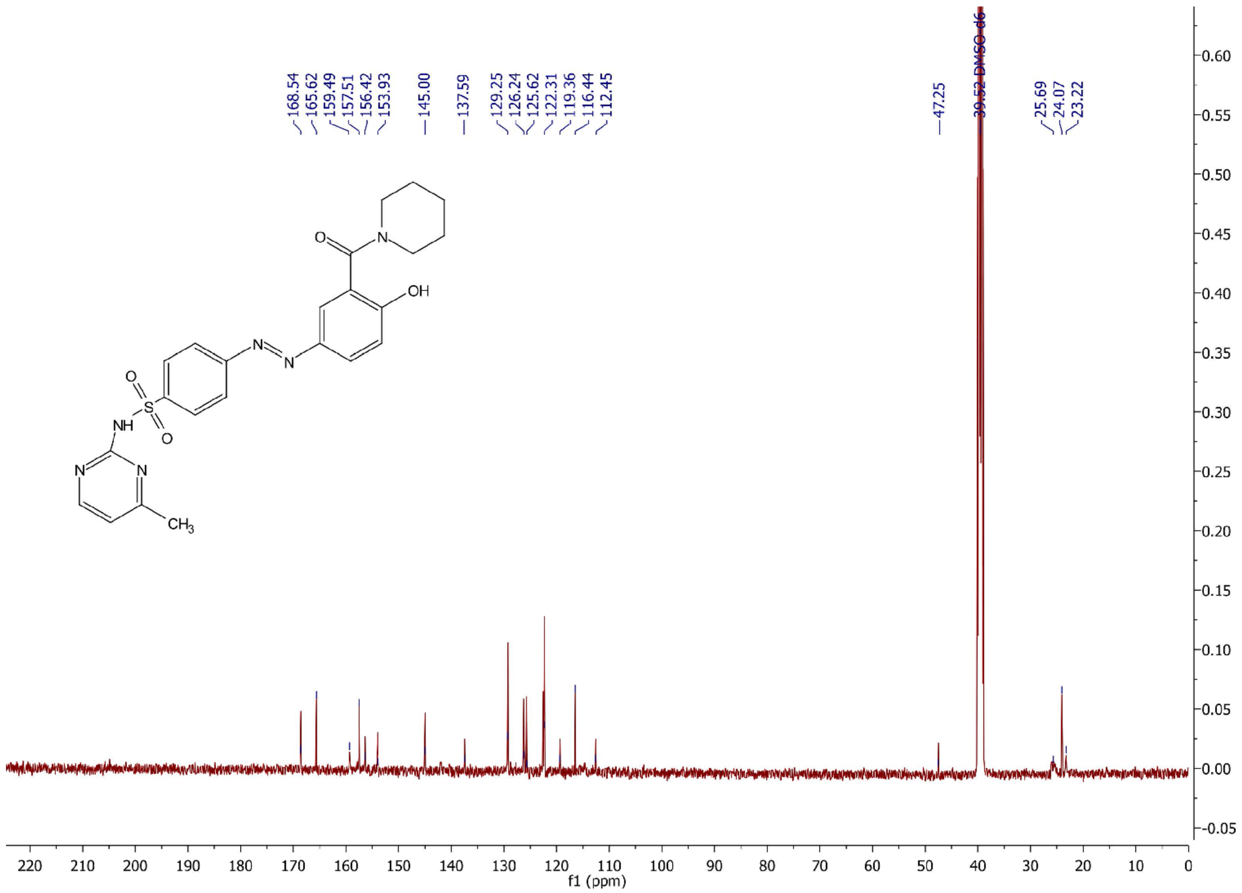

Figure 22. ^{13}C-NMR spectrum of compound 5k (DMSO-d_6)

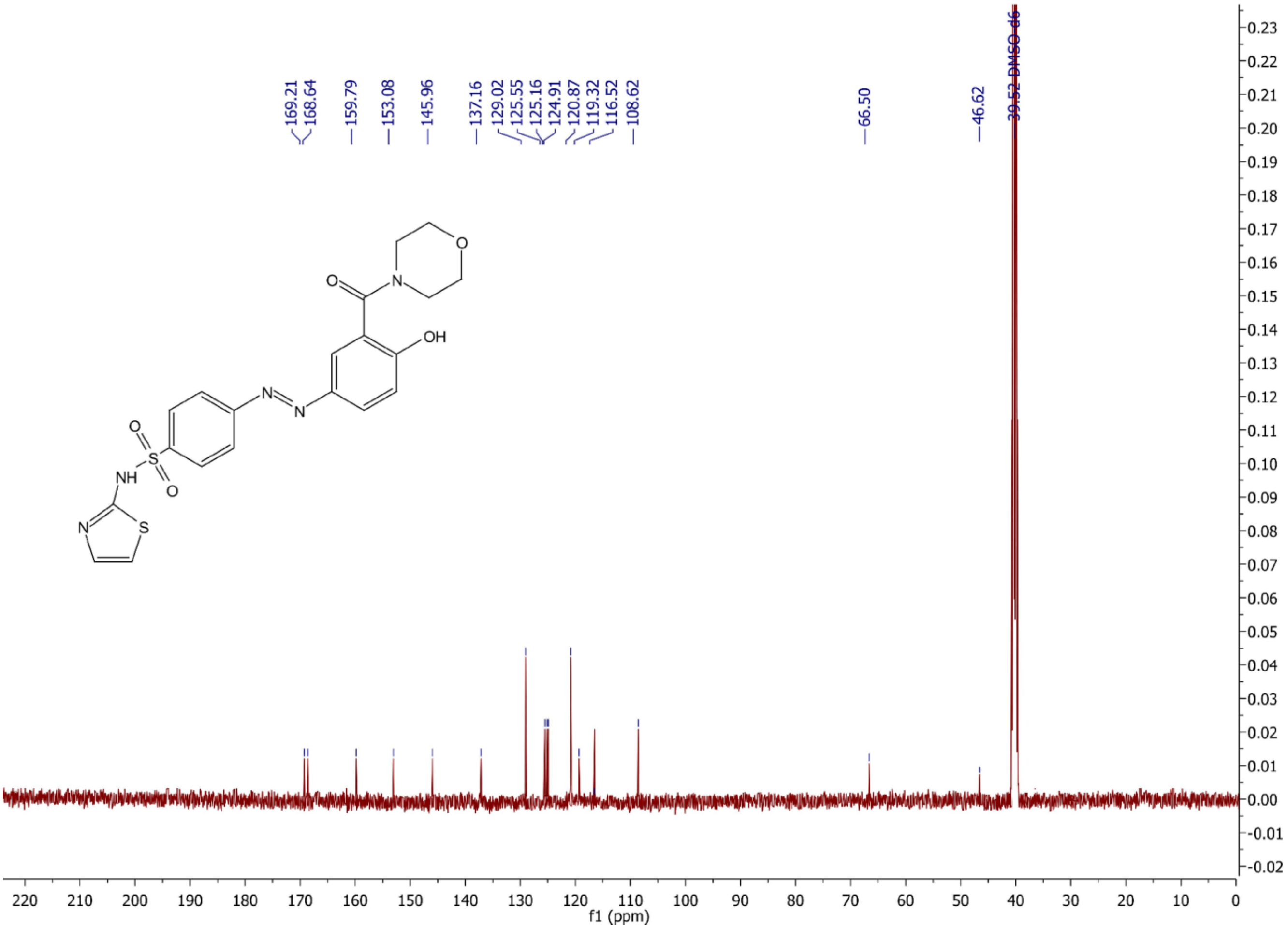

Figure 23. ^{13}C-NMR spectrum of compound 5m (DMSO-d_6)

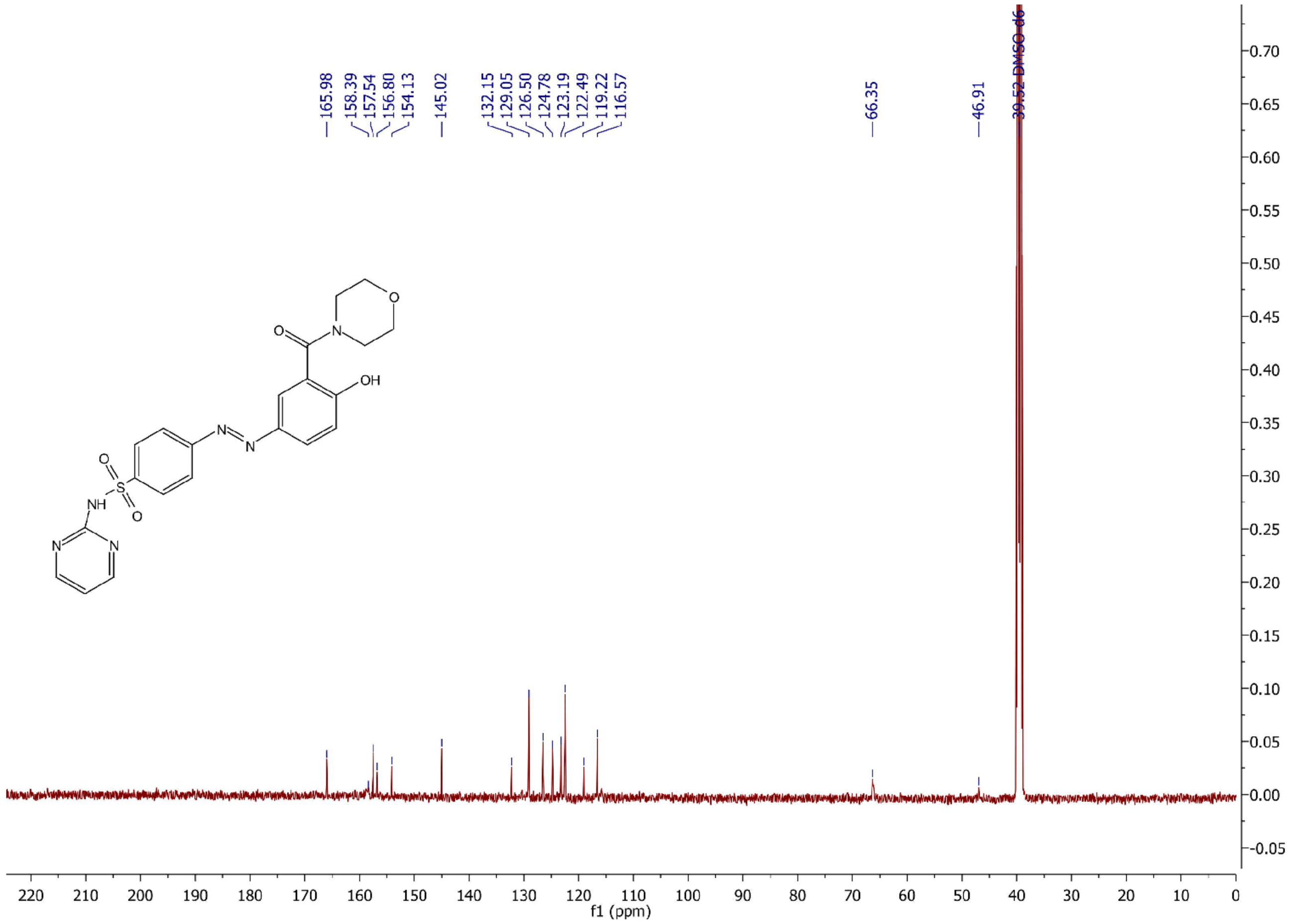

Figure 24. ^{13}C-NMR spectrum of compound 5n (DMSO-d_6)

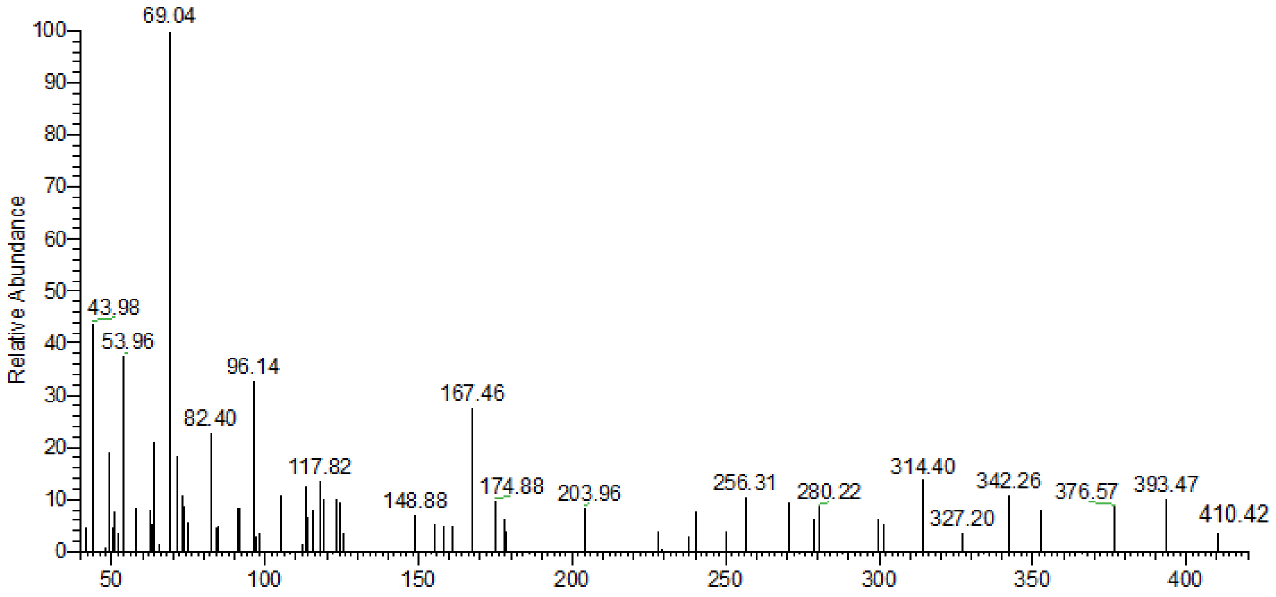

Figure 25. EI-MS spectrum of compound 5e

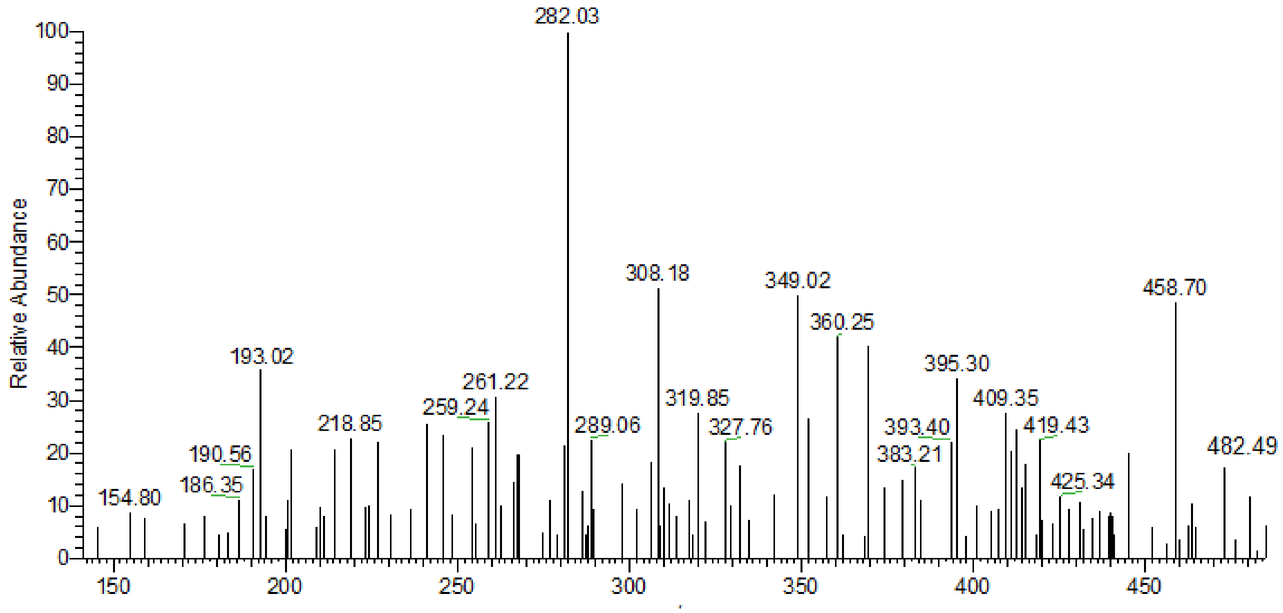

Figure 26. EI-MS spectrum of compound 5k

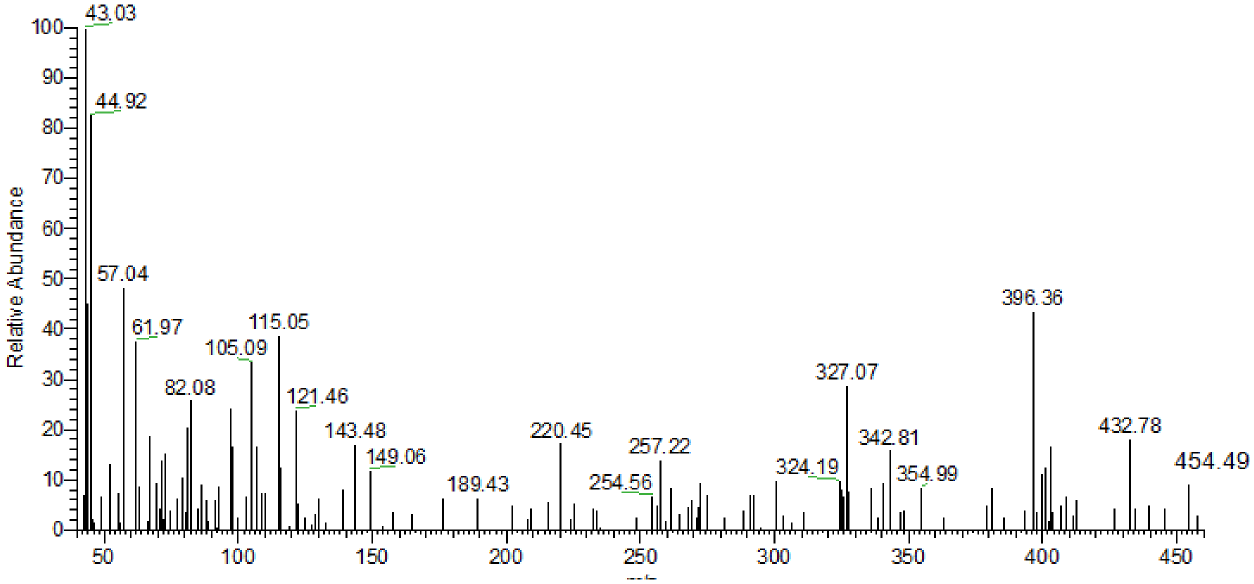

Figure 27. EI-MS spectrum of compound 5c

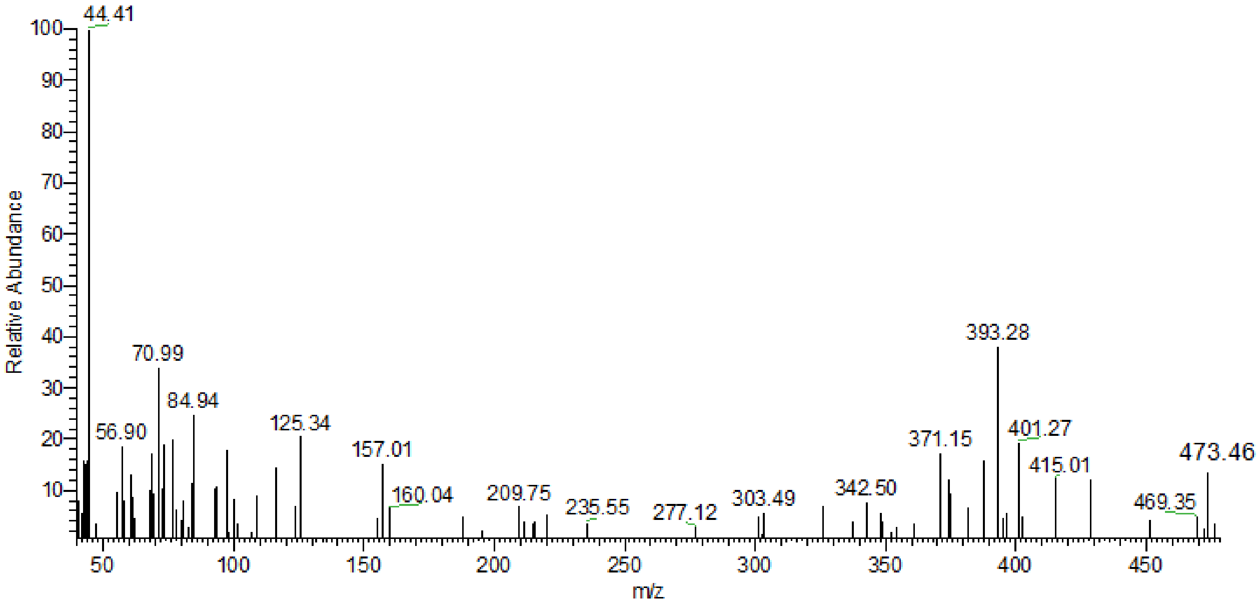

Figure 28 EI-MS spectrum of compound 5m

4.2. Biological evaluation

4.2.1. *In vitro* COX-1 and COX-2 enzymatic assay

All the synthesized target compounds were screened for their COX-1 and COX-2 inhibitory activities using Cayman colorimetric COX (ovine) inhibitor screening assay kit (Catalog No. 560131) supplied by Cayman chemicals, Ann Arbor, MI, USA. According to the manufacturer instructions and a previously reported method[55,56].The testing procedures for determining IC_{50} values of the tested compounds and reagents were prepared.

4.2.2. carrageenan-induced paw edema in mice

Swiss mice of both sexes; males and females excluding pregnant females weighing about (20-25 g) were purchased from EgyVac (Giza, Egypt) and kept in a 12 h light/dark cycle under standard conditions of temperature (24 – 26 ˚C) and humidity (50 ± 10%) with free access to food and water for 1 week before starting the experiments for adaptation. All the experimental procedures were carried out regarding to the ethical principles of the Institutional Animal Care and Use Committee (IACUC) of faculty of science, Helwan University, Egypt (approval no. hU2021/Z/AEA121-02). Mice were divided into seven groups of five mice per each; the first group acted as a negative control whose mice were administered 0.5% sodium carboxy methyl cellulose solution while the second group was given celecoxib (10 µmol/kg) as a positive control in addition to the third group

which received Diclofenac sodium as a second positive control (10 µmol/kg)[57]. The tested compounds and the reference drugs were intragastrically administered at a dose of 10 µmol/kg. Paw edema in mice was induced 1 hour after administration of the tested compounds, by injecting 0.1 mL of 1% w/v carrageenan (Sigma-Aldrich, USA) suspended in saline subcutaneously at the sub-planter surface of the right-hand paw of each mouse in all groups. The thickness (mm) of paw edema was measured by using a Vernier caliper (LETICA Scientific Instruments, Barcelona, Spain) before (0 h) and after carrageenan injection at 2, 4 and 8 h and compared to that of the negative control group to estimate the antiinflammatory activity. The percentage of paw-edema inhibition (antiinflammatory activity; AI %) was calculated by the formula:

$$(AI\ \%) = \frac{(C_t - C_0)\ \text{control} - (C_t - C_0)\ \text{tested compound}}{(C_t - C_0)\ \text{control}} * 100$$

C_t = the average thickness of right paw edema at specific time interval after carrageenan treatment

C_0 = the average thickness of right paw edema at zero-time interval before carrageenan treatment.

4.2.3. Determination of ED_{50}

Compounds **5b, 5j, 5n** and **5o** were further screened at doses of 5, 10, 20, 30 and 40µmol/kg body weight and the dose-response curves was used to determine the ED_{50} (median effective dose) values by measuring the inhibition of the edema volume after 8 hours of carrageenan injection[58].

4.2.4. Estimation of rat plasma prostaglandin E2 (PGE2)

Five blood samples were collected from rats 8hours after carrageenan injection and centrifuged to separate plasma, immediately frozen and stored until use. A competitive immunoassay procedure was applied for quantitative determination of PGE2 in biological fluids with EIA PGE2 kit (Aldrich, Steinheim, Germany). After incubation at room temperature, a monoclonal antibody to PGE2 in kit was used to bind to PGE2 in the sample competitively. Excess reagents were washed away, and then the substrate was added and incubated for short time until generation of yellow color. The optical density was recorded using a microplate reader DYNATech, MR 5000 (Dynatech Industries Inc., McLean, VA) at 450nm, and expressed in pg/ml[59].

4.2.5. Gastric ulcerogenic activity

The acute gastric ulcerogenic effect of compounds **5b, 5j, 5n** and **5o** in adult male Wistar rats was evaluated[58]. Rats weighing from 200 to 250 g were divided into seven groups of five rats each and fasted for 12 h before carrying out the experiment. Control group received only the vehicle saline, while other groups received celecoxib and Diclofenac sodium as reference drugs as well as the test compounds orally at a dose of 30µmol/kg body weight, dosed twice at 4h interval. After Six hours of the treatment of the last dose they were killed by ether inhalation, their stomachs were removed, opened along the greater curvature, washed under running water, fixed in saline solution and examined for the presence of hyperemia, hemorrhage or gastric ulcers. In addition, histopathological examination was also carried out to confirm the inflammatory reaction degree in the gastric layers of the treated rats' stomachs.

4.2.6. Antibacterial screening

4.2.6.1. Inhibition-zone measurements

Agar cup diffusion technique[59] was used for evaluation of the antibacterial activity of all synthesized compounds using a 1 mg/mL solution in DMSO. The test was performed on Gram-positive bacteria as *Staphylococcus aureus (RCMB 0100183), Staphylococcus epidermidis (RCMB 0100183), Streptococcus mutans (RCMB 0100172),* and *Bacillus subtilis (RCMB 0100162)* as well as Gram-negative bacteria as *Pseudomonas aeruginosa (RCMB 0100243), Escherichia coli (RCMB 010052), Salmonella typhi (RCMB 0100104), Shigella dysenteriae (RCMB 0100542)* and *Proteus vulgaris (RCMB 010085)*. Each 100 mL of sterile molten agar (at 45˚C) received 1 mL of 6 h-broth culture and then the seeded agar was poured into sterile Petri dishes. Cups (8 mm in diameter) were cut in the agar. 0.1 mL of the 1 mg/mL solution of the test compounds was poured on each cup. The plates were then incubated at 37˚C for 24 h. A control using Dimethylformamide (DMF) without the test compound was included for each organism. Ampicillin was used as standard Gram-positive antibacterial reference while levofloxacin was used as standard Gram-negative antibacterial reference. The resulting inhibition zones were recorded in (Table 4, page S5 supplementary file).

4.2.6.2. Minimal inhibitory concentration (MIC) measurement

Two fold serial broth dilution method[60] was used to measure the minimal inhibitory concentrations (MIC) of the test compounds. A suitable broth: 24 h at 37˚C was used to grow the test organisms. Two fold serial dilutions of solutions of the test compounds were prepared using 200, 100, 50, 25, 12.5, 6.25 and 3.125 µg/mL. The tubes were then inoculated with the test

organisms; each 5 mL received 0.1 mL of the above inoculum and were incubated at 37˚C for 48 h. Then, the tubes were observed for the presence or absence of microbial growth. The MIC values of the prepared compounds are listed in (Table 5, page S5 supplementary file).

4.2.6.3. Minimal bactericidal concentration (MBC) measurement

MBC tests were always measured following to the MIC as follows[63]: A loop-full from the tube that did not show visible growth (MIC) was spread over a quarter of Müller–Hinton agar plate. After incubation for 18 h, the plates were examined for growth. Again, the tube containing the lowest concentration of the test compound that failed to yield growth on subculture plates was judged to contain the MBC of that compound for the respective test organism (Table 5, page S5 supplementary file).

4.2.7. *In vivo* antibacterial screening in mice (Bacteremic infection)

To investigate the *in vivo* antibacterial activity of compounds **5b, 5j, 5n** and **5o** against *E.coli* and *S. aureus* using **sulfasalazine** as a reference drug, 352 mice of both sexes (except pregnant females) with weights ranging from 20 to 25gm were divided into 14 groups; 7 groups for *E.coli* (**5b** group, **5j** group, **5n** group, **5o** group, **sulfasalazine** group, positive control and negative control) and the other 7 groups for *S. aureus*. The 32 mice in **5b** group were divided into 4-dose group each group containing 8 mice of equal numbers of males and females. Likewise for **5j** group, **5n** group, **5o** group and **sulfasalazine**, for positive and negative control groups, each contains 8 mice; half males and half females. The mice in each dose group were subjected to intragastric administration of different dosage (100, 300, 500, 600 µmol/kg) of compound **5b** and so on for **5j**, **5n**, **5o** and **sulfasalazine**[64]. For *E.coli* mice groups, the mice in each dose group as well as the positive control were injected intraperitoneally with a single 0.5ml portion of *E. coli* C11 bacterial suspension in a concentration of 7.1×10^3 CFU per mouse 1 hour after oral treatment with compounds. Similarly, the mice in each dose group as well as the positive control were injected intraperitoneally with a single 0.5ml portion of *S. aureus* Smith bacterial suspension in a concentration of 9.5×10^6 CFU per mouse, for *S.aureus* mice groups. Untreated animals were easily subjected to 100% mortality during a period of 14 and 72 h after infection. Drugs were given to mice once a day for 7 continuous days. Survival of the mice was recorded every day over a period of seven days and the median effective dose **ED_{50}** was calculated for each group[65]. At the end of the experiment, all mice were euthanized as per the adopted Animal welfare and experimental

procedures and in accordance with the "Guide for the Care and Use of Laboratory Animals published by US National Institute of Health (NIH publication No. 83-23, revised 1996)".

Results were displayed as ED_{50} and 95% confidence limit calculated for each group using dose-response curves.

4.3. Docking studies

Molecular Operating Environment (MOE 2018.0802) software[66][67]https://www.chemcomp.com/Research-Citing_MOE.htm, Chemical Computing Group (Chemical Computing Group, Quebec, Canada, Montreal, Canada) was utilized in performing Molecular docking studies. The crystal structure of COX-2 enzyme in complex with celecoxib (PDB code 3LN1) was downloaded from RCSB Protein Data Bank website https://www.rcsb.org/. Preparation of the chosen compounds was performed through hydrogens addition, calculation of partial charges, together with energy minimization using MMFF94x Force Field at thoroughness = 0.01. The targeted protein was prepared by removing repeated chains, and water molecules. MOE QuickPrep protocol was employed in order to optimize the structural issues through 3D protonation and calculation of partial charges (RMSD gradient = 0.01 kcal/mol, AMBER10: EHT field)[68]. The best poses and binding score values of the selected compounds were detected by applying procedure and parameters in the MOE Dock protocol. Firstly, the co-crystallized ligand, celecoxib was extracted from the active site and then re-docked into COX-2 active sites to evaluate the docking reliability, and then molecular docking was carried out for the selected compounds using the MOE default settings, utilizing triangle matcher as placement method and London dG as the main scoring function. Induced fit (PDB code 3LN1) and Rigid Receptor (PDB code 3TZF) methods with affinity dG scoring function were also applied as an extra refinement step. Moreover, the obtained docking poses of the tested compounds were assessed according to the binding energy scores; RMSD values; conformity of the binding forces with the co-crystalized ligand, celecoxib. Finally, the best pose was isolated, placed in the active site, and saved as a picture to get it exported as JPEG file.

4.4. *In silico* prediction of physicochemical properties, drug likeness score, pharmacokinetics, toxicity profile and ligand efficiency metrics

In the present study, prediction of the physicochemical properties was performed using Molinspiration chemoinformatic server, pharmacokinetics by Pre-ADMET calculator, drug likeness score and toxicological effects by Osiris property explorer.

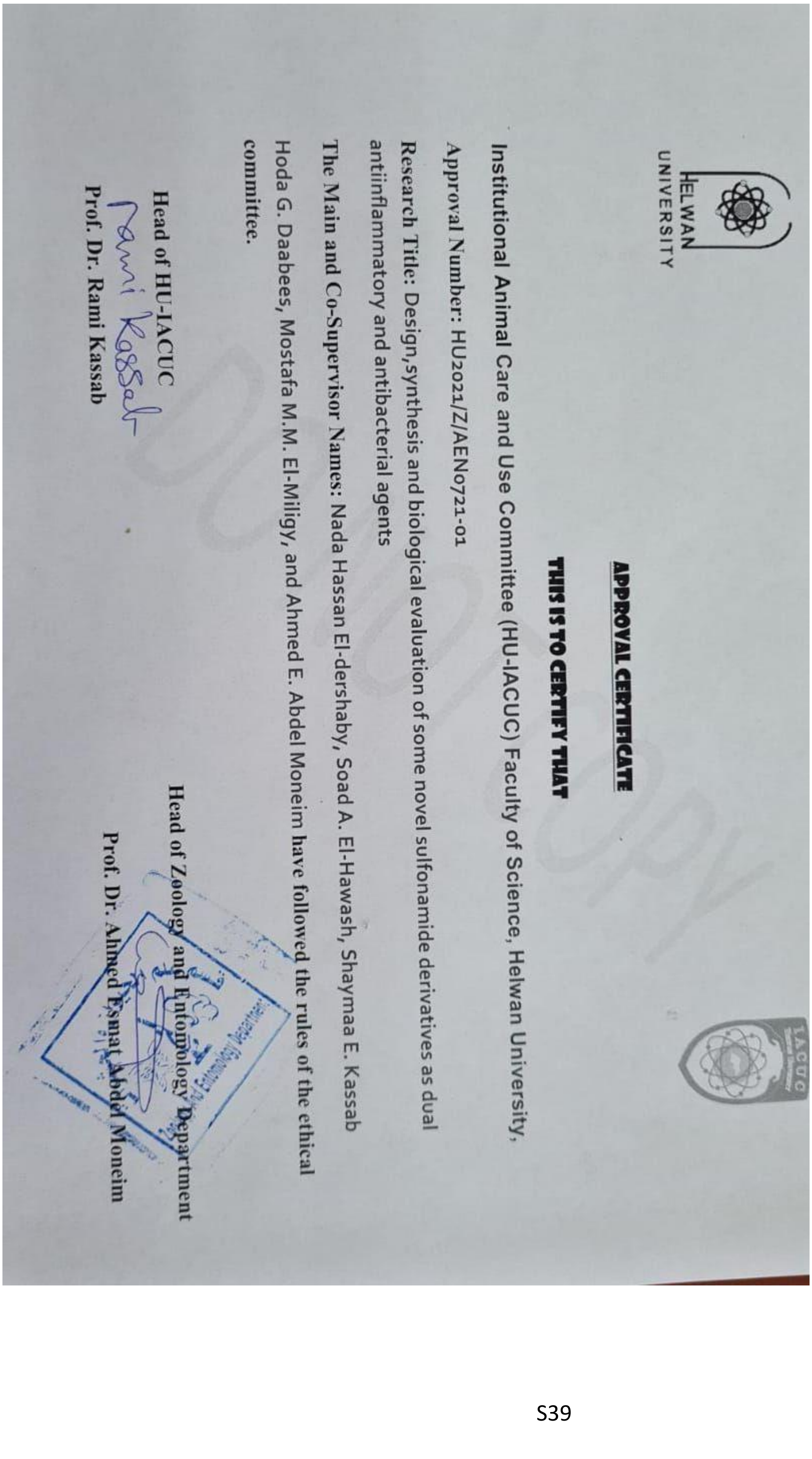

HELWAN UNIVERSITY

APPROVAL CERTIFICATE

THIS IS TO CERTIFY THAT

Institutional Animal Care and Use Committee (HU-IACUC) Faculty of Science, Helwan University,

Approval Number: HU2021/Z/AEN0721-01

Research Title: Design,synthesis and biological evaluation of some novel sulfonamide derivatives as dual antiinflammatory and antibacterial agents

The Main and Co-Supervisor Names: Nada Hassan El-dershaby, Soad A. El-Hawash, Shaymaa E. Kassab

Hoda G. Daabees, Mostafa M.M. El-Miligy, and Ahmed E. Abdel Moneim have followed the rules of the ethical committee.

Head of HU-IACUC
Prof. Dr. Rami Kassab

Head of Zoology and Entomology Department
Prof. Dr. Ahmed Esmat Abdel Moneim

References:

[1] N.Ö. Can, D. Osmaniye, S. Levent, B.N. Sağlık, B. Korkut, Ö. Atlı, Y. Özkay, Z.A. Kaplancıklı, Design, synthesis and biological assessment of new thiazolylhydrazine derivatives as selective and reversible hMAO-A inhibitors, Eur. J. Med. Chem. 144 (2018) 68–81. https://doi.org/10.1016/j.ejmech.2017.12.013.

[2] Molinspiration Cheminformatics, n.d.https://www.molinspiration.com/.

[3] Pre-ADMET, n.d.https://preadmet.bmdrc.kr/.

[4] Osiris Property Explorer, n.d.https://www.organic-chemistry.org/prog/peo/.

[5] Y.H. Zhao, M.H. Abraham, J. Le, A. Hersey, C.N. Luscombe, G. Beck, B. Sherborne, I. Cooper, Rate-limited steps of human oral absorption and QSAR studies., Pharm. Res. 19 (2002) 1446–1457. https://doi.org/10.1023/a:1020444330011.

[6] M.J. Ahsan, J. Govindasamy, H. Khalilullah, G. Mohan, J.P. Stables, C. Pannecouque, E. De Clercq, POMA analyses as new efficient bioinformatics' platform to predict and optimise bioactivity of synthesized 3a,4-dihydro-3H-indeno[1,2-c]pyrazole-2-carboxamide/carbothioamide analogues, Bioorg. Med. Chem. Lett. 22 (2012) 7029–7035. https://doi.org/https://doi.org/10.1016/j.bmcl.2012.09.108.

[7] N.W. Hassan, M.N. Saudi, Y.S. Abdel-Ghany, A. Ismail, P.A. Elzahhar, D. Sriram, R. Nassra, M.M. Abdel-Aziz, S.A. El-Hawash, Novel pyrazine based anti-tubercular agents: Design, synthesis, biological evaluation and in silico studies, Bioorg. Chem. 96 (2020) 103610. https://doi.org/https://doi.org/10.1016/j.bioorg.2020.103610.

[8] P.A. Elzahhar, S.M. Abd El Wahab, M. Elagawany, H. Daabees, A.S.F. Belal, A.F. EL-Yazbi, A.H. Eid, R. Alaaeddine, R.R. Hegazy, R.M. Allam, M.W. Helmy, Bahaa Elgendy, A. Angeli, S.A. El-Hawash, C.T. Supuran, Expanding the anticancer potential of 1,2,3-triazoles via simultaneously targeting Cyclooxygenase-2, 15-lipoxygenase and tumor-associated carbonic anhydrases, Eur. J. Med. Chem. 200 (2020) 112439. https://doi.org/10.1016/j.ejmech.2020.112439.

[9] P.W. Kenny, A. Leitão, C.A. Montanari, Ligand efficiency metrics considered harmful, J. Comput. Aided. Mol. Des. 28 (2014) 699–710. https://doi.org/10.1007/s10822-014-9757-8.

[10] A.L. Hopkins, C.R. Groom, A. Alex, Ligand efficiency: a useful metric for lead selection, Drug Discov. Today. 9 (2004) 430–431. https://doi.org/https://doi.org/10.1016/S1359-6446(04)03069-7.

[11] A.L. Hopkins, G.M. Keserü, P.D. Leeson, D.C. Rees, C.H. Reynolds, The role of ligand efficiency metrics in drug discovery, Nat. Rev. Drug Discov. 13 (2014) 105–121. https://doi.org/10.1038/nrd4163.
